# Supplementary material for: Association between surgical volume and failure of primary total hip replacement in England and Wales: findings from a prospective national joint replacement register
Source: BMJ Open. 2020 Sep 14;10(9):e033045. doi: 10.1136/bmjopen-2019-033045 (PMC7490953; doi:10.1136/bmjopen-2019-033045)
Supplement: Supplementary data [file bmjopen-2019-033045supp001.pdf]

Supplementary Tables

Multilevel Weibull model

Denote by V_{ij} the volume in the 365 days prior to procedure i ($i = 1, \dots, I$) carried out by surgeon j ($j = 1, \dots, J$) and $\bar{V}_j$ the surgeon's average volume over the observation period. A multilevel Weibull model for the association between $h_{ij}(t)$, the hazard of revision at time t for procedure i of surgeon j, and volume is specified as

$$h_{ij}(t) = h_0(t)\exp\left[\boldsymbol{\alpha} S(\bar{V}_j, K_0) + \boldsymbol{\gamma} S\left((\bar{V}_j - V_{ij}), K_1\right) + \boldsymbol{\beta} X_{ij} + Z_j\right]$$

where $h_0(t)$ is the baseline hazard function of a Weibull distribution, $S()$ represents the restricted cubic spline basis, with a vector of K_k knots, $\boldsymbol{\alpha}$ is a vector of coefficients representing the between-consultant volume association, $\boldsymbol{\gamma}$ is a vector of coefficients for the within-consultant volume association, and $\boldsymbol{\beta}$ is a vector of coefficients for confounding factors X_{ij}, and $Z_j \sim N(0, \sigma^2)$ is a surgeon-level random effect.

Supplementary.Table 1: Coding and specification of confounding variables

Variable	Options	Baseline | Centred	Regression Specification
Patient Age	Orthogonalized Restricted Cubic Splines Knots @ knots(50(10)90 102	70	Continuous
Sex	Male / Female	Male	Binary
American Society of Anaesthesiologists (ASA) grade	I / II / III / IV+V	II	MV Binary
Operation funder	Public / Private	Public	Binary
Fixation	Cemented / Uncemented / Hybrid / Reverse Hybrid	Cemented	MV Binary
Approach	Posterior /Other	Posterior	Binary
Patient position during arthroplasty	Lateral / Supine	Lateral	Binary
Anaesthetic type	Spinal / GA / GA & Spinal / GA & Nerve Block/ Epidural / Nerve Block & Spinal / GA & Epidural / None / Epidural & Spinal / Nerve Block / GA & Nerve Block & Spinal / Other	Spinal	MV Binary
Mechanical thromboprophylaxis	TED / TED ICC / ICC / TED FP / FP / TED FP ICC / FP ICC / None	None	MV Binary
Chemical thromboprophylaxis	LMWH / Asprin/ DTI / LMWH Asprin / FXa / LMWH DTI / LMWH FXa / PS / LMWH Warf / Warf / LDH / LMWH PS /None /Other	None	MV Binary
Bearing	CoC / CoM /CoPe /MoC / MoPe / MoPu	MoPE	MV Binary
Year of arthroplasty	Orthogonalised Restricted Cubic Splines Knots @ 0(4.33)13	2004	Continuous
Setting of the treatment	NHS / Treatment Centre / Privates	NHS	MV Binary
Surgical centre volume	Orthogonalised Restricted Cubic Splines Knots @ 0(430)1720	97	Continuous

Training status of the primary surgeon	Consultant / Trainee	Consultant	Binary
Consultant was listed in the NJR prior to 2008	<2008 / >= 2008	<2008	Binary
proportion of THA undertaken in the NHS	Orthogonalized Restricted Cubic Splines Knots @ knots(0(0.25)1	0.7	Continuous
proportion of THA procedures	Orthogonalized Restricted Cubic Splines Knots @ knots(0(0.125)1	0.6	Continuous
Index of Multiple Deprivation (English)	Deciles	Decile #1	MV Binary
Welsh Index of Multiple Deprivation	Deciles	Decile #1	MV Binary

Supplementary.Table 2: Descriptive statistics of continuous variables used in the analysis.

Variable	Mean	(SD)	[25th	50th	75th]	Missing
(Traced) Age at operation date, rounded 2 DP	69.84	(10.2)	[63.56,	70.65,	77.16]	0
Consultant recorded hip volume in previous 365 days	118.36	(97.3)	[52.00,	95.00,	158.00]	0
SD consultant recorded hip volume in previous 365 days by consultant	28.13	(19.8)	[14.48,	23.48,	36.53]	114
Centre recorded hip volume in previous 365 days	370.18	(306.2)	[174.00,	284.00,	435.00]	0
SD centre recorded hip volume in previous 365 days by centre	81.84	(57.5)	[44.69,	65.90,	101.86]	2
Proportion of NHS hip procedures in previous 365 days	0.69	(0.3)	[0.54,	0.74,	0.94]	0
SD Proportion of NHS hip procedures in previous 365 day by consultant	0.10	(0.1)	[0.05,	0.10,	0.14]	114
Proportion of hip procedures in previous 365 days	0.60	(0.2)	[0.46,	0.58,	0.73]	0
SD Proportion of hip procedures in previous 365 days by consultant	0.06	(0.0)	[0.04,	0.06,	0.08]	114

Supplementary.Table 3: Descriptive statistics of categorical variable used in the analysis.

Variable	Factor	N	(%)
Gender	Female	397107	(61.1)
	Male	252852	(38.9)
	Missing	0	(0.0)
ASA Grade	I	94042	(14.5)
	II	457780	(70.4)
	III	95293	(14.7)
	IV +V	2844	(0.4)
	Missing	0	(0.0)
Procedure funded by:	NHS	546727	(84.1)
	Private	88588	(13.6)
	Missing	14644	(2.3)
Method of fixation	Cemented	236554	(36.4)
	Uncemented	260857	(40.1)
	Hybrid	133489	(20.5)
	Reverse Hybrid	19059	(2.9)
	Missing	0	(0.0)
Surgical Approach	Posterior	376818	(58.0)
	Other	273141	(42.0)
	Missing	0	(0.0)
Patient Position	Lateral	595964	(91.7)
	Supine	53995	(8.3)
	Missing	0	(0.0)
Anaesthetic combination	Sp	329954	(50.8)
	GA	141132	(21.7)
	GA Sp	79706	(12.3)
	GA NB	29658	(4.6)
	Epi	18759	(2.9)
	NB Sp	16450	(2.5)
	GA Epi	13235	(2.0)
	None	103	(0.0)
	Epi Sp	5449	(0.8)
	NB	3912	(0.6)
	GA NB Sp	2353	(0.4)
	Other	600	(0.1)
	Missing	8648	(1.3)
Mechanical thromboprophylaxis combination	TED	198597	(30.6)
	TED ICC	128881	(19.8)
	ICC	101050	(15.5)
	TED FP	77150	(11.9)
	FP	58467	(9.0)
	None	33814	(5.2)
	TED FP ICC	26230	(4.0)
	FP ICC	8994	(1.4)
	Missing	16776	(2.6)
Chemical thromboprophylaxis combination	LMWH	397523	(61.2)
	None	57057	(8.8)
	Asprin	44497	(6.8)
	DTI	36394	(5.6)
	LMWH Asprin	29615	(4.6)
	FXa	31768	(4.9)
	LMWH DTI	14628	(2.3)
	LMWH FXa	7391	(1.1)
	PS	4875	(0.8)

	LMWH Warf	3712	(0.6)
	Warf	3264	(0.5)
	LDH	1299	(0.2)
	LMWH PS	1781	(0.3)
	Other	1364	(0.2)
	Missing	14791	(2.3)
Bearing Surface [Head on Acetab.]	CoC	108539	(16.7)
	CoM	1916	(0.3)
	CoPe	119379	(18.4)
	MoC	115	(0.0)
	MoPe	419844	(64.6)
	MoPu	166	(0.0)
	Missing	0	(0.0)
Hospital provider category	NHS	420897	(64.8)
	Treatment Centre	27233	(4.2)
	Private	201829	(31.1)
	Missing	0	(0.0)
Year of operation	04	3783	(0.6)
	05	22638	(3.5)
	06	29726	(4.6)
	07	38141	(5.9)
	08	42083	(6.5)
	09	46407	(7.1)
	10	52725	(8.1)
	11	57465	(8.8)
	12	63544	(9.8)
	13	66043	(10.2)
	14	71634	(11.0)
	15	71316	(11.0)
	16	72761	(11.2)
	17	11693	(1.8)
	Missing	0	(0.0)
Operation training type (Consultant / Lead surgeon / Assisted by) Number in parentheses indicates the same or different consultant	Con.(1)/Con.(1)	520620	(80.1)
	Con.(1)/Con.(1)(?)	18202	(2.8)
	Con.(1)/Con.(2)	15041	(2.3)
	Con.(1)/Jun.(1)/Con.	37414	(5.8)
	Con.(1)/Jun.(1)/Jun.(2)	58682	(9.0)
	Missing	0	(0.0)
Consultant first recorded procedure post 2008 + 365days	Cons. Pre 2009	547791	(84.3)
	Cons. Post 2009	102168	(15.7)
	Missing	0	(0.0)
Index of Multiple Deprivation Deciles (Wales)	1	1945	(0.3)
	2	2460	(0.4)
	3	2854	(0.4)
	4	3183	(0.5)
	5	3668	(0.6)
	6	4351	(0.7)
	7	4596	(0.7)
	8	4498	(0.7)
	9	4294	(0.7)
	10	3630	(0.6)
	Missing	614480	(94.5)
Index of Multiple Deprivation Deciles (England)	1	32697	(5.0)
	2	37030	(5.7)
	3	43721	(6.7)
	4	54200	(8.3)
	5	63862	(9.8)
	6	71498	(11.0)
	7	74777	(11.5)

	8	76272	(11.7)
	9	78245	(12.0)
	10	80544	(12.4)
	Missing	37113	(5.7)

Supplementary.Table 4: Consultant volume summary statistics by potential confounding factors.

Variable	Category	N	Mean	(SD)	[25th	50th	75th]
Age at operation	50 to 55yrs	28129	129.82	(97.4)	[64.00,	108.00,	170.00]
	55 to 60yrs	48289	124.41	(95.9)	[59.00,	103.00,	164.00]
	60 to 65yrs	78757	121.97	(97.5)	[55.00,	99.00,	163.00]
	65 to 70yrs	109204	120.88	(98.6)	[53.00,	97.00,	161.00]
	70 to 75yrs	116901	118.50	(97.9)	[51.00,	95.00,	158.00]
	75 to 80yrs	104115	117.07	(98.9)	[50.00,	93.00,	156.00]
	80 to 85yrs	64385	117.64	(99.4)	[50.00,	93.00,	157.00]
	85 to 90yrs	25244	119.65	(101.7)	[51.00,	94.00,	159.00]
	>90yrs	4834	123.76	(105.2)	[53.00,	97.00,	165.00]
Gender	Female	355154	120.68	(99.3)	[53.00,	97.00,	161.00]
	Male	224704	119.45	(96.9)	[53.00,	96.00,	159.00]
ASA Grade	I	75489	129.62	(105.2)	[56.00,	103.00,	176.00]
	II	414552	120.68	(98.9)	[53.00,	97.00,	160.00]
	III	87278	110.27	(89.1)	[49.00,	88.00,	148.00]
	IV +V	2539	103.76	(80.5)	[46.00,	83.00,	140.00]
Procedure funded by:	NHS	500193	116.38	(94.5)	[52.00,	94.00,	155.00]
	Private	79665	144.25	(117.2)	[63.00,	116.00,	196.00]
Method of fixation	Cemented	209831	100.24	(73.9)	[43.00,	82.00,	140.00]
	Uncemented	231302	123.73	(100.1)	[56.00,	96.00,	160.00]
	Hybrid	121293	149.75	(124.2)	[71.00,	123.00,	192.00]
	Reverse Hybrid	17432	108.24	(68.9)	[57.00,	93.50,	147.00]
Hospital provider category	NHS	371858	113.06	(93.7)	[50.00,	90.00,	151.00]
	Treatment Centre	25296	103.59	(56.0)	[60.00,	100.00,	134.00]
	Private	182704	137.05	(109.5)	[61.00,	109.00,	184.00]
P(NHS) in previous 365-days	<0.25	59325	115.76	(109.8)	[42.00,	85.00,	145.00]
	>=0.25 & <0.5	70297	149.07	(118.0)	[60.00,	115.00,	212.00]
	>=0.5 & <0.75	170720	137.44	(100.4)	[72.00,	117.00,	178.00]
	>=0.75 & <1	278820	103.62	(84.7)	[45.00,	83.00,	140.00]
P(Hip Arthroplasty) in previous 365-days	<0.25	21128	25.62	(21.4)	[10.00,	21.00,	33.00]
	>=0.25 & <0.5	176666	66.41	(40.2)	[36.00,	59.00,	89.00]
	>=0.5 & <0.75	255607	119.99	(67.8)	[69.00,	109.00,	159.00]
	>=0.75 & <1	126204	212.03	(137.2)	[123.00,	188.00,	265.00]
Index of Multiple Deprivation Deciles (Wales)	1	1657	98.69	(78.0)	[39.00,	72.00,	148.00]
	2	2153	98.57	(74.4)	[40.00,	76.00,	142.00]
	3	2522	91.51	(67.8)	[40.00,	71.00,	129.00]
	4	2809	88.34	(68.7)	[38.00,	69.00,	121.00]
	5	3343	91.71	(72.2)	[36.00,	70.00,	130.00]
	6	3961	90.30	(67.8)	[38.00,	70.00,	130.00]
	7	4249	97.31	(70.0)	[43.00,	77.00,	138.00]
	8	4135	98.71	(70.1)	[44.00,	80.00,	139.00]

	9	3947	115.23	(84.7)	[49.00,	92.00,	174.00]
	10	3265	128.15	(85.1)	[56.00,	108.00,	198.00]
Index of Multiple Deprivation Deciles (England)	1	28022	98.15	(75.1)	[45.00,	81.00,	131.00]
	2	32304	101.47	(81.3)	[46.00,	83.00,	135.00]
	3	38370	106.67	(89.1)	[47.00,	85.00,	140.00]
	4	47978	111.39	(88.7)	[51.00,	90.00,	147.00]
	5	57129	118.02	(97.2)	[53.00,	94.00,	155.00]
	6	64237	124.51	(101.9)	[56.00,	100.00,	164.00]
	7	67491	125.95	(103.4)	[57.00,	101.00,	166.00]
	8	68831	127.19	(103.2)	[57.00,	103.00,	170.00]
	9	70537	130.69	(105.0)	[59.00,	106.00,	174.00]
	10	72918	134.51	(108.8)	[60.00,	111.00,	179.00]
Surgical Approach	Posterior	342849	144.30	(108.1)	[71.00,	121.00,	189.00]
	Other	237009	85.35	(68.7)	[37.00,	69.00,	115.00]
Patient Position	Lateral	535280	122.45	(99.0)	[55.00,	99.00,	164.00]
	Supine	44578	93.27	(87.1)	[40.00,	70.00,	112.00]
Anaesthetic combination	Sp	305186	116.39	(88.7)	[56.00,	98.00,	154.00]
	GA	125359	109.36	(87.5)	[46.00,	86.00,	151.00]
	GA Sp	71916	162.93	(131.8)	[68.00,	128.00,	221.00]
	GA NB	25213	105.09	(83.1)	[44.00,	84.00,	144.00]
	Epi	16568	95.02	(84.9)	[38.00,	71.00,	132.00]
	NB Sp	15451	132.51	(151.0)	[53.00,	86.00,	137.00]
	GA Epi	9820	130.50	(98.4)	[52.00,	102.00,	202.00]
	None	59	115.80	(78.9)	[49.00,	103.00,	167.00]
	Epi Sp	4041	104.14	(79.2)	[43.00,	83.00,	155.00]
	NB	3524	101.01	(83.8)	[48.00,	79.00,	133.00]
	GA NB Sp	2188	163.59	(115.9)	[78.00,	149.00,	230.00]
	Other	533	111.07	(112.9)	[44.00,	85.00,	143.00]
Mechanical thromboprophylaxis combination	TED	183510	115.60	(104.1)	[49.00,	92.00,	149.00]
	TED ICC	119415	118.20	(101.3)	[51.00,	90.00,	151.00]
	ICC	92340	109.16	(77.2)	[52.00,	91.00,	151.00]
	TED FP	67618	146.91	(118.8)	[67.00,	120.00,	187.00]
	FP	53375	132.33	(86.1)	[62.00,	122.00,	190.00]
	None	31757	117.05	(79.4)	[53.00,	100.00,	170.00]
	TED FP ICC	23445	115.61	(84.4)	[58.00,	91.00,	151.00]
	FP ICC	8398	103.40	(81.1)	[48.00,	83.00,	144.00]
Chemical thromboprophylaxis combination	LMWH	362081	113.44	(94.4)	[50.00,	91.00,	150.00]
	None	53791	129.35	(106.3)	[54.00,	99.00,	176.00]
	Asprin	38635	125.01	(88.2)	[56.00,	110.00,	170.00]
	DTI	34669	117.68	(78.4)	[59.00,	100.00,	163.00]
	LMWH Asprin	26428	146.37	(111.5)	[64.00,	125.00,	207.00]
	FXa	30026	153.06	(120.9)	[73.00,	122.00,	194.00]
	LMWH DTI	13840	138.70	(127.3)	[56.00,	99.00,	186.00]
	LMWH FXa	7019	128.35	(122.9)	[58.00,	99.00,	159.00]
	PS	4553	102.17	(64.2)	[50.00,	91.00,	144.00]
	LMWH Warf	3028	121.75	(98.5)	[55.00,	103.00,	164.00]

	Warf	2616	115.38	(73.6)	[57.00,	117.00,	152.00]
	LDH	677	91.82	(86.9)	[28.00,	47.00,	168.00]
	LMWH PS	1360	104.65	(77.3)	[49.00,	86.00,	160.00]
	Other	1135	120.09	(117.3)	[56.00,	88.00,	154.00]
Bearing Surface [Head on Acetab.]	CoC	89379	125.46	(90.5)	[61.00,	104.00,	166.00]
	CoM	1728	186.67	(141.7)	[69.00,	121.00,	286.50]
	CoPe	106359	138.64	(125.1)	[60.00,	104.00,	178.00]
	MoC	100	103.72	(88.3)	[41.50,	68.50,	140.50]
	MoPe	382139	113.55	(90.3)	[49.00,	92.00,	154.00]
	MoPu	153	123.27	(46.2)	[92.00,	130.00,	160.00]
Year of operation	04	1902	57.84	(47.1)	[24.00,	45.00,	77.00]
	05	12392	73.87	(60.3)	[29.00,	56.00,	101.00]
	06	16901	91.97	(81.6)	[33.00,	69.00,	124.00]
	07	22287	97.09	(83.3)	[36.00,	75.00,	133.00]
	08	37678	111.83	(98.2)	[44.00,	86.00,	149.00]
	09	44020	115.91	(102.8)	[45.00,	89.00,	151.00]
	10	50416	114.78	(95.7)	[48.00,	89.00,	155.00]
	11	54957	116.60	(98.2)	[50.00,	91.00,	154.00]
	12	60581	122.55	(99.0)	[55.00,	99.00,	163.00]
	13	62950	124.14	(94.3)	[58.00,	100.00,	169.00]
	14	68357	127.82	(94.1)	[63.00,	106.00,	171.00]
	15	68189	133.04	(103.8)	[64.00,	111.00,	172.00]
	16	69434	130.91	(106.2)	[65.00,	107.00,	169.00]
	17	9794	133.73	(106.7)	[67.00,	108.00,	175.00]
Centre Volume in previous 365 days	1x day	252914	99.94	(90.5)	[40.00,	75.00,	130.00]
	2x day	223425	116.00	(76.7)	[59.00,	101.00,	158.00]
	3x day	40127	142.01	(86.8)	[80.00,	127.00,	191.00]
	4x day	33584	226.83	(167.5)	[115.00,	183.00,	289.00]
	5x day	14127	198.14	(143.4)	[99.00,	172.00,	250.00]
	6x day	12630	151.66	(73.8)	[99.00,	133.00,	206.00]
	7x day	3051	156.37	(64.7)	[110.00,	142.00,	220.00]
Operation training type (Consultant / Lead surgeon / Assisted by) Number in parentheses indicates the same or different consultant	Con.(1)/Con.(1)	463705	117.46	(91.7)	[53.00,	95.00,	157.00]
	Con.(1)/Con.(1)(?)	16246	105.20	(115.9)	[40.00,	67.00,	133.00]
	Con.(1)/Con.(2)	13637	141.29	(134.4)	[49.00,	101.00,	197.00]
	Con.(1)/Jun.(1)/Con.	34006	110.47	(72.3)	[56.00,	101.00,	153.00]
	Con.(1)/Jun.(1)/Jun.(2)	52264	150.02	(139.3)	[58.00,	119.00,	191.00]
Consultant first recorded procedure post 2008 + 365days	Cons. Pre 2009	482572	126.19	(104.1)	[54.00,	101.00,	170.00]
	Cons. Post 2009	97286	90.53	(53.7)	[50.00,	81.00,	122.00]

Supplementary.Figure 1 ***Between-consultant*** *Marginal association of hip surgical volume in the preceding 365-days and hazard of revision arthroplasty adjusted with 4 different sets (M2 to M4) of confounding factors in a multi-level model (MLM).*

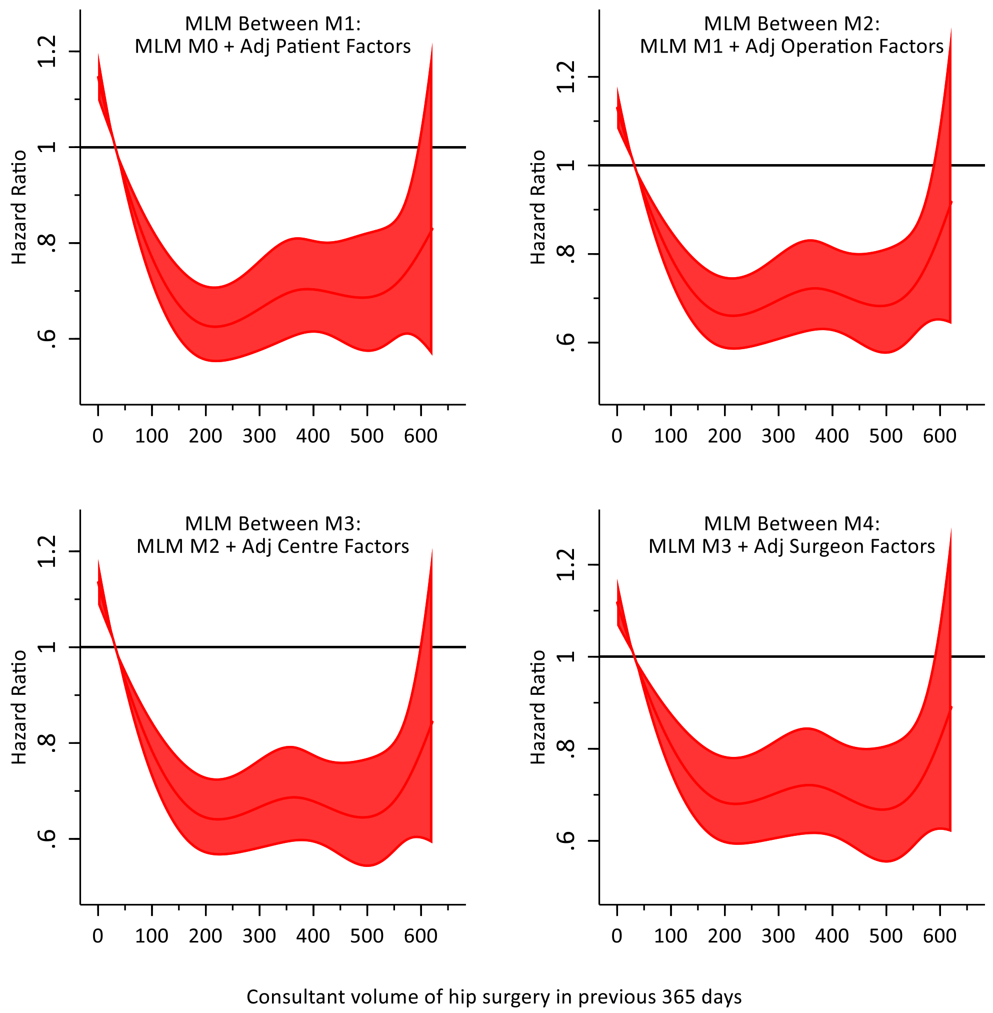

Foot Note.

Patient factors include sex, ASA grade, funder. Operation confounding factors include fixation, approach, position, anaesthetic, mechanical and chemical thromboprophylaxis, bearing, and year of operation. Centre confounding factors include hospital location and centre volume in the preceding 365-days. Surgeon confounding factors included; lead

operating surgeon, listing of a surgeon within NJR prior to 2008, the proportion of NHS cases in the preceding year, and proportion hip arthroplasty procedures undertaken in the previous year.

Supplementary.Figure 2 ***Within-consultant*** *Marginal association of hip surgical volume in the preceding 365-days and hazard of revision arthroplasty adjusted with 4 different sets (M2 to M4) of confounding factors in a multi-level model (MLM).*

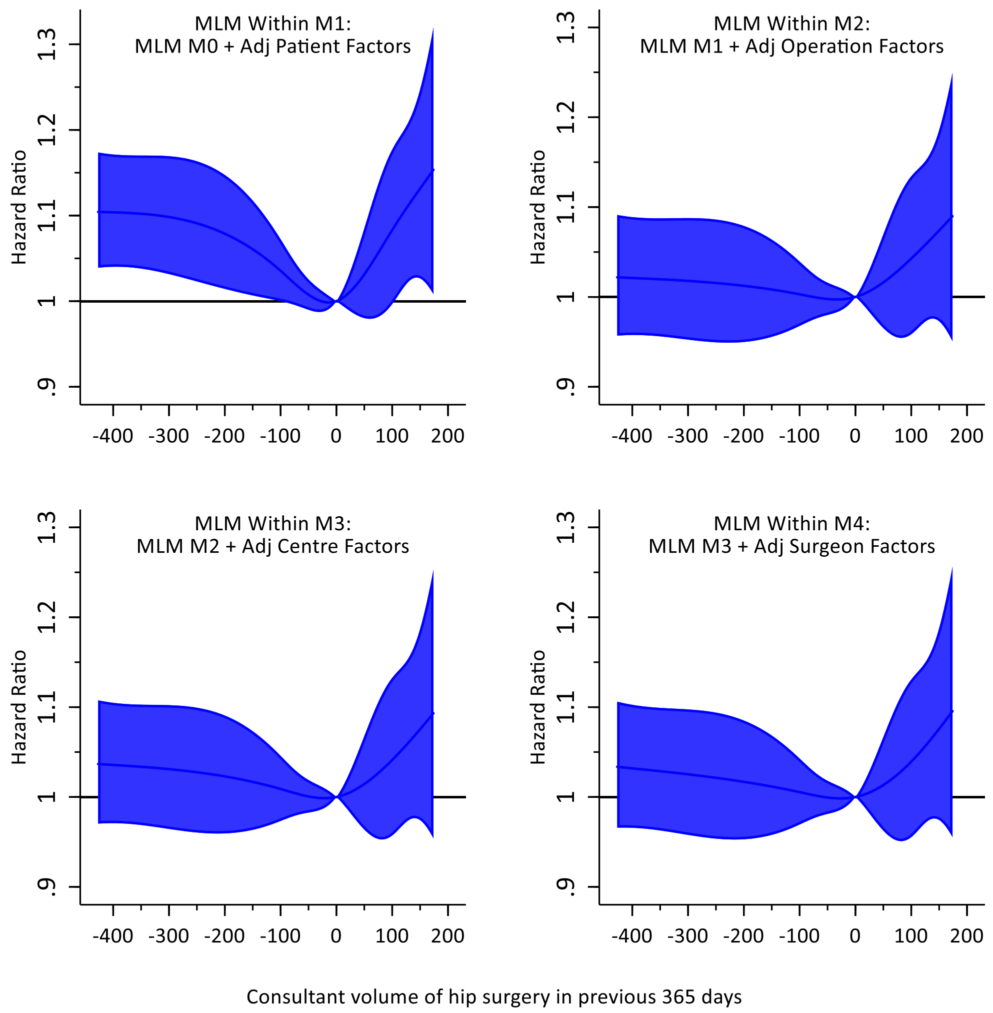

Foot Note.

Patient factors include sex, ASA grade, funder. Operation confounding factors include fixation, approach, position, anaesthetic, mechanical and chemical thromboprophylaxis, bearing, and year of operation. Centre confounding factors include hospital location and

centre volume in the preceding 365-days. Surgeon confounding factors included; lead operating surgeon, listing of a surgeon within NJR prior to 2008, the proportion of NHS cases in the preceding year, and proportion hip arthroplasty procedures undertaken in the previous year.
