# Supplementary material for: Association between surgical volume and failure of primary total hip replacement in England and Wales: findings from a prospective national joint replacement register
Source: BMJ Open. 2020 Sep 14;10(9):e033045. doi: 10.1136/bmjopen-2019-033045 (PMC7490953; doi:10.1136/bmjopen-2019-033045)
Supplement: Author's manuscript [file bmjopen-2019-033045.draft_revisions.pdf]

BMJ Open is committed to open peer review. As part of this commitment we make the peer review history of every article we publish publicly available.

When an article is published we post the peer reviewers' comments and the authors' responses online. We also post the versions of the paper that were used during peer review. These are the versions that the peer review comments apply to.

The versions of the paper that follow are the versions that were submitted during the peer review process. They are not the versions of record or the final published versions. They should not be cited or distributed as the published version of this manuscript.

BMJ Open is an open access journal and the full, final, typeset and author-corrected version of record of the manuscript is available on our site with no access controls, subscription charges or pay-per-view fees (http://bmjopen.bmj.com).

If you have any questions on BMJ Open's open peer review process please email info.bmjopen@bmj.com

The association between surgical volume and failure of primary total hip replacement in England and Wales: Findings from the National Joint Registry of England and Wales.

Journal:	*BMJ Open*
Manuscript ID	bmjopen-2019-033045
Article Type:	Original research
Date Submitted by the Author:	17-Jul-2019

Complete List of Authors:	Sayers, Adrian; University of Bristol, Muscloskeletal Research Unit<br>Steele, Fiona; LSE, Department of Statistics<br>Whitehouse, Michael; University of Bristol, Musculoskeletal Research Unit<br>Price, Andrew; University of Oxford, Nuffield Department of Orthopaedics, Rheumatology and Musculoskeletal Sciences<br>Ben-Shlomo, Yoav; University of Bristol,<br>Blom, AW; University of Bristol,

Keywords:	Total Hip Arthroplasty, Hip < ORTHOPAEDIC & TRAUMA SURGERY, volume, Revision surgery, Within Surgeon Effects, Between Surgeon effects

SCHOLARONE™
Manuscripts

I, the Submitting Author has the right to grant and does grant on behalf of all authors of the Work (as defined in the below author licence), an exclusive licence and/or a non-exclusive licence for contributions from authors who are: i) UK Crown employees; ii) where BMJ has agreed a CC-BY licence shall apply, and/or iii) in accordance with the terms applicable for US Federal Government officers or employees acting as part of their official duties; on a worldwide, perpetual, irrevocable, royalty-free basis to BMJ Publishing Group Ltd ("BMJ") its licensees and where the relevant Journal is co-owned by BMJ to the co-owners of the Journal, to publish the Work in this journal and any other BMJ products and to exploit all rights, as set out in our licence.

The Submitting Author accepts and understands that any supply made under these terms is made by BMJ to the Submitting Author unless you are acting as an employee on behalf of your employer or a postgraduate student of an affiliated institution which is paying any applicable article publishing charge ("APC") for Open Access articles. Where the Submitting Author wishes to make the Work available on an Open Access basis (and intends to pay the relevant APC), the terms of reuse of such Open Access shall be governed by a Creative Commons licence – details of these licences and which Creative Commons licence will apply to this Work are set out in our licence referred to above.

Other than as permitted in any relevant BMJ Author's Self Archiving Policies, I confirm this Work has not been accepted for publication elsewhere, is not being considered for publication elsewhere and does not duplicate material already published. I confirm all authors consent to publication of this Work and authorise the granting of this licence.

For peer review only

Full Title: The association between surgical volume and failure of primary total hip replacement in England and Wales: Findings from the National Joint Registry of England and Wales.

Short Title: Surgical volume and failure of primary total hip replacement

Adrian Sayers[1,2], Fiona Steele[3], Michael R Whitehouse[1,5], Andrew Price[4], Yoav Ben-Shlomo[2], Ashley W Blom[1,5]

1. Musculoskeletal Research Unit, Bristol Medical School, 1st Floor Learning & Research Building, Southmead Hospital, Bristol, BS10 5NB, United Kingdom
2. Population Health Sciences, Bristol Medical School, Canynge Hall, 39 Whatley Road Bristol, BS8 2PS, United Kingdom
3. Department of Statistics, London School of Economics and Political Science, London, UK
4. NIHR Biomedical Research Unit, University of Oxford, Oxford, UK
5. National Institute for Health Research Bristol Biomedical Research Centre, University Hospitals Bristol NHS Foundation Trust and University of Bristol

Corresponding Author

Adrian Sayers

Musculoskeletal Research Unit, Bristol Medical School, 1st Floor Learning & Research Building, Southmead Hospital, Bristol, BS10 5NB, United Kingdom

Email: adrian.sayers@bristol.ac.uk Tel: 0117 4147880

Word Count:2826

For peer review only

For peer review only - http://bmjopen.bmj.com/site/about/guidelines.xhtml

Abstract

Objective

Investigate the association of volume of total hip arthroplasty (THA) between consultants and within the same consultant in the previous year and the hazard or revision using multi-level survival models.

Design

Prospective cohort study using data from a national joint replacement register.

Setting

Elective total hip arthroplasty (THA) across all private and public centres in England and Wales between April 2003 and February 2017.

Participants

Patients age 50 years or more undergoing THA for osteoarthritis.

Intervention

The volume of THA conducted in the preceding 365-days to the index procedure.

Main Outcome and measure

Revision surgery (excision, addition or replacement) of a primary THA.

Results

Of the 579,858 patients undergoing primary THA (mean baseline age 69.8 years [SD 10.2]), 61.1% were female. Multi-level survival found differing results for between and within-consultant effects. There was a strong volume-revision association between consultants, with a near linear 43.3% (95% CI 29.1%, 57.4%) reduction of the risk of revision comparing consultants with volumes between 1 and 200 procedures annually. Changes in individual

For peer review only - http://bmjopen.bmj.com/site/about/guidelines.xhtml

surgeons (within-consultant) case volume showed no evidence of an association with revision.

Conclusion

Separation of between- and within-consultant effects of surgical volume reveals how volume contributes to the risk of revision after THA. The lack of association within-consultants suggests that individual changes to consultant volume alone will have little effect on outcomes following THA.

These novel findings provide strong evidence supporting the practice of specialisation of hip arthroplasty. It does not support the practice of low volume consultants increasing their personal volume as it is unlikely their results would improve if this is the only change. Limiting the exposure of patients to consultants with low volumes of THA and greater utilization of centres with higher volume surgeons with better outcomes may be beneficial to patients.

Strengths and limitations of this study

- This is the largest study in the world to explore the association between surgical volume and outcomes in total hip arthroplasty
- We uniquely calculate a time-varying exposure of surgical volume.
- We differentiate between- and within- consultant effects using a multi-level Weibull survival model.
- The effect of volume is modelled continuously using restricted cubic splines.
- We are unable to affirm causality due to the observational nature of the data

Funding Statement

AS is funded by an MRC Strategic Skills Fellowship MR/L01226X/1

For peer review only - http://bmjopen.bmj.com/site/about/guidelines.xhtml

This study was supported by the NIHR Biomedical Research Centre at University Hospitals Bristol NHS Foundation Trust and the University of Bristol. The views expressed in this publication are those of the author(s) and not necessarily those of the NHS, the National Institute for Health Research or the Department of Health and Social Care.

Competing interests statement

We declare no competing interests

For peer review only

Keywords

Total Hip Arthroplasty, Volume, Revision, Within surgeon effects, Between surgeon effects.

Introduction

Centralisation and specialisation in medical care are advocated to optimise a theorised volume-outcome relationship. In arthroplasty, the volume-outcome relationship has been investigated with respect to outcomes including surgical revision,[1-6] mortality,[1, 6-9] patient reported outcome measures (PROMS),[10] and complications[6, 11-14] where volume is measured either by surgeon[1-6] or hospital annual volume.[15] Given the technical requirements of arthroplasty, a strong argument for specialisation based on surgical volume and the risk of revision arthroplasty would exist if volume was causally related to outcome. The evidence to support this assertion is surprisingly sparse and has methodological limitations.[1-6] The principal limitation is the failure to distinguish between- and within-consultant effects.

Differentiating between between- and within-consultant effects is crucial to interpreting the data. A between-consultant effect is essentially a cross-sectional analysis that compares the performance of one consultant against another and is highly likely to be confounded by centre level effects.[16] A within-consultant effect is based on a time series of individual data and compares changes of volume across time within the same consultant. Correspondingly, within effects can be interpreted more strongly, as the effect of changing a consultant's personal volume, assuming centre-level factors remain relatively constant over the short term analysis period.

The concept of between- and within-effects is well known in epidemiology, and the ecological fallacy is one example. For example, in a standard (single-level) regression analysis, we may observe a positive association between red meat consumption and life expectancy across several countries with differing levels of development. A between- and within-decomposition would reveal a positive between-country correlation, which is explained by the level of development, and a negative within-country association i.e.

For peer review only - http://bmjopen.bmj.com/site/about/guidelines.xhtml

individuals within a country who eat more red meat have a lower life expectancy. The decomposition of volume effects into between- and within-effects is similar i.e. between-consultant effects are explained by factors, other than volume, which are intrinsic to those consultants and the hospital where they are based ("centre effects"), whereas within-consultant effect represents the consequence of consultants individually changing their personal volume assuming that other factors remain constant.

In order to facilitate a between- and within-effects analysis, consultant volume needs to be assessed continuously across time. Allowing volume to vary over time is computationally intensive, but responds to variation in demand and capacity to deliver arthroplasty, specialisation or diversification of professional practice, and is in contrast to previous approaches.[1, 4] Additionally, as consultant volume changes, dichotomising the data using arbitrary thresholds, e.g. 12 operations per year,[3] is difficult to justify as consultants status will vary depending upon the time they are observed. Furthermore, the interpretation of the results is less likely to be distorted by the arbitrary placement of the threshold.[17, 18]

The aim of this research is to investigate the between- and within-consultant (surgeon) effect of the volume of primary total hip arthroplasty (THA) and the risk of subsequent revision.

For peer review only

Methods

Using data from the National Joint Registry of England, Wales, Northern Ireland and the Isle of Man (NJR), we investigated the association between consultant surgical volume in the year (365-days) prior to the index operation of interest, and the risk of revision in patients undergoing elective THA between 1st April 2003 and 22nd February 2017.

Data source

The NJR commenced data collection in April 2003; at inception, it was mandatory for all THA conducted in the private sector to be entered into the NJR, and from 2011 all THA procedures in the public and private sector were required to be entered into the NJR. A recent national audit of data entered into the NJR between 2014 and 2015 estimated data capture of 95% for primary THA and 91% for revision THA.

Patient and public involvement

Patient representatives sit on the committee structure of the National Joint Registry. The research priorities of the National Joint Registry are identified by this committee structure and approved by the patient representatives. Patients were not involved in the setting of the research question or the outcome measures, nor were they involved in designing or implementing this work or interpretation of the results. We are unable to disseminate results of this study directly to study participants due to the anonymous nature of the data. We plan to disseminate our findings to the National Joint Registry, via their communications team, to relevant to the provision of joint replacement and to the general population through the local and national press.

For peer review only - http://bmjopen.bmj.com/site/about/guidelines.xhtml

Inclusion/exclusion criteria

All consenting patients undergoing THA were eligible for inclusion in the analysis. Patients were included if their patient history was unique and consistent, i.e. contained no duplicates, ipsilateral revision prior to the primary, or currently held in query by the submitting unit. Due to the requirement of reliable date information, patients who were indicated to have died prior to undergoing a procedure, were more than 110 years of age, had undergone a procedure prior to their date of birth, or received a procedure prior to 2003 were excluded. Only primary THA, where the sole indication for operation was osteoarthritis (OA) with unique prosthesis combinations, were included. All metal-on-metal bearing combinations were excluded from the analysis due to the known exceptionally high failure rate in this group.[19, 20] Consultants with less than 365-days of data were excluded as were patients who were less than 50 years of age at the date of the index THA, because these cases are highly likely to be due to secondary OA. See Figure 1 for a detailed breakdown of inclusion criteria.

Primary outcome

The primary outcome of interest is all cause revision after a primary THA. Revision arthroplasty was identified by the inclusion of a revision specific data upload after a primary ipsilateral THA.

Censoring

Patients were censored following death. Death status was established by linking patients to the NHS Personal Demographic tracing service.

Primary exposure

The primary exposure of interest in this study was the consultant surgical volume of any THA recorded in the NJR in the preceding 365-days prior to the index procedure in

For peer review only - http://bmjopen.bmj.com/site/about/guidelines.xhtml

consenting patients, and prior to the application of inclusion and exclusion criteria, see Figure 1.

Confounding factors

Confounding factors were thematically organised into 5 groups:

1) Patient factors: Age, sex, American Society of Anesthesiologists (ASA) grade, and operation funder.
2) Operation factors: THA fixation, approach, patient position during arthroplasty, anaesthetic type, thromboprophylaxis regime, bearing, and year of primary THA.
3) Centre factors included the setting of the treatment episode (i.e. private or NHS hospital) and surgical centre volume.
4) Consultant based factors included the training status of the primary surgeon performing the operation, whether the responsible consultant was listed in the NJR after to 2008 (i.e. a newly qualified surgeon), the proportion of THA undertaken in the NHS in the preceding 365-days by the consultant (i.e. a public or private hospital surgeon), the proportion of THA procedures undertaken in the previous year compared to all joints recorded by the NJR (i.e. a specialist hip surgeon or a general arthroplasty surgeon).
5) Deprivation factors were based on the English and Welsh indices of multiple deprivation, an area based index of patient socioeconomic status. See Supplementary.Table 1.

Statistical analyses

Means, standard-deviations and interquartile points were used to describe continuous variables. Frequencies and percentages were used to describe categorical variables. The

For peer review only - http://bmjopen.bmj.com/site/about/guidelines.xhtml

association between confounding factors and consultant volume was explored by comparing summary statistics between levels of each factor.

The associations between consultant volume in the preceding 365-days of the index procedure and all-cause revision were explored using multi-level parametric (Weibull) survival models.

Between- and within-effects are decomposed using a process known as group mean centring.[21, 22] Group mean centring is the process of creating two new variables from the primary exposure. The first variable is the consultant specific mean volume, and the second variable is the deviation of a consultant's volume for a given procedure from their personal average (i.e. consultant-mean centred volume). The between-effect is estimated using the coefficient of a consultant's mean volume, whereas the within-effect is the coefficient based on the consultants deviation from their average.

Continuous variables were modelled used orthogonalized Restricted Cubic Splines (RCS), this also included the volume effect. We iteratively varied the number of knot points and placement strategies, in unadjusted models to optimize fit. The most parsimonious specification of RCS was selected using Akaike Information Criterion (AIC).[23]

Confounding adjustment was conducted incrementally introducing patient, operation, centre, surgeon, and finally, deprivation confounding variable groups. The effect of confounding adjustment on the primary exposure of interest was explored at each stage of the model building process. All modelling was conducted in Stata 15.1.[24]

Missing data

Given the large data set and small fraction of incomplete cases among the observed (FICO), any improvement of efficiency from multiple imputation is likely to be negligible, and a complete-cases analysis would provide unbiased results.[25, 26] Therefore, we have assumed

For peer review only - http://bmjopen.bmj.com/site/about/guidelines.xhtml

For peer review only

the reason for missingness is independent of both the outcome and primary exposure of interest.

For peer review only - http://bmjopen.bmj.com/site/about/guidelines.xhtml

Results

Between 1st April 2003 and 22nd February 2017, 1,008,619 primary and revision THA procedures were entered in the NJR. After application of inclusion and exclusion criteria, 579,858 primary THA replacement procedures were available for analysis, (Figure 1), including 9238 revised procedures, with 2.5 million years of observation time, with a maximum length of follow up of 13.14years. Patients were predominately (61.1 %) female, ASA II (70.4%), and treated in public (NHS) hospital (84.1%), had a mean age 69.8 years (SD 10.2). See Supplementary.Table 2 and Supplementary.Table 3 for full descriptive statistics of the population of patients used in the analysis.

Mean between consultant volume in the previous year was positively skewed, with only a minority (2.5%) of consultants performing in-excess of 200 procedures a year, (Figure 2). The median number of procedures conducted across all consultants, for the whole analysis period, in the year prior to an index procedure was 95 (IQR 52, 158) with substantial variability between consultants, (Figure 3). Mean within consultant volume, for the most part, was symmetrically distributed. There was substantial variability of volume within-consultants (median within-consultant SD is 23 (IQR 14.5, 36.5)), (Figure 2), and consultants with larger mean volumes tended to have greater variability, (Figure 3).

Summary statistics of consultant volume by confounding factors at the index procedure are listed in Supplementary.Table 4. Higher volume consultants were observed to treat patients who were younger, with lower ASA grade, who privately funded their THA, and received a uncemented or hybrid prostheses more frequently. They were also more likely to use a posterior approach, place patients in the lateral position, work in the private sector, predominately perform hip arthroplasty, and to treat patients from more deprived areas, (Supplementary.Table 4).

For peer review only - http://bmjopen.bmj.com/site/about/guidelines.xhtml

The effect of confounding adjustment on the volume association was explored using complete-cases in the multi-level Weibull model. The marginal between- and within-consultant effects of volume in the preceding year on hazard of revision and 95% CI are presented in Figure 4 and Figure 5 respectively.

A, near linear, 48.8% (95% CI:36.3,61.4) reduction in hazard ratio was observed in crude models for volumes between 1 and 200 procedures annually i.e. from 1.13 (95% CI 1.08, 1.18) to 0.64 (95% CI 0.56, 0.72), with a flattening of risk beyond this, noting that only ~2.5% of consultant recorded more than 200 procedures annually. Despite extensive confounding adjustment, the marginal association of the between-consultant volume effect remained remarkably consistent across the range of volumes observed. In our fully adjusted (deprivation) model, a near linear 43.3% (95% CI 29.1, 57.4) reduction in hazard ratio was observed. The marginal association of the within-consultant volume effect and revision was similarly consistent and showed no evidence (Figure 5) of an association with revision if consultants increased or decreased their volume by ± 50 procedures per year (HR ~1 and 95% CI includes zero).

For peer review only - http://bmjopen.bmj.com/site/about/guidelines.xhtml

Discussion

We provide novel insights into the volume outcome relationship of 579,858 elective THA patients using a between- and within-decomposition to analyse the effects of consultant volumes on revision. Between different consultants, the volume of arthroplasty in the previous year is associated with a near linear 49% and 43% reduction in hazard ratio, between 1 and 200 procedures with revision THA in crude and fully adjusted models respectively. Within the same consultant we demonstrate that there is no evidence of an association between volume of THA in the previous year and risk of revision.

Uniquely, we use a time-varying volume specification that facilitates the decomposition of between- and within-consultant effects. We suggest the within-consultant effect is much closer to the causal interpretation desired by many policymakers, and failure of research to recognise the difference amongst between- and within-effects may lead to erroneous policy decisions and unintended consequences.

We demonstrate that between-consultant optimal results are reached when the consultant volumes in the previous year are approximately 200 procedures. We suggest these factors are not causally related to volume, but rather due to unmeasured factors such as centre effects. There is no evidence to suggest that consultants should change their personal volume in the hope of improving their outcomes or that there is an arbitrary threshold where the outcome of results become good.

Whilst the results appear contradictory compared to previous research, the differences can be explained by the method of analysis, i.e. single-level models vs. multi-level model, and interpretation of results, i.e. separation of between- and within-consultant volume effects. Single-level analyses assume that all procedures are independent of one another, so that a low volume consultant would achieve the results of a high-volume consultant if they could

For peer review only - http://bmjopen.bmj.com/site/about/guidelines.xhtml

instantly increase their personal volume. The interpretation of a single-level is similar to that of the between-consultant interpretation. Whilst this may be an attractive interpretation for policymakers, it fails to recognise the complexity within the data and processes observed, and that there are many factors, intrinsic to each consultant and the centre or centres in which they work, that predispose them to be either low- or high-volume surgeons e.g. fellowship trained, threshold for revision, or unit organisation.

This study has a number of strengths and limitations. Strengths include: 1) our unique decomposition of between- and within-consultant which we believe provides a more actionable interpretation for policy makers. 2) Time-varying consultant volume, which is independent of the index procedure, and allows for stronger inferences. 3) OA was the only indication for arthroplasty, which we believe represents a "best case scenario", and the volume effect will only be attenuated by the inclusion of other diagnoses. 4) We demonstrate the use of RCS to model volume effects, which ensures flexibility and a smooth continuous function, emphasising the lack of any threshold in the volume effect. 5) The study is significantly, 10 times larger, than any other published study on this topic,[1-6] with a follow-up period of more than 13 years. 6) we have conducted extensive case-mix adjustment and illustrate that both between- and within-effects are insensitive to our measured confounding factors.

Despite the many strengths, this study has a number of limitations. 1) The analysis and decomposition of between- and within-consultant effects is more complex than traditional analyses assume, and requires careful interpretation. 2) Despite the independent nature of the volume of arthroplasty calculation prior to the index procedure, we still see anticipated associations, i.e. younger patients, patients with lower ASA scores, patients receiving uncemented implants and patients operated on in the private sector all tend to be treated by higher volume consultants. Consultants specialising in THA and working principally within

For peer review only - http://bmjopen.bmj.com/site/about/guidelines.xhtml

the private sector accrue higher volumes. These features suggest a strong propensity in practice to treat similar patients and raises the possibility of lagged correlation in unmeasured confounders.[27] 3) The use of a single indication for arthroplasty, namely OA, may limit the generalisability of results particularly in regard to Black or Asian ethnicities where OA is not as dominant an indication for THA.[28] 4) The use of RCS requires extensive sensitivity analyses to ensure knot points are placed optimally, and that results are not sensitive to knot placement. 5) Calculation of the time-varying volume specification of THA in the previous year is computationally intensive and requires significant parallelisation before analyses can be started. 6) Our covariates are unlikely to capture important centre differences in staffing, organization and policy in the management of THAs

We suggest the within-consultant effect from the multi-level regression is much closer to the causal interpretation required by consultants, patients, and policymakers i.e. what is the effect of changes in personal volume on the hazard of revision THA? This is not to say the between-effect is not of interest to policy makers, but to say that the between-effect suggests that there are intrinsic differences between high and low volume consultants, where higher volume consultants tend to have better outcomes, but these differences cannot be attributed to volume per se. Understanding variability between units independent of patient case mix has been explored in other registries such as the UK renal Registry.[16]

For peer review only

Conclusion

In summary, using data from the largest arthroplasty register in the world, we have demonstrated that there is no within-consultant association between surgical volume in the previous year and the risk of revision in patients undergoing primary THA for OA. Whereas there is strong evidence to suggest higher volume consultants tend to have better outcomes for reasons that are unlikely to be due to the volume of arthroplasty in the previous year per se.

For peer review only - http://bmjopen.bmj.com/site/about/guidelines.xhtml

The results from this study have profound implications for quality improvement within healthcare. Encouraging consultants to undertake a minimum number of procedures under the guise of raising standards could be counterproductive and may only serve to expose patients to increased risk of revision by low or previously low volume consultants. Centralisation and specialisation of THA in consultants who for reasons, not including volume, can undertake a greater number of procedures is likely to benefit patients and reduce the revision burden overall.

Importantly, the combined use of an independent time-varying consultant volume with multi-level (between- and within-) regression modelling allows results to be interpreted more clearly than a cross-sectional analysis of conventional observational data. Our work highlights the importance of appropriate methodology, and whilst this may fall short of being definitively causal, results from a randomised experiment are unlikely to be feasible in this population. We believe this represents the best available evidence to guide policy formulation.

List of abbreviations

THA: Total Hip Arthroplasty

PROMS: patient reported outcome measures

NJR: National Joint Registry of England, Wales, Northern Ireland and the Isle of Man

OA: Osteoarthritis

ASA: American Society of Anesthesiologists

NHS: National Health Service

RCS: Restricted Cubic Splines

AIC: Akaike Information Criterion

FICO: Fraction of incomplete cases among the observed

IQR: Interquartile Range

For peer review only - http://bmjopen.bmj.com/site/about/guidelines.xhtml

For peer review only

Author Contributions

AS, FAS, YBS, AJP, MRW, and AWB were responsible for the study design, AS conducted the data analysis. AS, FAS, YBS, AJP, MRW, and AWB were responsible for interpreting the data. AS, FAS, YBS, AJP, MRW, and AWB prepared and edited and approved the final manuscript.

Role of the funding source

The Medical Research Council or National Institute of Health Research had no role in the design and conduct of the study; collection, management, analysis, and interpretation of the data; preparation, review, or approval of the manuscript; and decision to submit the manuscript for publication

Availability of data and material

Access to the data can be made via research requests to the National Joint Registry of England, Wales, Northern Ireland and the Isle of Man. Full details can be found at http://www.njrcentre.org.uk/njrcentre/Research/Research-requests

Acknowledgements

We thank the patients and staff of all the hospitals who have contributed data to the National Joint Registry. We are grateful to the Healthcare Quality Improvement Partnership, the National Joint Registry Steering Committee, and staff at the National Joint Registry for facilitating this work. The views expressed represent those of the authors and do not necessarily reflect those of the National Joint Registry Steering Committee or Healthcare Quality Improvement Partnership, who do not vouch for how the information is presented.

For peer review only - http://bmjopen.bmj.com/site/about/guidelines.xhtml

Figure Titles

Figure 1: Flow chart of inclusion and exclusion criteria

Figure 2: Empirical cumulative distribution and frequency distribution of (Between) mean consultant volume and (Within) individual centred volume of hip arthroplasty in the previous 365-days.

Foot Note

Grey horizontal hashed lines indicate the 2.5th, 25th, 50th, 75th, and 97.5th centile of the distribution, vertical hashed lines indicate mean and centred consultant volume at 2.5th, 25th, 50th, 75th, and 97.5th centiles respectively.

Figure 3: Mean, interquartile range, and 95th centile range of consultant and centre volume of hip arthroplasty in the previous 365-days recorded in the NJR by individual consultant, and individual unit respectively.

Figure 4: ***Between-consultant*** *Marginal association of hip surgical volume in the preceding 365-days and hazard of revision arthroplasty adjusted with five different sets of confounding factors in a multi-level model.*

Foot Note.

Patient factors include sex, ASA grade, funder. Operation confounding factors include fixation, approach, position, anaesthetic, mechanical and chemical thromboprophylaxis, bearing, and year of operation. Centre confounding factors include hospital location and centre volume in the preceding 365-days. Surgeon confounding factors included; lead operating surgeon, listing of a surgeon within NJR prior to 2008, the proportion of NHS cases in the preceding year, and proportion hip arthroplasty procedures undertaken in the previous year.

For peer review only

Figure 5: ***Within-consultant*** *Marginal association of hip surgical volume in the preceding 365-days and hazard of revision arthroplasty adjusted with five different sets of confounding factors in a multi-level model.*

Foot Note.

Patient factors include sex, ASA grade, funder. Operation confounding factors include fixation, approach, position, anaesthetic, mechanical and chemical thromboprophylaxis, bearing, and year of operation. Centre confounding factors include hospital location and centre volume in the preceding 365-days. Surgeon confounding factors included; lead operating surgeon, listing of a surgeon within NJR prior to 2008, the proportion of NHS cases in the preceding year, and proportion hip arthroplasty procedures undertaken in the previous year.

For peer review only

For peer review only - http://bmjopen.bmj.com/site/about/guidelines.xhtml

1. Kreder HJ, Deyo RA, Koepsell T, Swiontkowski MF, Kreuter W. Relationship between the volume of total hip replacements performed by providers and the rates of postoperative complications in the state of Washington. J Bone Joint Surg Am. 1997;79(4):485-94. PubMed PMID: 9111392.
2. Kreder HJ, Williams JI, Jaglal S, Hu R, Axcell T, Stephen D. Are complication rates for elective primary total hip arthroplasty in Ontario related to surgeon and hospital volumes? A preliminary investigation. Can J Surg. 1998;41(6):431-7. PubMed PMID: 9854532.
3. Losina E, Barrett J, Mahomed NN, Baron JA, Katz JN. Early failures of total hip replacement: effect of surgeon volume. Arthritis Rheum. 2004;50(4):1338-43. doi: https://dx.doi.org/10.1002/art.20148. PubMed PMID: 15077318.
4. Manley M, Ong K, Lau E, Kurtz SM. Effect of volume on total hip arthroplasty revision rates in the United States Medicare population. J Bone Joint Surg Am. 2008;90(11):2446-51. doi: https://dx.doi.org/10.2106/JBJS.G.01300. PubMed PMID: 18978414.
5. Khatod M, Cafri G, Namba RS, Inacio MC, Paxton EW. Risk factors for total hip arthroplasty aseptic revision. J Arthroplasty. 2014;29(7):1412-7. doi: https://dx.doi.org/10.1016/j.arth.2014.01.023. PubMed PMID: 24582159.
6. Ravi B, Jenkinson R, Austin PC, Croxford R, Wasserstein D, Escott B, et al. Relation between surgeon volume and risk of complications after total hip arthroplasty: propensity score matched cohort study. Bmj. 2014;348:g3284. doi: https://dx.doi.org/10.1136/bmj.g3284. PubMed PMID: 24859902.
7. Katz JN, Losina E, Barrett J, Phillips CB, Mahomed NN, Lew RA, et al. Association between hospital and surgeon procedure volume and outcomes of total hip replacement in the United States medicare population. J Bone Joint Surg Am. 2001;83-A(11):1622-9. PubMed PMID: 11701783.
8. Lavernia CJ. Hemiarthroplasty in hip fracture care: effects of surgical volume on short-term outcome. J Arthroplasty. 1998;13(7):774-8. PubMed PMID: 9802663.
9. Lavernia CJ, Guzman JF. Relationship of surgical volume to short-term mortality, morbidity, and hospital charges in arthroplasty. J Arthroplasty. 1995;10(2):133-40. PubMed PMID: 7798093.
10. Katz JN, Phillips CB, Baron JA, Fossel AH, Mahomed NN, Barrett J, et al. Association of hospital and surgeon volume of total hip replacement with functional status and satisfaction three years following surgery. Arthritis Rheum. 2003;48(2):560-8. doi: https://dx.doi.org/10.1002/art.10754. PubMed PMID: 12571867.
11. Solomon DH, Losina E, Baron JA, Fossel AH, Guadagnoli E, Lingard EA, et al. Contribution of hospital characteristics to the volume-outcome relationship: dislocation and infection following total hip replacement surgery. Arthritis Rheum. 2002;46(9):2436-44. doi: https://dx.doi.org/10.1002/art.10478. PubMed PMID: 12355492.
12. Battaglia TC, Mulhall KJ, Brown TE, Saleh KJ. Increased surgical volume is associated with lower THA dislocation rates. Clin Orthop. 2006;447:28-33. doi: https://dx.doi.org/10.1097/01.blo.0000218743.99741.f0. PubMed PMID: 16672898.
13. Khatod M, Barber T, Paxton E, Namba R, Fithian D. An analysis of the risk of hip dislocation with a contemporary total joint registry. Clin Orthop. 2006;447:19-23. doi: https://dx.doi.org/10.1097/01.blo.0000218752.22613.78. PubMed PMID: 16741469.
14. Yasunaga H, Tsuchiya K, Matsuyama Y, Ohe K. High-volume surgeons in regard to reductions in operating time, blood loss, and postoperative complications for total hip

For peer review only - http://bmjopen.bmj.com/site/about/guidelines.xhtml

arthroplasty. J Orthop Sci. 2009;14(1):3-9. doi: https://dx.doi.org/10.1007/s00776-008-1289-4. PubMed PMID: 19214681.
15. Judge A, Chard J, Learmonth I, Dieppe P. The effects of surgical volumes and training centre status on outcomes following total joint replacement: analysis of the Hospital Episode Statistics for England. J Public Health (Oxf). 2006;28(2):116-24. doi: https://dx.doi.org/10.1093/pubmed/fdl003. PubMed PMID: 16597628.
16. Hodsman A, Ben-Shlomo Y, Roderick P, Tomson CRV. The 'Centre Effect' in Nephrology: What Do Differences between Nephrology Centres Tell Us about Clinical Performance in Patient Management? Nephron Clin Pract. 2011;119(1):C10-C7. doi: 10.1159/000321378. PubMed PMID: WOS:000291753000002.
17. Orsini N, Greenland S. A procedure to tabulate and plot results after flexible modeling of a quantitative covariate. Stata J. 2011;11(1):1-29. PubMed PMID: WOS:000292850600001.
18. Greenland S. Avoiding power loss associated with categorization and ordinal scores in dose-response and trend analysis. Epidemiology. 1995;6(4):450-4. Epub 1995/07/01. PubMed PMID: 7548361.
19. Smith AJ, Dieppe P, Howard PW, Blom AW, National Joint Registry for E, Wales. Failure rates of metal-on-metal hip resurfacings: analysis of data from the National Joint Registry for England and Wales. Lancet. 2012;380(9855):1759-66. Epub 2012/10/06. doi: 10.1016/S0140-6736(12)60989-1. PubMed PMID: 23036895.
20. Smith AJ, Dieppe P, Vernon K, Porter M, Blom AW, National Joint Registry of E, et al. Failure rates of stemmed metal-on-metal hip replacements: analysis of data from the National Joint Registry of England and Wales. Lancet. 2012;379(9822):1199-204. Epub 2012/03/16. doi: 10.1016/S0140-6736(12)60353-5. PubMed PMID: 22417410.
21. Neuhaus JM, McCulloch CE. Separating between- and within-cluster covariate effects by using conditional and partitioning methods. J Roy Stat Soc B. 2006;68:859-72. doi: DOI 10.1111/j.1467-9868.2006.00570.x. PubMed PMID: WOS:000241500400007.
22. Skrondal A, Rabe-Hesketh S. Generalized Latent Variable Modelling: Multilevel, Longitudinal , and Structural Equation Models. Boca Raton: Chapman & Hall/CRC; 2004.
23. Akaike H. Citation Classic - a New Look at the Statistical-Model Identification. Cc/Eng Tech Appl Sci. 1981;(51):22-. PubMed PMID: WOS:A1981MS54100001.
24. StataCorp. Stata Statistical Software: Release 14. College Station, TX: StataCorp LP; 2015.
25. White IR, Carlin JB. Bias and efficiency of multiple imputation compared with complete-case analysis for missing covariate values. Stat Med. 2010;29(28):2920-31. Epub 2010/09/16. doi: 10.1002/sim.3944. PubMed PMID: 20842622.
26. Schafer JL, Graham JW. Missing data: Our view of the state of the art. Psychol Methods. 2002;7(2):147-77. doi: 10.1037//1082-989x.7.2.147. PubMed PMID: WOS:000176079500001.
27. Bellemare MF, Masaki T, Pepinsky TB. Lagged Explanatory Variables and the Estimation of Causal Effect. J Polit. 2017;79(3):949-63. doi: 10.1086/690946. PubMed PMID: WOS:000403659000022.
28. Smith MC, Ben-Shlomo Y, Dieppe P, Beswick AD, Adebajo AO, Wilkinson JM, et al. Rates of hip and knee joint replacement amongst different ethnic groups in England: an analysis of National Joint Registry data. Osteoarthritis Cartilage. 2017;25(4):448-54. Epub 2017/02/06. doi: 10.1016/j.joca.2016.12.030. PubMed PMID: 28159557.

For peer review only

For peer review only - http://bmjopen.bmj.com/site/about/guidelines.xhtml

Figure1a. Inclusion Exclusion Criteria

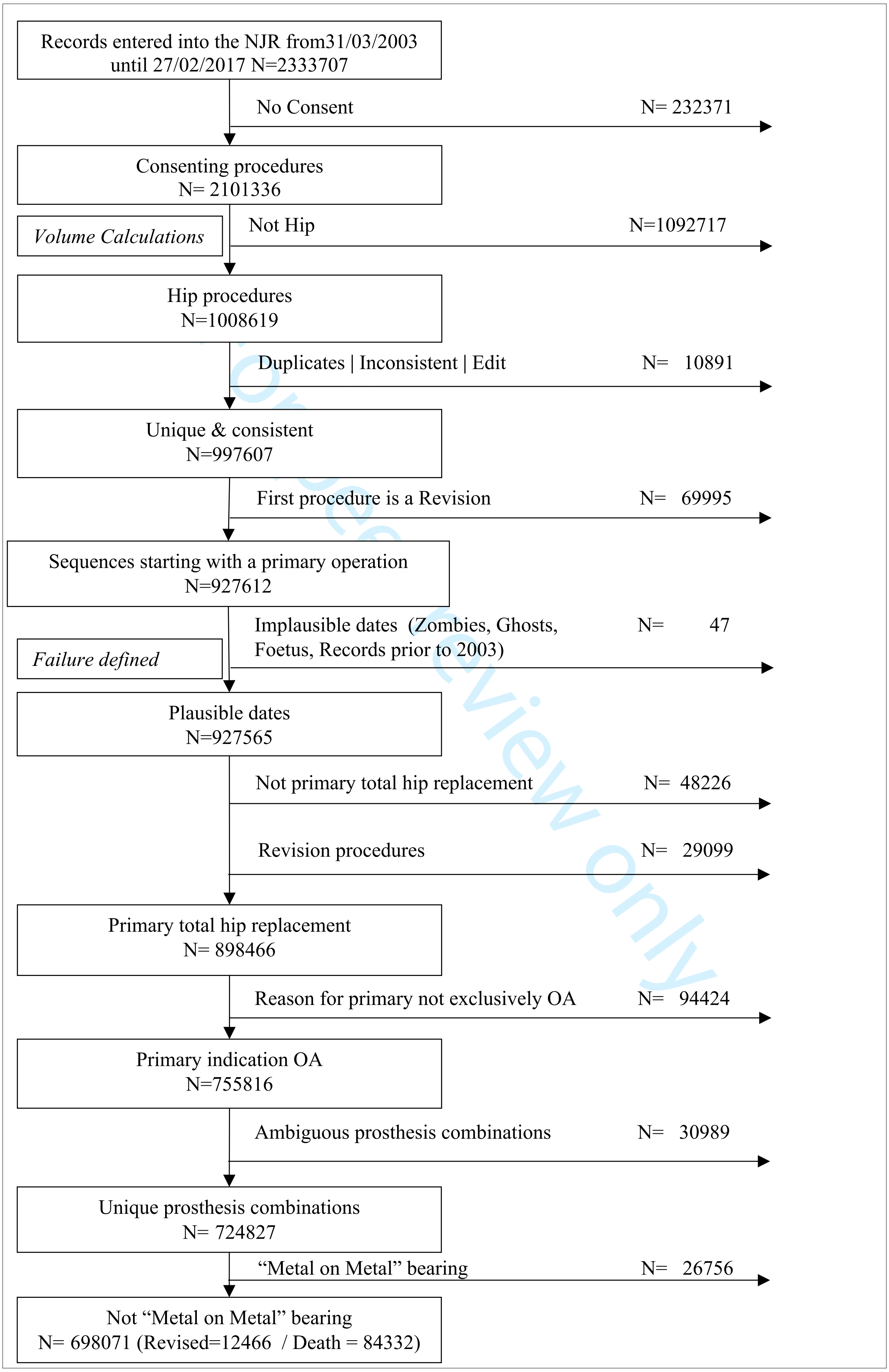

For peer review only

Figure1b. Missing Data

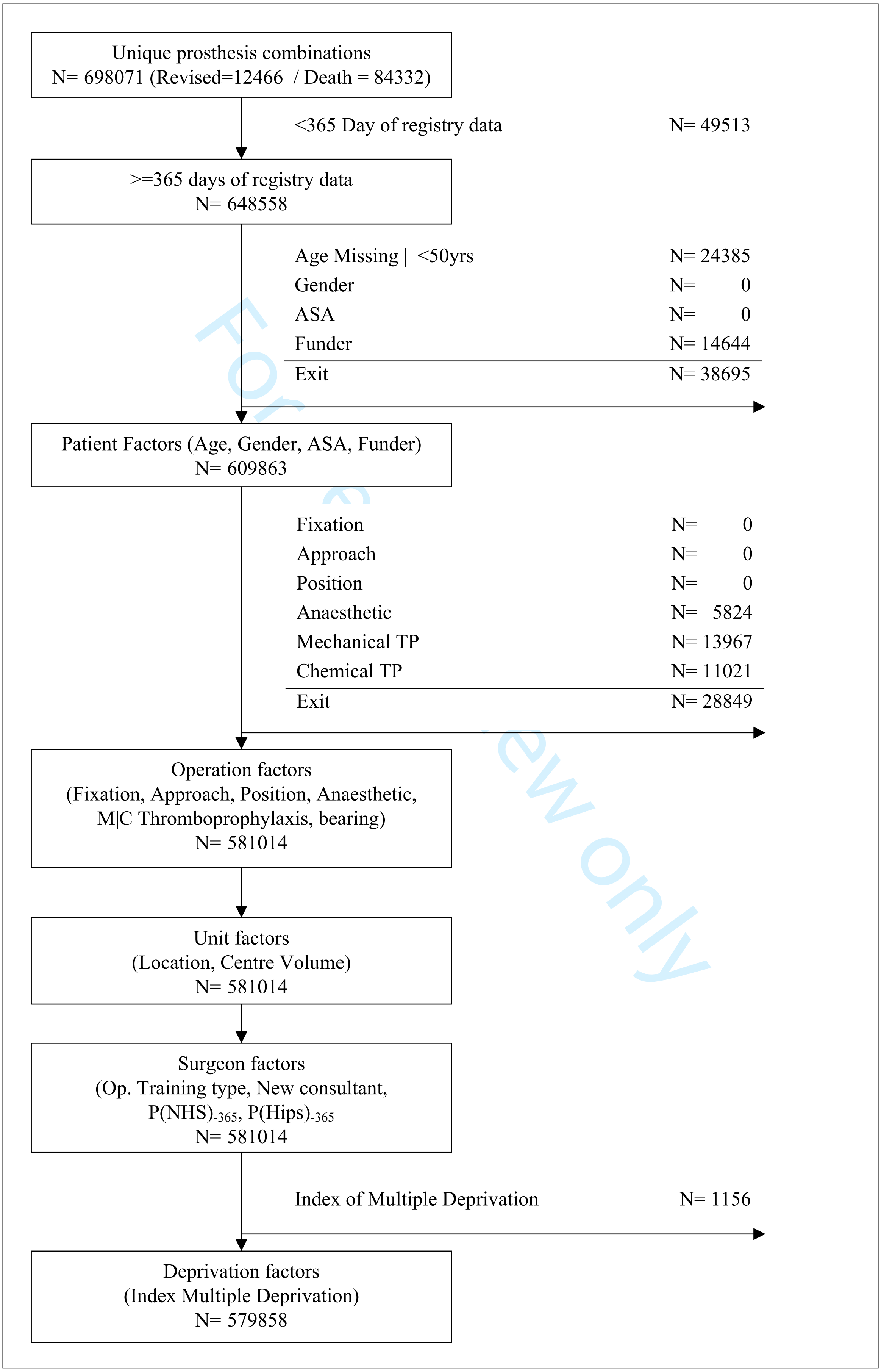

For peer review only

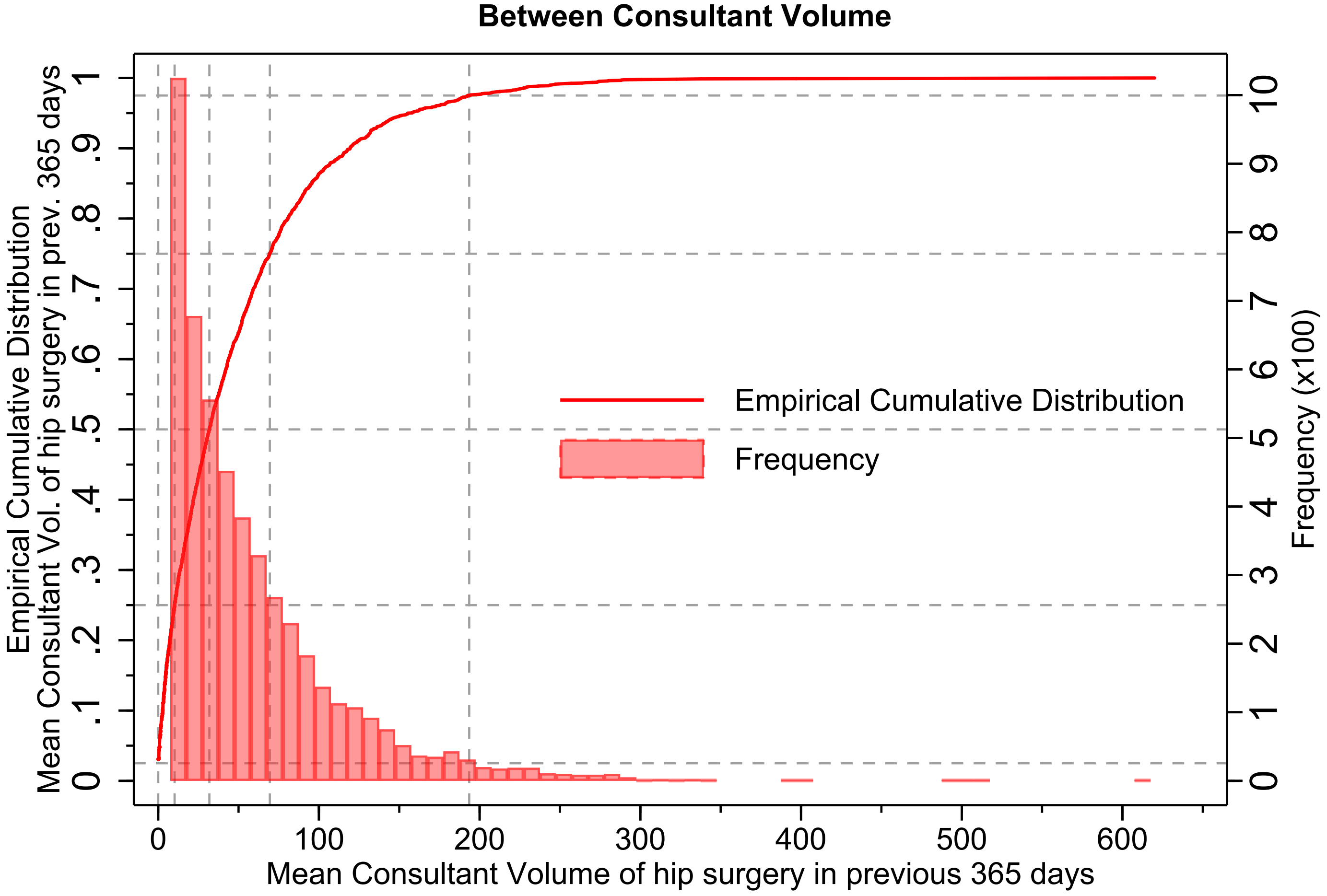
Between Consultant Volume
Empirical Cumulative Distribution
Frequency
Empirical Cumulative Distribution
Mean Consultant Vol. of hip surgery in prev. 365 days
Frequency (x100)
Mean Consultant Volume of hip surgery in previous 365 days
0
100
200
300
400
500
600
0
.1
.2
.3
.4
.5
.6
.7
.8
.9
1
0
1
2
3
4
5
6
7
8
9
10

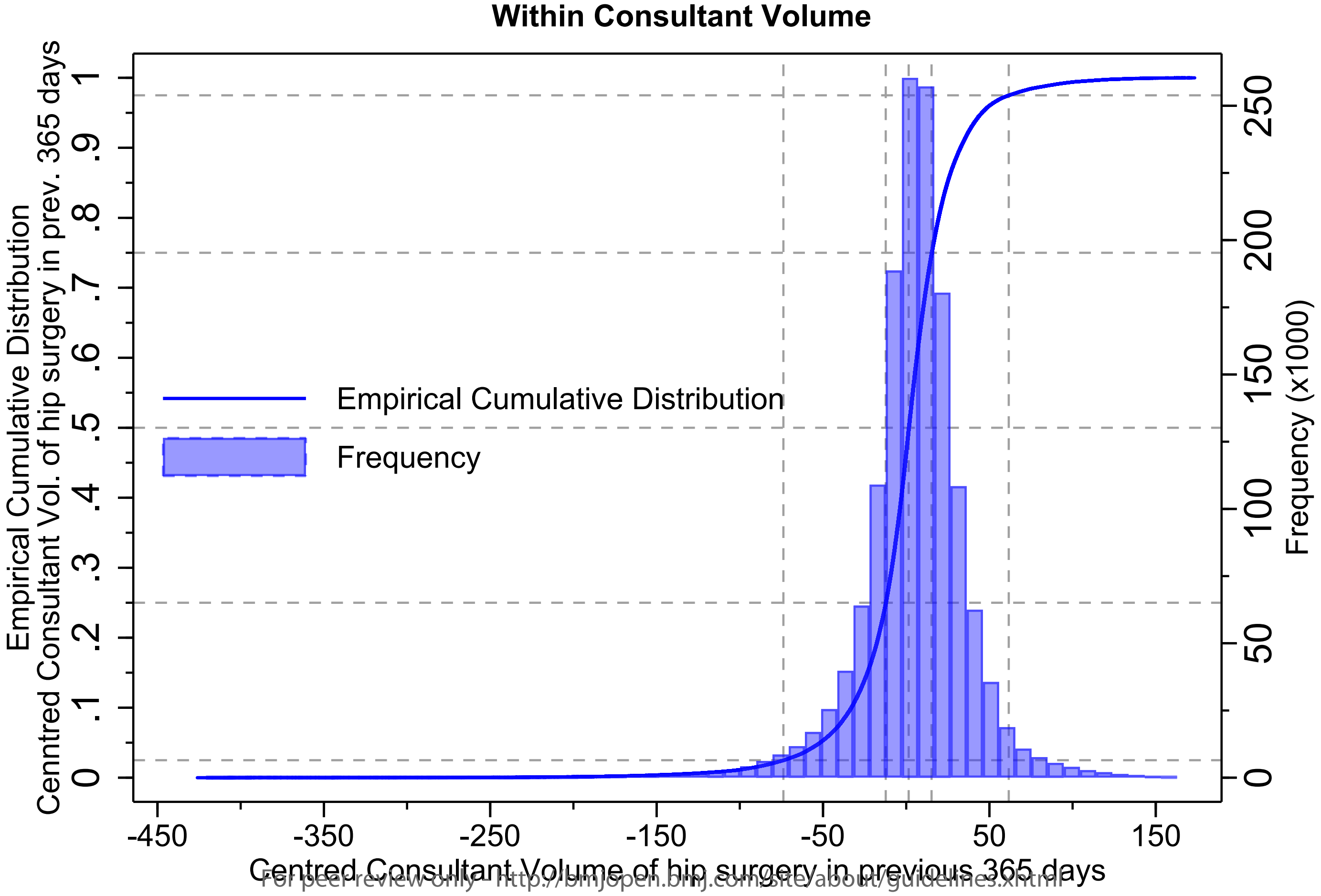
Within Consultant Volume
Empirical Cumulative Distribution
Frequency
Empirical Cumulative Distribution
Cenntred Consultant Vol. of hip surgery in prev. 365 days
Frequency (x1000)
Centred Consultant Volume of hip surgery in previous 365 days
-450
-350
-250
-150
-50
50
150
0
.1
.2
.3
.4
.5
.6
.7
.8
.9
1
0
50
100
150
200
250

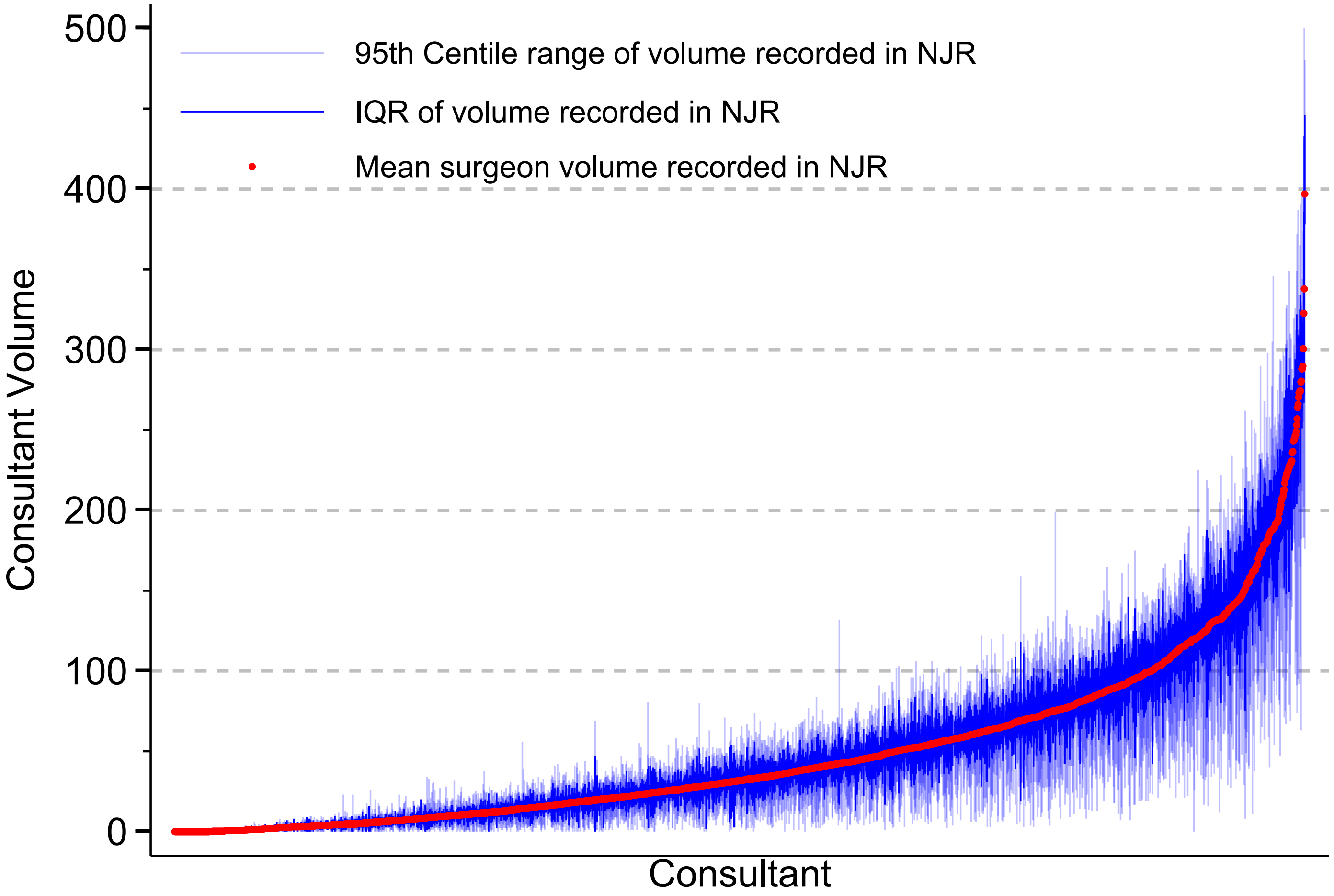
95th Centile range of volume recorded in NJR
IQR of volume recorded in NJR
Mean surgeon volume recorded in NJR
Consultant Volume
500
400
300
200
100
0
Consultant

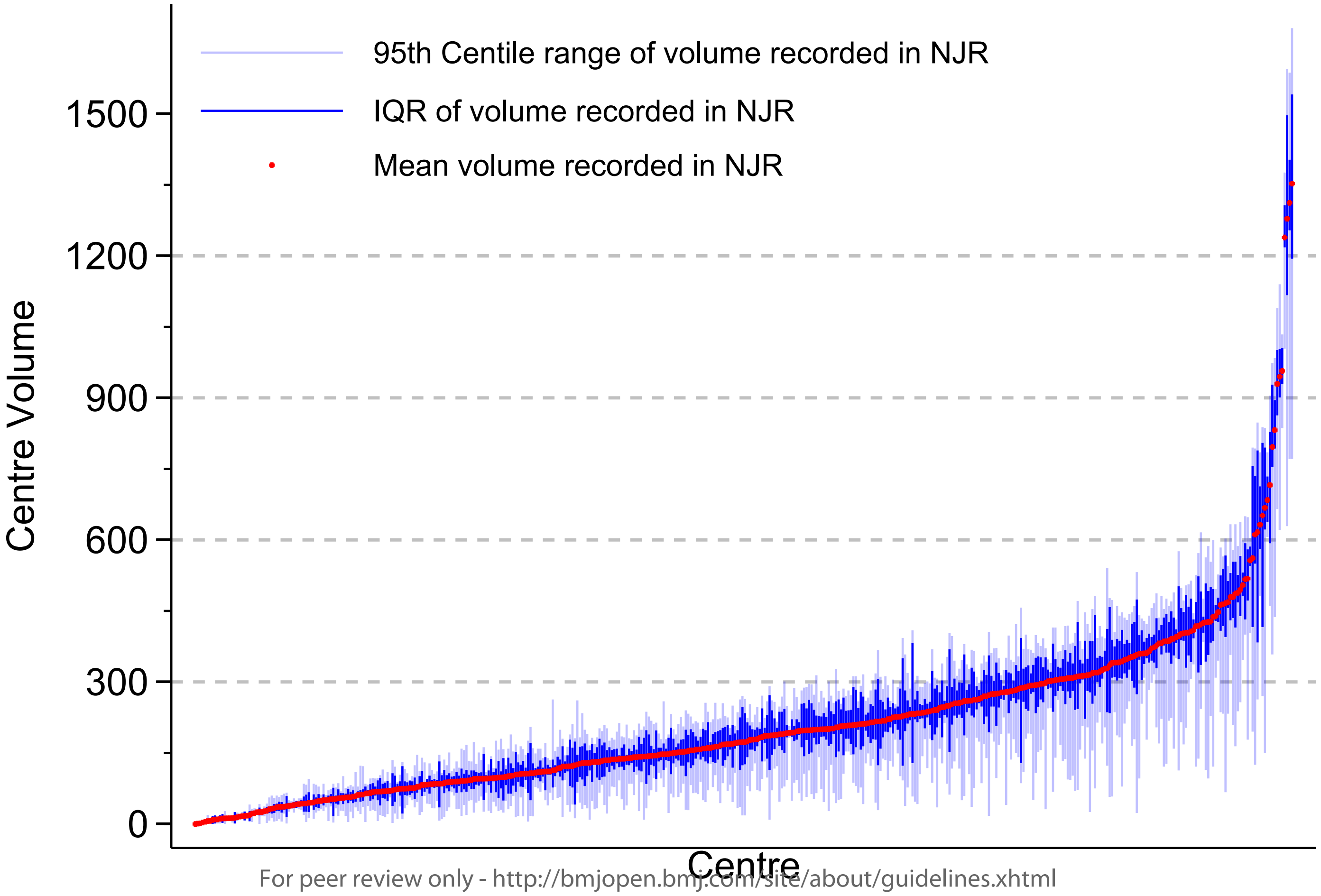
95th Centile range of volume recorded in NJR
IQR of volume recorded in NJR
Mean volume recorded in NJR
Centre Volume
1500
1200
900
600
300
0
Centre

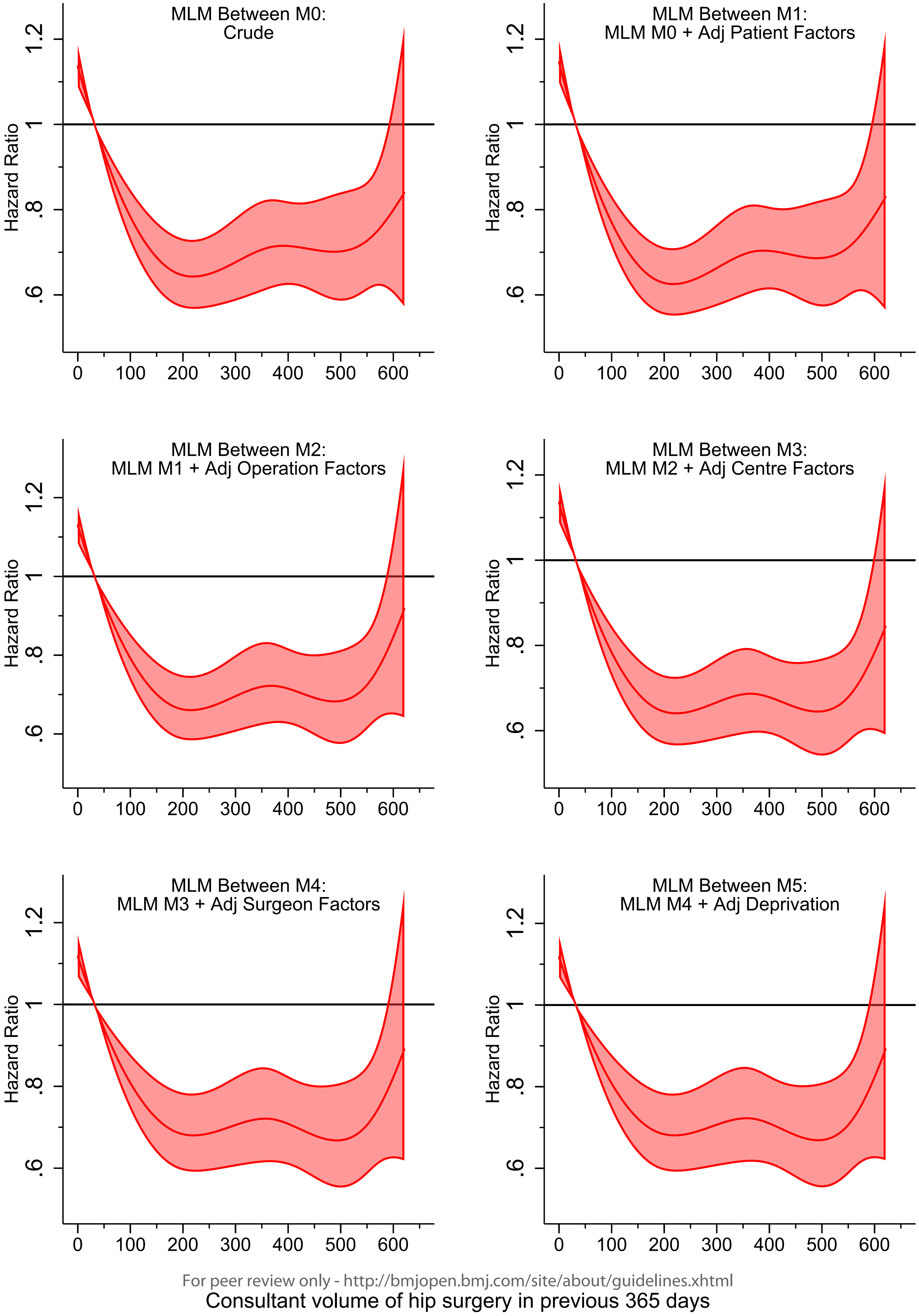

For peer review only - http://bmjopen.bmj.com/site/about/guidelines.xhtml

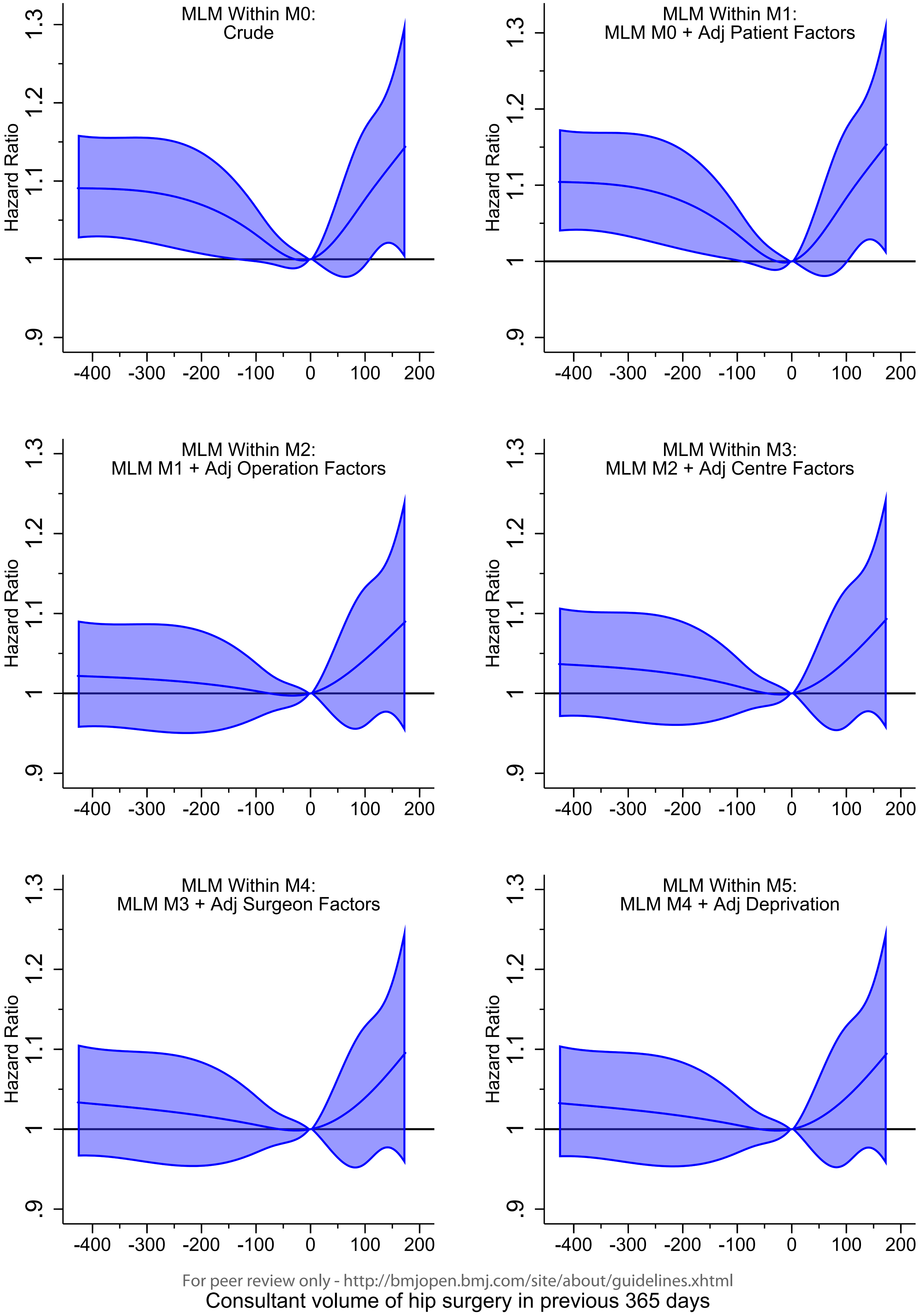
MLM Within M0:
Crude
MLM Within M1:
MLM M0 + Adj Patient Factors
MLM Within M2:
MLM M1 + Adj Operation Factors
MLM Within M3:
MLM M2 + Adj Centre Factors
MLM Within M4:
MLM M3 + Adj Surgeon Factors
MLM Within M5:
MLM M4 + Adj Deprivation
Hazard Ratio
.9
1
1.1
1.2
1.3
-400
-300
-200
-100
0
100
200
Consultant volume of hip surgery in previous 365 days

Supplementary Tables

Supplementary.Table 1: Coding and specification of confounding variables

Variable	Options	Baseline | Centred	Regression Specification
Pateint Age	Orthogonalised Restricted Cubic Splines Knots @ knots(50(10)90 102	70	Continuous
Sex	Male / Female	Male	Binary
American Society of Anesthesiologists (ASA) grade	I / II / III / IV+V	II	MV Binary
Operation funder	Public / Private	Public	Binary
Fixation	Cemented / Uncemented / Hybrid / Reverse Hybrid	Cemented	MV Binary
Approach	Posterior /Other	Posterior	Binary
Patient position during arthroplasty	Lateral / Supine	Lateral	Binary
Anaesthetic type	Spinal / GA / GA & Spinal / GA & Nerve Block/ Epidural / Nerve Block & Spinal / GA & Epidural / None / Epidural & Spinal / Nerve Block / GA & Nerve Block & Spinal / Other	Spinal	MV Binary
Mechanical thromboprophylaxis	TED / TED ICC / ICC / TED FP / FP / TED FP ICC / FP ICC / None	None	MV Binary
Chemical thromboprophylaxis	LMWH / Asprin/ DTI / LMWH Asprin / FXa / LMWH DTI / LMWH FXa / PS / LMWH Warf / Warf / LDH / LMWH PS /None /Other	None	MV Binary
Bearing	CoC / CoM /CoPe /MoC / MoPe / MoPu	MoPE	MV Binary

For peer review only

Year of arthroplasty	Orthogonalised Restricted Cubic Splines Knots @ 0(4.33)13	2004	Continuous
Setting of the treatment	NHS / Treatment Centre / Privates	NHS	MV Binary
Surgical centre volume	Orthogonalised Restricted Cubic Splines Knots @ 0(430)1720	97	Continuous
Training status of the primary surgeon	Consultant / Trainee	Consultant	Binary
Consultant was listed in the NJR prior to 2008	<2008 / >= 2008	<2008	Binary
proportion of THA undertaken in the NHS	Orthogonalised Restricted Cubic Splines Knots @ knots(0(0.25)1	0.7	Continuous
proportion of THA procedures	Orthogonalised Restricted Cubic Splines Knots @ knots(0(0.125)1	0.6	Continuous
Index of Multiple Deprivation (English)	Deciles	Decile #1	MV Binary
Welsh Index of Multiple Deprivation	Deciles	Decile #1	MV Binary

For peer review only

Supplementary.Table 2: Descriptive statistics of continuous variables used in the analysis.

Variable	Mean	(SD)	[25th	50th	75th]	Missing
(Traced) Age at operation date, rounded 2 DP	69.84	(10.2)	[63.56,	70.65,	77.16]	0
Consultant recorded hip volume in previous 365 days	118.36	(97.3)	[52.00,	95.00,	158.00]	0
SD consultant recorded hip volume in previous 365 days by consultant	28.13	(19.8)	[14.48,	23.48,	36.53]	114
Centre recorded hip volume in previous 365 days	370.18	(306.2)	[174.00,	284.00,	435.00]	0
SD centre recorded hip volume in previous 365 days by centre	81.84	(57.5)	[44.69,	65.90,	101.86]	2
Proportion of NHS hip procedures in previous 365 days	0.69	(0.3)	[0.54,	0.74,	0.94]	0
SD Proportion of NHS hip procedures in previous 365 day by consultant	0.10	(0.1)	[0.05,	0.10,	0.14]	114
Proportion of hip procedures in previous 365 days	0.60	(0.2)	[0.46,	0.58,	0.73]	0
SD Proportion of hip procedures in previous 365 days by consultant	0.06	(0.0)	[0.04,	0.06,	0.08]	114

For peer review only

Supplementary.Table 3: Descriptive statistics of categorical variable used in the analysis.

Variable	Factor	N	(%)
Gender	Female	397107	(61.1)
	Male	252852	(38.9)
	Missing	0	(0.0)
ASA Grade	I	94042	(14.5)
	II	457780	(70.4)
	III	95293	(14.7)
	IV +V	2844	(0.4)
	Missing	0	(0.0)
Procedure funded by:	NHS	546727	(84.1)
	Private	88588	(13.6)
	Missing	14644	(2.3)
Method of fixation	Cemented	236554	(36.4)
	Uncemented	260857	(40.1)
	Hybrid	133489	(20.5)
	Reverse Hybrid	19059	(2.9)
	Missing	0	(0.0)
Surgical Approach	Posterior	376818	(58.0)
	Other	273141	(42.0)
	Missing	0	(0.0)
Patient Position	Lateral	595964	(91.7)
	Supine	53995	(8.3)
	Missing	0	(0.0)
Anaesthetic combination	Sp	329954	(50.8)
	GA	141132	(21.7)
	GA Sp	79706	(12.3)
	GA NB	29658	(4.6)
	Epi	18759	(2.9)
	NB Sp	16450	(2.5)
	GA Epi	13235	(2.0)
	None	103	(0.0)

For peer review only

	Epi Sp	5449	(0.8)
	NB	3912	(0.6)
	GA NB Sp	2353	(0.4)
	Other	600	(0.1)
	Missing	8648	(1.3)
Mechanical thromboprophylaxis combination	TED	198597	(30.6)
	TED ICC	128881	(19.8)
	ICC	101050	(15.5)
	TED FP	77150	(11.9)
	FP	58467	(9.0)
	None	33814	(5.2)
	TED FP ICC	26230	(4.0)
	FP ICC	8994	(1.4)
	Missing	16776	(2.6)
Chemical thromboprophylaxis combination	LMWH	397523	(61.2)
	None	57057	(8.8)
	Asprin	44497	(6.8)
	DTI	36394	(5.6)
	LMWH Asprin	29615	(4.6)
	FXa	31768	(4.9)
	LMWH DTI	14628	(2.3)
	LMWH FXa	7391	(1.1)
	PS	4875	(0.8)
	LMWH Warf	3712	(0.6)
	Warf	3264	(0.5)
	LDH	1299	(0.2)
	LMWH PS	1781	(0.3)
	Other	1364	(0.2)
	Missing	14791	(2.3)
Bearing Surface [Head on Acetab.]	CoC	108539	(16.7)
	CoM	1916	(0.3)
	CoPe	119379	(18.4)
	MoC	115	(0.0)
	MoPe	419844	(64.6)

For peer review only

	MoPu	166	(0.0)
	Missing	0	(0.0)
Hospital provider category	NHS	420897	(64.8)
	TreatmentCentre	27233	(4.2)
	Private	201829	(31.1)
	Missing	0	(0.0)
Year of operation	04	3783	(0.6)
	05	22638	(3.5)
	06	29726	(4.6)
	07	38141	(5.9)
	08	42083	(6.5)
	09	46407	(7.1)
	10	52725	(8.1)
	11	57465	(8.8)
	12	63544	(9.8)
	13	66043	(10.2)
	14	71634	(11.0)
	15	71316	(11.0)
	16	72761	(11.2)
	17	11693	(1.8)
	Missing	0	(0.0)
Operation training type	Con.(1)/Con.(1)	520620	(80.1)
	Con.(1)/Con.(1)(?)	18202	(2.8)
	Con.(1)/Con.(2)	15041	(2.3)
	Con.(1)/Jun.(1)/Con.	37414	(5.8)
	Con.(1)/Jun.(1)/Jun.(2)	58682	(9.0)
	Missing	0	(0.0)
Consultant first recorded procedure post 2008 + 365days	Cons. Pre 2009	547791	(84.3)
	Cons. Post 2009	102168	(15.7)
	Missing	0	(0.0)
Index of Multiple Deprivation Deciles (Wales)	1	1945	(0.3)
	2	2460	(0.4)
	3	2854	(0.4)
	4	3183	(0.5)

For peer review only

	5	3668	(0.6)
	6	4351	(0.7)
	7	4596	(0.7)
	8	4498	(0.7)
	9	4294	(0.7)
	10	3630	(0.6)
	Missing	614480	(94.5)
Index of Multiple Deprivation Deciles (England)	1	32697	(5.0)
	2	37030	(5.7)
	3	43721	(6.7)
	4	54200	(8.3)
	5	63862	(9.8)
	6	71498	(11.0)
	7	74777	(11.5)
	8	76272	(11.7)
	9	78245	(12.0)
	10	80544	(12.4)
	Missing	37113	(5.7)

For peer review only

Supplementary.Table 4: Consultant volume summary statistics by potential confounding factors.

Variable	Category	N	Mean	(SD)	[25th	50th	75th]
Age at operation	50 to 55yrs	28129	129.82	(97.4)	[64.00,	108.00,	170.00]
	55 to 60yrs	48289	124.41	(95.9)	[59.00,	103.00,	164.00]
	60 to 65yrs	78757	121.97	(97.5)	[55.00,	99.00,	163.00]
	65 to 70yrs	109204	120.88	(98.6)	[53.00,	97.00,	161.00]
	70 to 75yrs	116901	118.50	(97.9)	[51.00,	95.00,	158.00]
	75 to 80yrs	104115	117.07	(98.9)	[50.00,	93.00,	156.00]
	80 to 85yrs	64385	117.64	(99.4)	[50.00,	93.00,	157.00]
	85 to 90yrs	25244	119.65	(101.7)	[51.00,	94.00,	159.00]
	>90yrs	4834	123.76	(105.2)	[53.00,	97.00,	165.00]
Gender	Female	355154	120.68	(99.3)	[53.00,	97.00,	161.00]
	Male	224704	119.45	(96.9)	[53.00,	96.00,	159.00]
ASA Grade	I	75489	129.62	(105.2)	[56.00,	103.00,	176.00]
	II	414552	120.68	(98.9)	[53.00,	97.00,	160.00]
	III	87278	110.27	(89.1)	[49.00,	88.00,	148.00]
	IV +V	2539	103.76	(80.5)	[46.00,	83.00,	140.00]
Procedure funded by:	NHS	500193	116.38	(94.5)	[52.00,	94.00,	155.00]
	Private	79665	144.25	(117.2)	[63.00,	116.00,	196.00]
Method of fixation	Cemented	209831	100.24	(73.9)	[43.00,	82.00,	140.00]
	Uncemented	231302	123.73	(100.1)	[56.00,	96.00,	160.00]
	Hybrid	121293	149.75	(124.2)	[71.00,	123.00,	192.00]
	Reverse Hybrid	17432	108.24	(68.9)	[57.00,	93.50,	147.00]
Hospital provider category	NHS	371858	113.06	(93.7)	[50.00,	90.00,	151.00]
	Treatment Centre	25296	103.59	(56.0)	[60.00,	100.00,	134.00]
	Private	182704	137.05	(109.5)	[61.00,	109.00,	184.00]
P(NHS) in previous 365-days	<0.25	59325	115.76	(109.8)	[42.00,	85.00,	145.00]
	>=0.25 & <0.5	70297	149.07	(118.0)	[60.00,	115.00,	212.00]
	>=0.5 & <0.75	170720	137.44	(100.4)	[72.00,	117.00,	178.00]
	>=0.75 & <1	278820	103.62	(84.7)	[45.00,	83.00,	140.00]
P(Hip Arthroplasty) in previous 365-days	<0.25	21128	25.62	(21.4)	[10.00,	21.00,	33.00]

For peer review only

	>=0.25 & <0.5	176666	66.41	(40.2)	[36.00,	59.00,	89.00]
	>=0.5 & <0.75	255607	119.99	(67.8)	[69.00,	109.00,	159.00]
	>=0.75 & <1	126204	212.03	(137.2)	[123.00,	188.00,	265.00]
Index of Multiple Deprivation Deciles (Wales)	1	1657	98.69	(78.0)	[39.00,	72.00,	148.00]
	2	2153	98.57	(74.4)	[40.00,	76.00,	142.00]
	3	2522	91.51	(67.8)	[40.00,	71.00,	129.00]
	4	2809	88.34	(68.7)	[38.00,	69.00,	121.00]
	5	3343	91.71	(72.2)	[36.00,	70.00,	130.00]
	6	3961	90.30	(67.8)	[38.00,	70.00,	130.00]
	7	4249	97.31	(70.0)	[43.00,	77.00,	138.00]
	8	4135	98.71	(70.1)	[44.00,	80.00,	139.00]
	9	3947	115.23	(84.7)	[49.00,	92.00,	174.00]
	10	3265	128.15	(85.1)	[56.00,	108.00,	198.00]
Index of Multiple Deprivation Deciles (England)	1	28022	98.15	(75.1)	[45.00,	81.00,	131.00]
	2	32304	101.47	(81.3)	[46.00,	83.00,	135.00]
	3	38370	106.67	(89.1)	[47.00,	85.00,	140.00]
	4	47978	111.39	(88.7)	[51.00,	90.00,	147.00]
	5	57129	118.02	(97.2)	[53.00,	94.00,	155.00]
	6	64237	124.51	(101.9)	[56.00,	100.00,	164.00]
	7	67491	125.95	(103.4)	[57.00,	101.00,	166.00]
	8	68831	127.19	(103.2)	[57.00,	103.00,	170.00]
	9	70537	130.69	(105.0)	[59.00,	106.00,	174.00]
	10	72918	134.51	(108.8)	[60.00,	111.00,	179.00]
Surgical Approach	Posterior	342849	144.30	(108.1)	[71.00,	121.00,	189.00]
	Other	237009	85.35	(68.7)	[37.00,	69.00,	115.00]
Patient Position	Lateral	535280	122.45	(99.0)	[55.00,	99.00,	164.00]
	Supine	44578	93.27	(87.1)	[40.00,	70.00,	112.00]
Anaesthetic combination	Sp	305186	116.39	(88.7)	[56.00,	98.00,	154.00]
	GA	125359	109.36	(87.5)	[46.00,	86.00,	151.00]
	GA Sp	71916	162.93	(131.8)	[68.00,	128.00,	221.00]
	GA NB	25213	105.09	(83.1)	[44.00,	84.00,	144.00]
	Epi	16568	95.02	(84.9)	[38.00,	71.00,	132.00]
	NB Sp	15451	132.51	(151.0)	[53.00,	86.00,	137.00]

For peer review only

	GA Epi	9820	130.50	(98.4)	[52.00,	102.00,	202.00]
	None	59	115.80	(78.9)	[49.00,	103.00,	167.00]
	Epi Sp	4041	104.14	(79.2)	[43.00,	83.00,	155.00]
	NB	3524	101.01	(83.8)	[48.00,	79.00,	133.00]
	GA NB Sp	2188	163.59	(115.9)	[78.00,	149.00,	230.00]
	Other	533	111.07	(112.9)	[44.00,	85.00,	143.00]
Mechanical thromboprophylaxis combination	TED	183510	115.60	(104.1)	[49.00,	92.00,	149.00]
	TED ICC	119415	118.20	(101.3)	[51.00,	90.00,	151.00]
	ICC	92340	109.16	(77.2)	[52.00,	91.00,	151.00]
	TED FP	67618	146.91	(118.8)	[67.00,	120.00,	187.00]
	FP	53375	132.33	(86.1)	[62.00,	122.00,	190.00]
	None	31757	117.05	(79.4)	[53.00,	100.00,	170.00]
	TED FP ICC	23445	115.61	(84.4)	[58.00,	91.00,	151.00]
	FP ICC	8398	103.40	(81.1)	[48.00,	83.00,	144.00]
Chemical thromboprophylaxis combination	LMWH	362081	113.44	(94.4)	[50.00,	91.00,	150.00]
	None	53791	129.35	(106.3)	[54.00,	99.00,	176.00]
	Asprin	38635	125.01	(88.2)	[56.00,	110.00,	170.00]
	DTI	34669	117.68	(78.4)	[59.00,	100.00,	163.00]
	LMWH Asprin	26428	146.37	(111.5)	[64.00,	125.00,	207.00]
	FXa	30026	153.06	(120.9)	[73.00,	122.00,	194.00]
	LMWH DTI	13840	138.70	(127.3)	[56.00,	99.00,	186.00]
	LMWH FXa	7019	128.35	(122.9)	[58.00,	99.00,	159.00]
	PS	4553	102.17	(64.2)	[50.00,	91.00,	144.00]
	LMWH Warf	3028	121.75	(98.5)	[55.00,	103.00,	164.00]
	Warf	2616	115.38	(73.6)	[57.00,	117.00,	152.00]
	LDH	677	91.82	(86.9)	[28.00,	47.00,	168.00]
	LMWH PS	1360	104.65	(77.3)	[49.00,	86.00,	160.00]
	Other	1135	120.09	(117.3)	[56.00,	88.00,	154.00]
Bearing Surface [Head on Acetab.]	CoC	89379	125.46	(90.5)	[61.00,	104.00,	166.00]
	CoM	1728	186.67	(141.7)	[69.00,	121.00,	286.50]
	CoPe	106359	138.64	(125.1)	[60.00,	104.00,	178.00]
	MoC	100	103.72	(88.3)	[41.50,	68.50,	140.50]
	MoPe	382139	113.55	(90.3)	[49.00,	92.00,	154.00]
	MoPu	153	123.27	(46.2)	[92.00,	130.00,	160.00]

For peer review only

Year of operation	04	1902	57.84	(47.1)	[24.00,	45.00,	77.00]
	05	12392	73.87	(60.3)	[29.00,	56.00,	101.00]
	06	16901	91.97	(81.6)	[33.00,	69.00,	124.00]
	07	22287	97.09	(83.3)	[36.00,	75.00,	133.00]
	08	37678	111.83	(98.2)	[44.00,	86.00,	149.00]
	09	44020	115.91	(102.8)	[45.00,	89.00,	151.00]
	10	50416	114.78	(95.7)	[48.00,	89.00,	155.00]
	11	54957	116.60	(98.2)	[50.00,	91.00,	154.00]
	12	60581	122.55	(99.0)	[55.00,	99.00,	163.00]
	13	62950	124.14	(94.3)	[58.00,	100.00,	169.00]
	14	68357	127.82	(94.1)	[63.00,	106.00,	171.00]
	15	68189	133.04	(103.8)	[64.00,	111.00,	172.00]
	16	69434	130.91	(106.2)	[65.00,	107.00,	169.00]
	17	9794	133.73	(106.7)	[67.00,	108.00,	175.00]
Center Volume in previous 365 days	1x day	252914	99.94	(90.5)	[40.00,	75.00,	130.00]
	2x day	223425	116.00	(76.7)	[59.00,	101.00,	158.00]
	3x day	40127	142.01	(86.8)	[80.00,	127.00,	191.00]
	4x day	33584	226.83	(167.5)	[115.00,	183.00,	289.00]
	5x day	14127	198.14	(143.4)	[99.00,	172.00,	250.00]
	6x day	12630	151.66	(73.8)	[99.00,	133.00,	206.00]
	7x day	3051	156.37	(64.7)	[110.00,	142.00,	220.00]
Operation training type	Con.(1)/Con.(1)	463705	117.46	(91.7)	[53.00,	95.00,	157.00]
	Con.(1)/Con.(1)(?)	16246	105.20	(115.9)	[40.00,	67.00,	133.00]
	Con.(1)/Con.(2)	13637	141.29	(134.4)	[49.00,	101.00,	197.00]
	Con.(1)/Jun.(1)/Con.	34006	110.47	(72.3)	[56.00,	101.00,	153.00]
	Con.(1)/Jun.(1)/Jun.(2)	52264	150.02	(139.3)	[58.00,	119.00,	191.00]
Consultant first recorded procedure post 2008 + 365days	Cons. Pre 2009	482572	126.19	(104.1)	[54.00,	101.00,	170.00]
	Cons. Post 2009	97286	90.53	(53.7)	[50.00,	81.00,	122.00]

For peer review only

For peer review only

STROBE Statement—Checklist of items that should be included in reports of ***cohort studies***

	Item No	Recommendation	Page
**Title and abstract**	1	(*a*) Indicate the study's design with a commonly used term in the title or the abstract	1
		(*b*) Provide in the abstract an informative and balanced summary of what was done and what was found	2
**Introduction**			
Background/rationale	2	Explain the scientific background and rationale for the investigation being reported	4
Objectives	3	State specific objectives, including any prespecified hypotheses	5
**Methods**			
Study design	4	Present key elements of study design early in the paper	6
Setting	5	Describe the setting, locations, and relevant dates, including periods of 6recruitment, exposure, follow-up, and data collection	6
Participants	6	(*a*) Give the eligibility criteria, and the sources and methods of selection of participants. Describe methods of follow-up	6
		(*b*) For matched studies, give matching criteria and number of exposed and unexposed	
Variables	7	Clearly define all outcomes, exposures, predictors, potential confounders, and effect modifiers. Give diagnostic criteria, if applicable	7
Data sources/ measurement	8*	For each variable of interest, give sources of data and details of methods of assessment (measurement). Describe comparability of assessment methods if there is more than one group	*7/8*
Bias	9	Describe any efforts to address potential sources of bias	7/8
Study size	10	Explain how the study size was arrived at	8
Quantitative variables	11	Explain how quantitative variables were handled in the analyses. If applicable, describe which groupings were chosen and why	8 sup.t1
Statistical methods	12	(*a*) Describe all statistical methods, including those used to control for confounding	8
		(*b*) Describe any methods used to examine subgroups and interactions	n/a
		(*c*) Explain how missing data were addressed	9
		(*d*) If applicable, explain how loss to follow-up was addressed	7
		(*e*) Describe any sensitivity analyses	n/a
**Results**			
Participants	13*	(a) Report numbers of individuals at each stage of study—eg numbers potentially eligible, examined for eligibility, confirmed eligible, included in the study, completing follow-up, and analysed	Fig 1
		(b) Give reasons for non-participation at each stage	Fig 1
		(c) Consider use of a flow diagram	Fig 1
Descriptive data	14*	(a) Give characteristics of study participants (eg demographic, clinical, social) and information on exposures and potential confounders	ST2/3
		(b) Indicate number of participants with missing data for each variable of interest	Fig 1
		(c) Summarise follow-up time (eg, average and total amount)	6
Outcome data	15*	Report numbers of outcome events or summary measures over time	10
Main results	16	(*a*) Give unadjusted estimates and, if applicable, confounder-adjusted estimates and their precision (eg, 95% confidence interval). Make clear	Fig 4/5

For peer review only

For peer review only - http://bmjopen.bmj.com/site/about/guidelines.xhtml

		which confounders were adjusted for and why they were included	
		(*b*) Report category boundaries when continuous variables were categorized	n/a
		(*c*) If relevant, consider translating estimates of relative risk into absolute risk for a meaningful time period	n/a
Other analyses	17	Report other analyses done—eg analyses of subgroups and interactions, and sensitivity analyses	n/a
**Discussion**			
Key results	18	Summarise key results with reference to study objectives	12
Limitations	19	Discuss limitations of the study, taking into account sources of potential bias or imprecision. Discuss both direction and magnitude of any potential bias	13
Interpretation	20	Give a cautious overall interpretation of results considering objectives, limitations, multiplicity of analyses, results from similar studies, and other relevant evidence	14
Generalisability	21	Discuss the generalisability (external validity) of the study results	15
**Other information**			
Funding	22	Give the source of funding and the role of the funders for the present study and, if applicable, for the original study on which the present article is based	16

*Give information separately for exposed and unexposed groups.

Note: An Explanation and Elaboration article discusses each checklist item and gives methodological background and published examples of transparent reporting. The STROBE checklist is best used in conjunction with this article (freely available on the Web sites of PLoS Medicine at http://www.plosmedicine.org/, Annals of Internal Medicine at http://www.annals.org/, and Epidemiology at http://www.epidem.com/). Information on the STROBE Initiative is available at http://www.strobe-statement.org.

For peer review only

BMJ Open

The association between surgical volume and failure of primary total hip replacement in England and Wales: Findings from a prospective national joint replacement register

Journal:	*BMJ Open*
Manuscript ID	bmjopen-2019-033045.R1
Article Type:	Original research
Date Submitted by the Author:	09-Jan-2020
Complete List of Authors:	Sayers, Adrian; University of Bristol, Muscloskeletal Research Unit<br>Steele, Fiona; LSE, Department of Statistics<br>Whitehouse, Michael; University of Bristol, Musculoskeletal Research Unit<br>Price, Andrew; University of Oxford, Nuffield Department of Orthopaedics, Rheumatology and Musculoskeletal Sciences<br>Ben-Shlomo, Yoav; University of Bristol,<br>Blom, AW; University of Bristol,
**Primary Subject Heading**:	Epidemiology
Secondary Subject Heading:	Surgery, Epidemiology, Public health, Research methods
Keywords:	Total Hip Arthroplasty, Hip < ORTHOPAEDIC & TRAUMA SURGERY, volume, Revision surgery, Within Surgeon Effects, Between Surgeon effects

SCHOLARONE™
Manuscripts

I, the Submitting Author has the right to grant and does grant on behalf of all authors of the Work (as defined in the below author licence), an exclusive licence and/or a non-exclusive licence for contributions from authors who are: i) UK Crown employees; ii) where BMJ has agreed a CC-BY licence shall apply, and/or iii) in accordance with the terms applicable for US Federal Government officers or employees acting as part of their official duties; on a worldwide, perpetual, irrevocable, royalty-free basis to BMJ Publishing Group Ltd ("BMJ") its licensees and where the relevant Journal is co-owned by BMJ to the co-owners of the Journal, to publish the Work in this journal and any other BMJ products and to exploit all rights, as set out in our licence.

The Submitting Author accepts and understands that any supply made under these terms is made by BMJ to the Submitting Author unless you are acting as an employee on behalf of your employer or a postgraduate student of an affiliated institution which is paying any applicable article publishing charge ("APC") for Open Access articles. Where the Submitting Author wishes to make the Work available on an Open Access basis (and intends to pay the relevant APC), the terms of reuse of such Open Access shall be governed by a Creative Commons licence – details of these licences and which Creative Commons licence will apply to this Work are set out in our licence referred to above.

Other than as permitted in any relevant BMJ Author's Self Archiving Policies, I confirm this Work has not been accepted for publication elsewhere, is not being considered for publication elsewhere and does not duplicate material already published. I confirm all authors consent to publication of this Work and authorise the granting of this licence.

For peer review only

Full Title: The association between surgical volume and failure of primary total hip replacement in England and Wales: Findings from a prospective national joint replacement register

Short Title: Surgical volume and failure of primary total hip replacement

Adrian Sayers[1,2], Fiona Steele[3], Michael R Whitehouse[1,5], Andrew Price[4], Yoav Ben-Shlomo[2], Ashley W Blom[1,5]

1. Musculoskeletal Research Unit, Bristol Medical School, 1st Floor Learning & Research Building, Southmead Hospital, Bristol, BS10 5NB, United Kingdom
2. Population Health Sciences, Bristol Medical School, Canynge Hall, 39 Whatley Road Bristol, BS8 2PS, United Kingdom
3. Department of Statistics, London School of Economics and Political Science, London, UK
4. NIHR Biomedical Research Unit, University of Oxford, Oxford, UK
5. National Institute for Health Research Bristol Biomedical Research Centre, University Hospitals Bristol NHS Foundation Trust and University of Bristol

Corresponding Author

Adrian Sayers

Musculoskeletal Research Unit, Bristol Medical School, 1st Floor Learning & Research Building, Southmead Hospital, Bristol, BS10 5NB, United Kingdom

Email: adrian.sayers@bristol.ac.uk Tel: 0117 4147880

Word Count:2826

For peer review only - http://bmjopen.bmj.com/site/about/guidelines.xhtml

For peer review only

Abstract

Objective

Investigate the association of volume of total hip arthroplasty (THA) between consultants and within the same consultant in the previous year and the hazard of revision using multi-level survival models.

Design

Prospective cohort study using data from a national joint replacement register.

Setting

Elective total hip arthroplasty (THA) across all private and public centres in England and Wales between April 2003 and February 2017.

Participants

Patients age 50 years or more undergoing THA for osteoarthritis.

Intervention

The volume of THA conducted in the preceding 365-days to the index procedure.

Main Outcome and measure

Revision surgery (excision, addition or replacement) of a primary THA.

Results

Of the 579,858 patients undergoing primary THA (mean baseline age 69.8 years [SD 10.2]), 61.1% were female. Multi-level survival found differing results for between and within-consultant effects. There was a strong volume-revision association between consultants, with a near linear 43.3% (95% CI 29.1%, 57.4%) reduction of the risk of revision comparing consultants with volumes between 1 and 200 procedures annually. Changes in individual

For peer review only

surgeons (within-consultant) case volume showed no evidence of an association with revision.

Conclusion

Separation of between- and within-consultant effects of surgical volume reveals how volume contributes to the risk of revision after THA. The lack of association within-consultants suggests that individual changes to consultant volume alone will have little effect on outcomes following THA.

These novel findings provide strong evidence supporting the practice of specialisation of hip arthroplasty. It does not support the practice of low volume consultants increasing their personal volume as it is unlikely their results would improve if this is the only change. Limiting the exposure of patients to consultants with low volumes of THA and greater utilization of centres with higher volume surgeons with better outcomes may be beneficial to patients.

Strengths and limitations of this study

- This is the largest study in the world to explore the association between surgical volume and outcomes in total hip arthroplasty
- We uniquely calculate a time-varying exposure of surgical volume.
- We differentiate between- and within- consultant effects using a multi-level Weibull survival model.
- The effect of volume is modelled continuously using restricted cubic splines.
- We are unable to affirm causality due to the observational nature of the data

Funding Statement

AS is funded by an MRC Strategic Skills Fellowship MR/L01226X/1

For peer review only - http://bmjopen.bmj.com/site/about/guidelines.xhtml

This study was supported by the NIHR Biomedical Research Centre at University Hospitals Bristol NHS Foundation Trust and the University of Bristol. The views expressed in this publication are those of the author(s) and not necessarily those of the NHS, the National Institute for Health Research or the Department of Health and Social Care.

Competing interests statement

We declare no competing interests

For peer review only

Keywords

Total Hip Arthroplasty, Volume, Revision, Within surgeon effects, Between surgeon effects.

For peer review only - http://bmjopen.bmj.com/site/about/guidelines.xhtml

Introduction

Centralisation and specialisation in medical care are advocated to optimise a theorised volume-outcome relationship. In arthroplasty, the volume-outcome relationship has been investigated with respect to outcomes including surgical revision,[1-6] mortality,[1 6-9] patient reported outcome measures (PROMS),[10] and complications[6 11-14] where volume is measured either by surgeon[1-6] or hospital annual volume.[15] Given the technical requirements of arthroplasty, a strong argument for specialisation based on surgical volume and the risk of revision arthroplasty would exist if volume was causally related to outcome. The evidence to support this assertion is surprisingly sparse and has methodological limitations.[1-6] The principal limitation is the failure to distinguish between- and within-consultant effects.

Differentiating between- and within-consultant effects is crucial to interpreting the data. A between-consultant effect is essentially a cross-sectional analysis that compares the performance of one consultant against another and is highly likely to be confounded by centre level effects.[16] A within-consultant effect is based on individual time series data and compares changes of volume across time within the same consultant. Correspondingly, within effects can be interpreted more strongly, as the effect of changing a consultant's personal volume, assuming centre-level factors remain relatively constant over the short term analysis period.

The concept of between- and within-effects is well known in epidemiology, and the ecological fallacy is one example. For example, in a standard (single-level) regression analysis, we may observe a positive association between red meat consumption and life expectancy across several countries with differing levels of development. A between- and within-decomposition would reveal a positive between-country correlation, which is explained by the level of development, and a negative within-country association i.e. individuals within a country who eat more red meat have a lower life expectancy. The

For peer review only - http://bmjopen.bmj.com/site/about/guidelines.xhtml

decomposition of the volume effect into a between- and within-effect is similar i.e. the between-consultant effect is explained by factors, other than volume, which are intrinsic to those consultants and the hospital where they are based ("centre effects"), whereas the within-consultant effect represents the consequence of consultants individually changing their personal volume assuming that other factors remain constant.

In order to facilitate a between- and within-effects analysis, consultant volume needs to be assessed continuously across time. Allowing volume to vary over time is computationally intensive, but responds to variation in demand and capacity to deliver arthroplasty, specialisation or diversification of professional practice, and is in contrast to previous approaches.[1 4] Additionally, as consultant volume changes, dichotomising the data using arbitrary thresholds, e.g. 12 operations per year,[3] is difficult to justify as a consultant's volume will vary over the time they are observed. Furthermore, the interpretation of the results is less likely to be distorted by the arbitrary placement of the threshold.[17 18]

The aim of this research is to investigate the between- and within-consultant (surgeon) effect of the volume of primary total hip arthroplasty (THA) and the risk of subsequent revision.

For peer review only

Methods

Using data from the National Joint Registry of England, Wales, Northern Ireland and the Isle of Man (NJR), we investigated the association between consultant surgical volume in the year (365-days) prior to the index operation of interest, and the risk of revision in patients undergoing elective THA between 1st April 2003 and 22nd February 2017.

Data source

The NJR commenced data collection in April 2003; at inception, it was mandatory for all THA conducted in the private sector to be entered into the NJR, and from 2011 all THA procedures in the public and private sector were required to be entered into the NJR. A recent national audit of data entered into the NJR between 2014 and 2015 estimated data capture of 95% for primary THA and 91% for revision THA.

Ethical Approval

Pseudo anonymised analysis of NJR data is considered as secondary use of clinical registry data, under HRA guidance this does not require formal ethical approval. The full NJR privacy notice can be found online (http://www.njrcentre.org.uk/njrcentre/About-the-NJR/Privacy-Notice-GDPR).

Patient and public involvement

Patient representatives sit on the committee structure of the National Joint Registry. The research priorities of the National Joint Registry are identified by this committee structure and approved by the patient representatives. Patients were not involved in the setting of the research question or the outcome measures, nor were they involved in designing or implementing this work or interpretation of the results. We are unable to disseminate results of this study directly to study participants due to the anonymous nature of the data. We plan to disseminate our findings to the National Joint Registry, via their communications team, to

For peer review only - http://bmjopen.bmj.com/site/about/guidelines.xhtml

relevant individuals who determine the provision of joint replacement, and to the general population through the local and national press.

Inclusion/exclusion criteria

All consenting patients undergoing THA were eligible for inclusion in the analysis. Patients were included if their patient history was unique and consistent, i.e. contained no duplicates, ipsilateral revision prior to the primary, or currently held in query by the submitting unit. Due to the requirement of reliable date information, patients who were indicated to have died prior to undergoing a procedure, were more than 110 years of age, had undergone a procedure prior to their date of birth, or received a procedure prior to 2003 were excluded. Only primary THA, where the sole indication for operation was osteoarthritis (OA) with unique prosthesis combinations, were included. All metal-on-metal bearing combinations were excluded from the analysis due to the known exceptionally high failure rate in this group.[19 20] Consultants with less than 365-days of data were excluded as were patients who were less than 50 years of age at the date of the index THA, because these cases are highly likely to be due to secondary OA. See Figure 1 and Figure 2 for a detailed breakdown of inclusion criteria.

Primary outcome

The primary outcome of interest is all cause revision after a primary THA. Revision arthroplasty was identified by the inclusion of a revision specific data upload after a primary ipsilateral THA. We note that it is not always the primary surgeon that performs the revision.

Censoring

Patients were censored following death. Death status was established by linking patients to the NHS Personal Demographic tracing service.

For peer review only - http://bmjopen.bmj.com/site/about/guidelines.xhtml

Primary exposure

The primary exposure of interest in this study was the consultant surgical volume of any THA recorded in the NJR in the preceding 365-days prior to the index procedure in consenting patients, and prior to the application of inclusion and exclusion criteria, see Figure 1. We choose a 365 day period as this represents one calendar year, and this effectively integrates out seasonal variation from the volume definition,

Confounding factors

Confounding factors were thematically organised into 5 groups:

1) Patient factors: Age, sex, American Society of Anesthesiologists (ASA) grade, and operation funder.
2) Operation factors: THA fixation, approach, patient position during arthroplasty, anaesthetic type, thromboprophylaxis regime, bearing, and year of primary THA.
3) Centre factors included the setting of the treatment episode (i.e. private or NHS hospital) and surgical centre volume.
4) Consultant based factors included the training status of the primary surgeon performing the operation, whether the responsible consultant was listed in the NJR after to 2008 (i.e. a newly qualified surgeon), the proportion of THA undertaken in the NHS in the preceding 365-days by the consultant (i.e. a public or private hospital surgeon), the proportion of THA procedures undertaken in the previous year compared to all joints recorded by the NJR (i.e. a specialist hip surgeon or a general arthroplasty surgeon).
5) Deprivation factors were based on the English and Welsh indices of multiple deprivation, an area based index of patient socioeconomic status. See Supplementary.Table 1.

For peer review only - http://bmjopen.bmj.com/site/about/guidelines.xhtml

Statistical analyses

Means, standard-deviations and interquartile points were used to describe continuous variables. Frequencies and percentages were used to describe categorical variables. The association between confounding factors and consultant volume was explored by comparing summary statistics between levels of each factor.

Graphical methods including frequency distributions, and empirical cumulative distributions were used to describe the relative frequency and centiles of the volume distributions. The empirical cumulative distribution allows centiles of the distribution to be quickly identified.

The associations between consultant volume in the preceding 365-days of the index procedure and all-cause revision were explored using multi-level parametric (Weibull) survival models.

Between- and within-effects are decomposed using a process known as group mean centring.[21 22] Group mean centring is the process of creating two new variables from the primary exposure. The first variable is the consultant specific mean volume, and the second variable is the deviation of a consultant's volume for a given procedure from their personal average (i.e. consultant-mean centred volume). The between-effect is estimated as the coefficient of a consultant's mean volume, whereas the within-effect is the coefficient of the consultant's deviation from their average.

Continuous variables were modelled used orthogonalized Restricted Cubic Splines (RCS), this also included the volume effect. We iteratively varied the number of knot points and placement strategies, in unadjusted models to optimize fit. The most parsimonious specification of RCS was selected using Akaike Information Criterion (AIC).[23]

Confounding adjustment was conducted incrementally introducing patient, operation, centre, surgeon, and finally, deprivation confounding variable groups. The effect of confounding

For peer review only - http://bmjopen.bmj.com/site/about/guidelines.xhtml

adjustment on the primary exposure of interest was explored and presented at each stage of the model building process allowing the effect of adjustment to be clearly illustrated. All modelling was conducted using the `mestreg` package in Stata 15.1.[24]. The specification of the model is described in more detail in the supplementary material.

Missing data

Given the large data set and small fraction of incomplete cases among the observed (FICO) (89% of all eligible records were included in the analysis), any improvement in efficiency from multiple imputation is likely to be negligible, and a complete-cases analysis would provide unbiased results.[25 26] Therefore, we have assumed the reason for missingness is independent of both the outcome and primary exposure of interest.

For peer review only

Results

Between 1st April 2003 and 22nd February 2017, 1,008,619 primary and revision THA procedures were entered in the NJR. After application of inclusion and exclusion criteria, 579,858 primary THA replacement procedures were available for analysis, (Figure 1, Figure 2), including 9238 revised procedures, with 2.5 million years of observation time, with a maximum length of follow up of 13.14 years. Patients were predominately female (61.1%), ASA II (70.4%), treated in public (NHS) hospitals (84.1%), and had a mean age 69.8 years (SD 10.2). See Supplementary.Table 2 and Supplementary Table 3 for full descriptive statistics of the population of patients used in the analysis.

Mean between-consultant volume in the previous year was positively skewed, with only a minority (>97.5% of the empirical cumulative distribution) of consultants performing in-excess of 200 procedures a year, (Figure 3). The median number of procedures conducted across all consultants, for the whole analysis period, in the year prior to an index procedure was 95 (IQR 52, 158) with substantial variability between consultants, (Figure 4). Mean within-consultant volume, for the most part, was symmetrically distributed. There was substantial variability of volume within consultants (median within-consultant SD is 23 (IQR 14.5, 36.5)), (Figure 3), and consultants with larger mean volumes tended to have greater variability, (Figure 4). We also note that the centre IQR of centre volume, is much less variable than consultant volume, but is more negatively skewed at centre level, (Figure 4).

Summary statistics of consultant volume by confounding factors at the index procedure are listed in Supplementary Table 4. Higher volume consultants were observed to treat patients who were younger, with lower ASA grade, who privately funded their THA, and received a uncemented or hybrid prostheses more frequently. They were also more likely to use a posterior approach, place patients in the lateral position, work in the private sector,

For peer review only - http://bmjopen.bmj.com/site/about/guidelines.xhtml

predominately perform hip arthroplasty, and to treat patients from more deprived areas, (Supplementary Table 4).

The effect of confounding adjustment on the volume association was explored using complete cases in estimation of the multi-level Weibull model. The marginal between- and within-consultant effects of volume in the preceding year on hazard of revision and 95% CI are presented in Figure 5, Supplementary Figure 1 and Figure 6, Supplementary Figure 2 respectively.

A near linear 48.8% (95% CI:36.3,61.4) reduction in hazard ratio was observed in crude models for volumes between 1 and 200 procedures annually i.e. from 1.13 (95% CI 1.08, 1.18) to 0.64 (95% CI 0.56, 0.72), with a flattening of risk beyond this, noting that only ~2.5% of consultant recorded more than 200 procedures annually. Despite extensive confounding adjustment, the marginal association of the between-consultant volume effect remained remarkably consistent across the range of volumes observed. In our fully adjusted (deprivation) model, a near linear 43.3% (95% CI 29.1, 57.4) reduction in hazard ratio was observed. The marginal association of the within-consultant volume effect and revision was similarly consistent and showed no evidence (Figure 6) of an association with revision if consultants increased or decreased their volume by ± 50 procedures per year (HR ~1 and 95% CI includes zero).

For peer review only - http://bmjopen.bmj.com/site/about/guidelines.xhtml

Discussion

We provide novel insights into the volume-outcome relationship of 579,858 elective THA patients using a between- and within-decomposition to analyse the association of consultant volumes on revision. Between different consultants, the volume of arthroplasty in the previous year is associated with a near linear 49% and 43% reduction in hazard ratio, between 1 and 200 procedures with revision THA in crude and fully adjusted models respectively. Within the same consultant we demonstrate that there is no evidence of an association between volume of THA in the previous year and risk of revision.

Uniquely, we use a time-varying volume specification that facilitates the decomposition of between- and within-consultant effects. We suggest the within-consultant effect is much closer to the causal interpretation desired by many policymakers, and failure of research to recognise the difference amongst between- and within-effects may lead to erroneous policy decisions and unintended consequences.

We demonstrate that optimal between-consultant results are reached when the consultant volumes in the previous year are approximately 200 procedures. We suggest these factors are not causally related to volume, but rather due to unmeasured surgeon, patient and/or centre factors. There is no evidence to suggest that consultants should change their personal volume in the hope of improving their outcomes or that there is an arbitrary threshold where the outcome of results become good.

Whilst the results appear contradictory compared to previous research i.e. no threshold volume effect, the differences may be explained by the method of analysis, i.e. single-level models vs. multi-level model, and interpretation of results, i.e. separation of between- and within-consultant volume effects. Previous analyses are single-level analyses assume that all procedures are independent of one another, so that a low-volume consultant would achieve

For peer review only

the results of a high-volume consultant if they could instantly increase their personal volume. The interpretation of a single-level model is similar to that of the between-consultant interpretation. Whilst this may be an attractive interpretation for policymakers, it fails to recognise the complexity of the data and processes observed, and that there are many factors, intrinsic to each consultant and the centre or centres in which they work, that predispose them to be either low- or high-volume surgeons e.g. fellowship trained, threshold for revision, or unit organisation.

This study has a number of strengths and limitations. Strengths include: 1) our unique decomposition of between- and within-consultant which we believe provides a more actionable interpretation for policy makers. 2) Time-varying consultant volume, which is independent of the index procedure, and allows for stronger inferences. 3) OA was the only indication for arthroplasty, which we believe represents a "best case scenario", and the volume effect will only be attenuated by the inclusion of other diagnoses. 4) We demonstrate the use of RCS to model volume effects, which ensures flexibility and a smooth continuous function, emphasising the lack of any threshold in the volume effect. 5) The study is significantly, 10 times larger, than any other published study on this topic,[1-6] with a maximal follow-up period of more than 13 years. 6) we have conducted extensive case-mix adjustment and illustrate that both between- and within-effects are insensitive to our measured confounding factors.

Despite the many strengths, this study has a number of limitations. 1) The analysis and decomposition of between- and within-consultant effects is more complex than traditional analyses assume, and requires careful interpretation. 2) Despite the independent nature of the volume of arthroplasty calculation prior to the index procedure, we still see anticipated associations, i.e. younger patients, patients with lower ASA scores, patients receiving uncemented implants and patients operated on in the private sector all tend to be treated by

For peer review only - http://bmjopen.bmj.com/site/about/guidelines.xhtml

higher volume consultants. Consultants specialising in THA and working principally within the private sector accrue higher volumes. These features suggest a strong propensity in practice to treat similar patients and raises the possibility of lagged correlation in unmeasured confounders.[27] 3) The use of a single indication for arthroplasty, namely OA, may limit the generalisability of results particularly in regard to Black or Asian ethnicities where OA is not as dominant an indication for THA.[28] 4) The use of RCS requires extensive sensitivity analyses to ensure knot points are placed optimally, and that results are not sensitive to knot placement. 5) Calculation of the time-varying volume specification of THA in the previous year is computationally intensive and requires significant parallelisation before analyses can be started. 6) Our covariates are unlikely to capture important centre differences in staffing, organization and policy in the management of THAs. 7) Volume does not fully encapsulate experience or expertise that may have been acquired prior to the inception of the register for example during fellowships or from working in other healthcare systems, or the potential persistent effect of learnt experience. 8) Revision surgery is only one possible endpoint and other endpoints including Patient Reported Outcome Measures (PROMs) or other adverse events may also be important to patients. 9) We only considered a multi-level Weibull model to model the baseline hazard; other more flexible functional forms may accommodate the baseline hazard more appropriately, however model convergence is always a challenge.

We suggest the within-consultant effect from the multi-level regression is much closer to the causal interpretation required by consultants, patients, and policymakers i.e. what is the effect of changes in personal volume on the hazard of revision THA? This is not to say the between-effect is not of interest to policy makers, but to say that the between-effect suggests that there are intrinsic differences between high and low volume consultants i.e. expertise, where higher volume consultants tend to have better outcomes, but these differences cannot be attributed to volume per se. We suggest our analyses illustrate "State vs Trait" behaviour.

For peer review only - http://bmjopen.bmj.com/site/about/guidelines.xhtml

Where between-consultant association illustrates the "traits" of surgeons, and within-consultant associations illustrate their "state". This is to say traits of experienced high volume surgeons with good outcomes are unaffected by changes to their personal volume. Conversely, low volume inexperienced arthroplasty surgeons who transiently increase their personal volume do not improve their outcomes.

Conclusion

In summary, using data from the largest arthroplasty register in the world,[29] we have demonstrated that there is no within-consultant association between surgical volume in the previous year and the risk of revision in patients undergoing primary THA for OA. Whereas there is strong evidence to suggest higher volume consultants tend to have better outcomes for reasons that are unlikely to be due to the volume of arthroplasty in the previous year per se.

The results from this study have profound implications for quality improvement within healthcare. Encouraging consultants to undertake a minimum number of procedures under the guise of raising standards could be counterproductive and may only serve to expose patients to increased risk of revision by low or previously low volume consultants. Centralisation and specialisation of THA in consultants who for reasons, not including volume, can undertake a greater number of procedures is likely to benefit patients and reduce the revision burden overall. Encouraging or training low volume consultants to use prosthesis combinations with better outcomes may be more effective methods of improving outcomes for patients.

Importantly, the combined use of an independent time-varying consultant volume with multi-level (between- and within-) regression modelling allows results to be interpreted more clearly than a cross-sectional analysis of conventional observational data. Our work highlights the importance of appropriate methodology, and whilst this may fall short of being definitively causal, results from a randomised experiment are unlikely to be feasible in this

For peer review only - http://bmjopen.bmj.com/site/about/guidelines.xhtml

population. We believe this represents the best available evidence to guide policy formulation.

List of abbreviations

THA: Total Hip Arthroplasty

PROMS: patient reported outcome measures

NJR: National Joint Registry of England, Wales, Northern Ireland and the Isle of Man

OA: Osteoarthritis

ASA: American Society of Anesthesiologists

NHS: National Health Service

RCS: Restricted Cubic Splines

AIC: Akaike Information Criterion

FICO: Fraction of incomplete cases among the observed

IQR: Interquartile Range

Author Contributions

AS, FS, YBS, AJP, MRW, and AWB were responsible for the study design, AS conducted the data analysis. AS, FS, YBS, AJP, MRW, and AWB were responsible for interpreting the data. AS, FS, YBS, AJP, MRW, and AWB prepared and edited and approved the final manuscript.

Role of the funding source

The Medical Research Council or National Institute of Health Research had no role in the design and conduct of the study; collection, management, analysis, and interpretation of the data; preparation, review, or approval of the manuscript; and decision to submit the manuscript for publication

For peer review only

Availability of data and material

Access to the data can be made via research requests to the National Joint Registry of England, Wales, Northern Ireland and the Isle of Man. Full details can be found at http://www.njrcentre.org.uk/njrcentre/Research/Research-requests

Acknowledgements

We thank the patients and staff of all the hospitals who have contributed data to the National Joint Registry. We are grateful to the Healthcare Quality Improvement Partnership, the National Joint Registry Steering Committee, and staff at the National Joint Registry for facilitating this work. The views expressed represent those of the authors and do not necessarily reflect those of the National Joint Registry Steering Committee or Healthcare Quality Improvement Partnership, who do not vouch for how the information is presented.

For peer review only

Figure Titles

Figure 1: Flow chart of participant inclusion and exclusion criteria

Figure 2: Flow chart of participant missing data

Figure 3: Empirical cumulative distribution and frequency distribution of (Between) mean consultant volume and (Within) individual centred volume of hip arthroplasty in the previous 365-days.

Foot Note

Grey horizontal hashed lines indicate the 2.5th, 25th, 50th, 75th, and 97.5th centile of the distribution, vertical hashed lines indicate mean and centred consultant volume at 2.5th, 25th, 50th, 75th, and 97.5th centiles respectively.

Figure 4: Mean, interquartile range, and 95th centile range of consultant and centre volume of hip arthroplasty in the previous 365-days recorded in the NJR by individual consultant, and individual unit respectively.

Figure 5: ***Between-consultant*** *Marginal association of hip surgical volume in the preceding 365-days and hazard of revision arthroplasty unadjusted (M1) and adjusted (M5) for confounding factors in a multi-level model (MLM).*

Foot Note.

Patient factors include sex, ASA grade, funder. Operation confounding factors include fixation, approach, position, anaesthetic, mechanical and chemical thromboprophylaxis, bearing, and year of operation. Centre confounding factors include hospital location and centre volume in the preceding 365-days. Surgeon confounding factors included; lead operating surgeon, listing of a surgeon within NJR prior to 2008, the proportion of NHS

For peer review only - http://bmjopen.bmj.com/site/about/guidelines.xhtml

cases in the preceding year, and proportion hip arthroplasty procedures undertaken in the previous year.

Figure 6: ***Within-consultant*** *Marginal association of hip surgical volume in the preceding 365-days and hazard of revision arthroplasty unadjusted (M1) and adjusted (M5) for confounding factors in a multi-level model (MLM).*

Foot Note.

Patient factors include sex, ASA grade, funder. Operation confounding factors include fixation, approach, position, anaesthetic, mechanical and chemical thromboprophylaxis, bearing, and year of operation. Centre confounding factors include hospital location and centre volume in the preceding 365-days. Surgeon confounding factors included; lead operating surgeon, listing of a surgeon within NJR prior to 2008, the proportion of NHS cases in the preceding year, and proportion hip arthroplasty procedures undertaken in the previous year.

For peer review only

1. Kreder HJ, Deyo RA, Koepsell T, et al. Relationship between the volume of total hip replacements performed by providers and the rates of postoperative complications in the state of Washington. *J Bone Joint Surg Am* 1997;79(4):485-94.
2. Kreder HJ, Williams JI, Jaglal S, et al. Are complication rates for elective primary total hip arthroplasty in Ontario related to surgeon and hospital volumes? A preliminary investigation. *Can J Surg* 1998;41(6):431-7.
3. Losina E, Barrett J, Mahomed NN, et al. Early failures of total hip replacement: effect of surgeon volume. *Arthritis Rheum* 2004;50(4):1338-43. doi: https://dx.doi.org/10.1002/art.20148
4. Manley M, Ong K, Lau E, et al. Effect of volume on total hip arthroplasty revision rates in the United States Medicare population. *J Bone Joint Surg Am* 2008;90(11):2446-51. doi: https://dx.doi.org/10.2106/JBJS.G.01300
5. Khatod M, Cafri G, Namba RS, et al. Risk factors for total hip arthroplasty aseptic revision. *J Arthroplasty* 2014;29(7):1412-7. doi: https://dx.doi.org/10.1016/j.arth.2014.01.023
6. Ravi B, Jenkinson R, Austin PC, et al. Relation between surgeon volume and risk of complications after total hip arthroplasty: propensity score matched cohort study. *Bmj* 2014;348:g3284. doi: https://dx.doi.org/10.1136/bmj.g3284
7. Katz JN, Losina E, Barrett J, et al. Association between hospital and surgeon procedure volume and outcomes of total hip replacement in the United States medicare population. *J Bone Joint Surg Am* 2001;83-A(11):1622-9.
8. Lavernia CJ. Hemiarthroplasty in hip fracture care: effects of surgical volume on short-term outcome. *J Arthroplasty* 1998;13(7):774-8.
9. Lavernia CJ, Guzman JF. Relationship of surgical volume to short-term mortality, morbidity, and hospital charges in arthroplasty. *J Arthroplasty* 1995;10(2):133-40.
10. Katz JN, Phillips CB, Baron JA, et al. Association of hospital and surgeon volume of total hip replacement with functional status and satisfaction three years following surgery. *Arthritis Rheum* 2003;48(2):560-8. doi: https://dx.doi.org/10.1002/art.10754
11. Solomon DH, Losina E, Baron JA, et al. Contribution of hospital characteristics to the volume-outcome relationship: dislocation and infection following total hip replacement surgery. *Arthritis Rheum* 2002;46(9):2436-44. doi: https://dx.doi.org/10.1002/art.10478
12. Battaglia TC, Mulhall KJ, Brown TE, et al. Increased surgical volume is associated with lower THA dislocation rates. *Clin Orthop* 2006;447:28-33. doi: https://dx.doi.org/10.1097/01.blo.0000218743.99741.f0
13. Khatod M, Barber T, Paxton E, et al. An analysis of the risk of hip dislocation with a contemporary total joint registry. *Clin Orthop* 2006;447:19-23. doi: https://dx.doi.org/10.1097/01.blo.0000218752.22613.78
14. Yasunaga H, Tsuchiya K, Matsuyama Y, et al. High-volume surgeons in regard to reductions in operating time, blood loss, and postoperative complications for total hip arthroplasty. *J Orthop Sci* 2009;14(1):3-9. doi: https://dx.doi.org/10.1007/s00776-008-1289-4
15. Judge A, Chard J, Learmonth I, et al. The effects of surgical volumes and training centre status on outcomes following total joint replacement: analysis of the Hospital Episode Statistics for England. *J Public Health (Oxf)* 2006;28(2):116-24. doi: https://dx.doi.org/10.1093/pubmed/fdl003

For peer review only - http://bmjopen.bmj.com/site/about/guidelines.xhtml

16. Hodsman A, Ben-Shlomo Y, Roderick P, et al. The 'Centre Effect' in Nephrology: What Do Differences between Nephrology Centres Tell Us about Clinical Performance in Patient Management? *Nephron Clin Pract* 2011;119(1):C10-C17. doi: 10.1159/000321378
17. Orsini N, Greenland S. A procedure to tabulate and plot results after flexible modeling of a quantitative covariate. *Stata J* 2011;11(1):1-29.
18. Greenland S. Avoiding power loss associated with categorization and ordinal scores in dose-response and trend analysis. *Epidemiology* 1995;6(4):450-4. [published Online First: 1995/07/01]
19. Smith AJ, Dieppe P, Howard PW, et al. Failure rates of metal-on-metal hip resurfacings: analysis of data from the National Joint Registry for England and Wales. *Lancet* 2012;380(9855):1759-66. doi: 10.1016/S0140-6736(12)60989-1 [published Online First: 2012/10/06]
20. Smith AJ, Dieppe P, Vernon K, et al. Failure rates of stemmed metal-on-metal hip replacements: analysis of data from the National Joint Registry of England and Wales. *Lancet* 2012;379(9822):1199-204. doi: 10.1016/S0140-6736(12)60353-5 [published Online First: 2012/03/16]
21. Neuhaus JM, McCulloch CE. Separating between- and within-cluster covariate effects by using conditional and partitioning methods. *J Roy Stat Soc B* 2006;68:859-72. doi: DOI 10.1111/j.1467-9868.2006.00570.x
22. Skrondal A, Rabe-Hesketh S. Generalized Latent Variable Modelling: Multilevel, Longitudinal , and Structural Equation Models. Boca Raton: Chapman & Hall/CRC 2004.
23. Akaike H. Citation Classic - a New Look at the Statistical-Model Identification. *Cc/Eng Tech Appl Sci* 1981(51):22-22.
24. Stata Statistical Software: Release 14 [program]. College Station, TX: StataCorp LP, 2015.
25. White IR, Carlin JB. Bias and efficiency of multiple imputation compared with complete-case analysis for missing covariate values. *Stat Med* 2010;29(28):2920-31. doi: 10.1002/sim.3944 [published Online First: 2010/09/16]
26. Schafer JL, Graham JW. Missing data: Our view of the state of the art. *Psychol Methods* 2002;7(2):147-77. doi: 10.1037//1082-989x.7.2.147
27. Bellemare MF, Masaki T, Pepinsky TB. Lagged Explanatory Variables and the Estimation of Causal Effect. *J Polit* 2017;79(3):949-63. doi: 10.1086/690946
28. Smith MC, Ben-Shlomo Y, Dieppe P, et al. Rates of hip and knee joint replacement amongst different ethnic groups in England: an analysis of National Joint Registry data. *Osteoarthritis Cartilage* 2017;25(4):448-54. doi: 10.1016/j.joca.2016.12.030 [published Online First: 2017/02/06]
29. Malchau H, Garellick G, Berry D, et al. Arthroplasty implant registries over the past five decades: Development, current, and future impact. *J Orthop Res* 2018;36(9):2319-30. doi: 10.1002/jor.24014 [published Online First: 2018/04/18]

For peer review only - http://bmjopen.bmj.com/site/about/guidelines.xhtml

Figure 1. Flow chart of participant inclusion and exclusion criteria

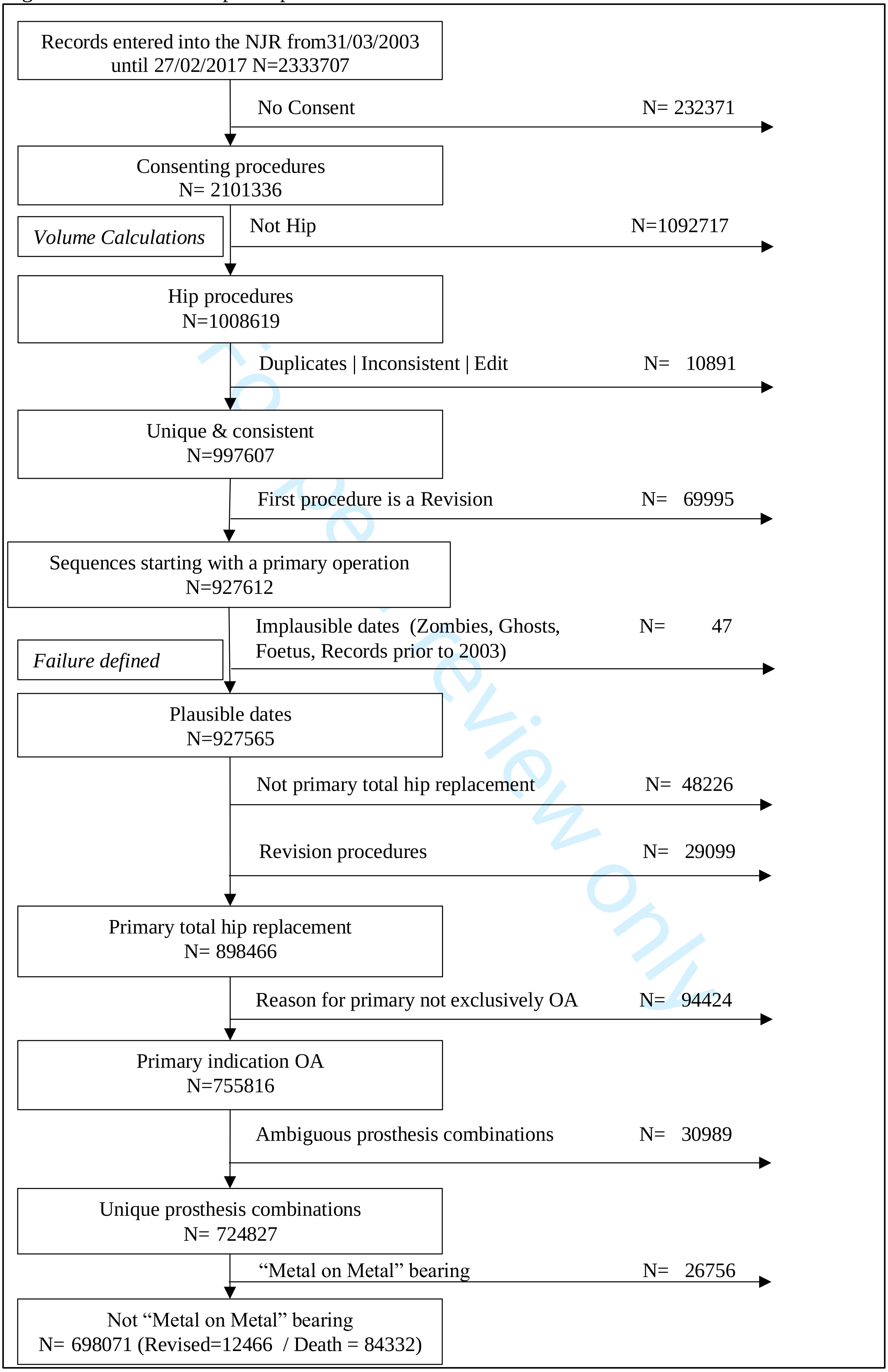

For peer review only

Figure 2. Flow chart of participant missing data

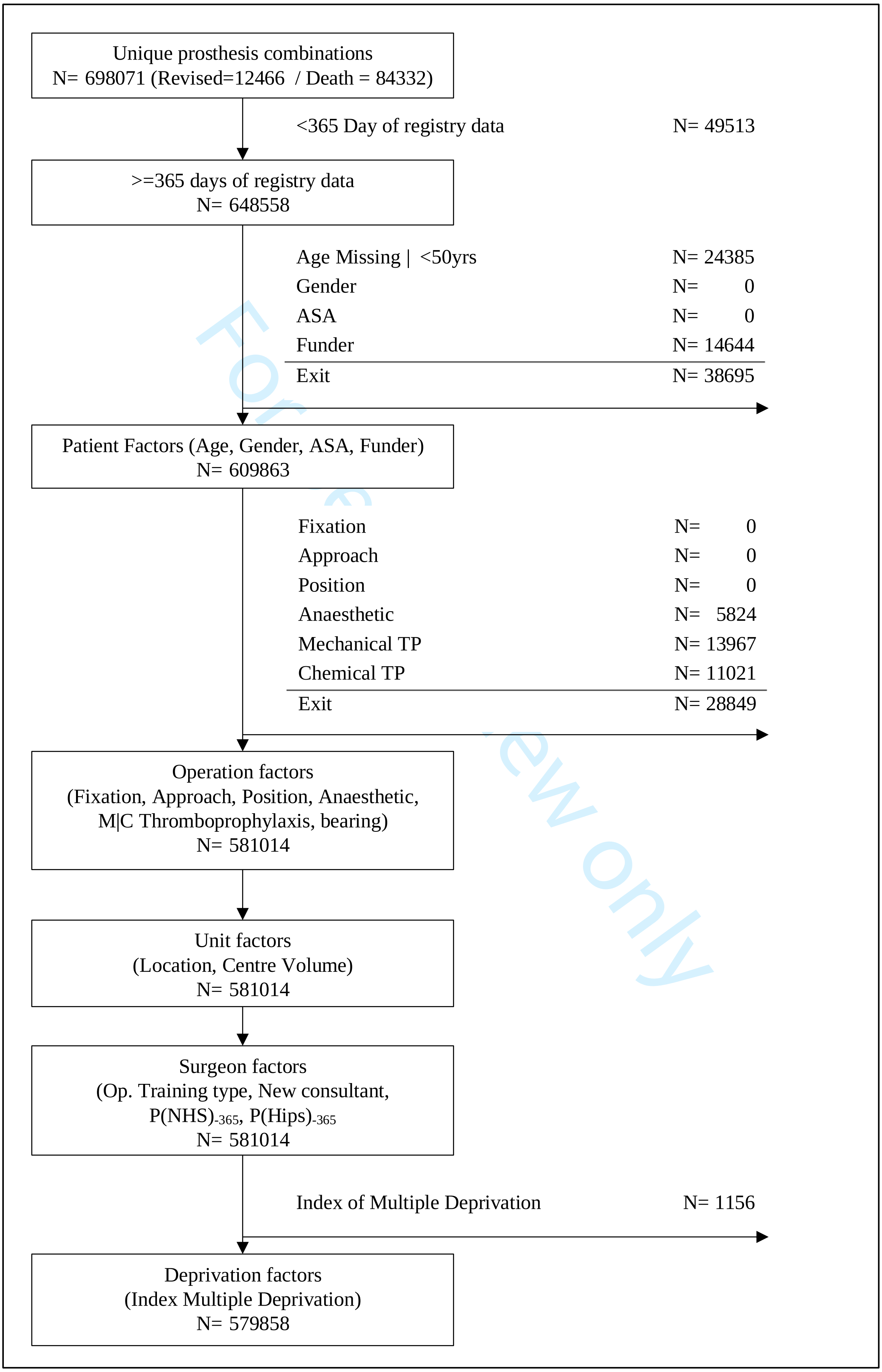

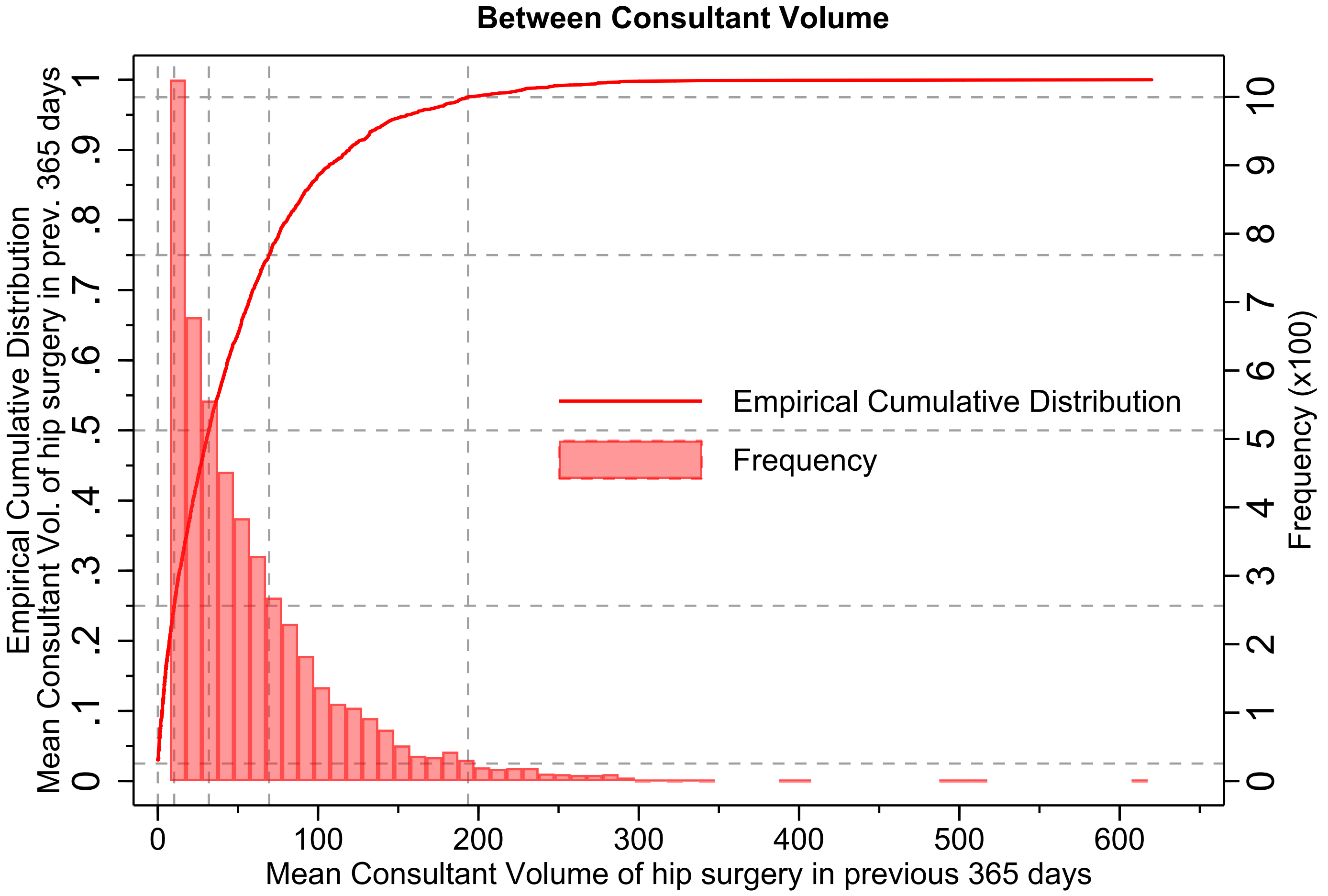
Between Consultant Volume
Empirical Cumulative Distribution
Mean Consultant Vol. of hip surgery in prev. 365 days
Frequency (x100)
Empirical Cumulative Distribution
Frequency
Mean Consultant Volume of hip surgery in previous 365 days
0 .1 .2 .3 .4 .5 .6 .7 .8 .9 1
0 1 2 3 4 5 6 7 8 9 10
0 100 200 300 400 500 600

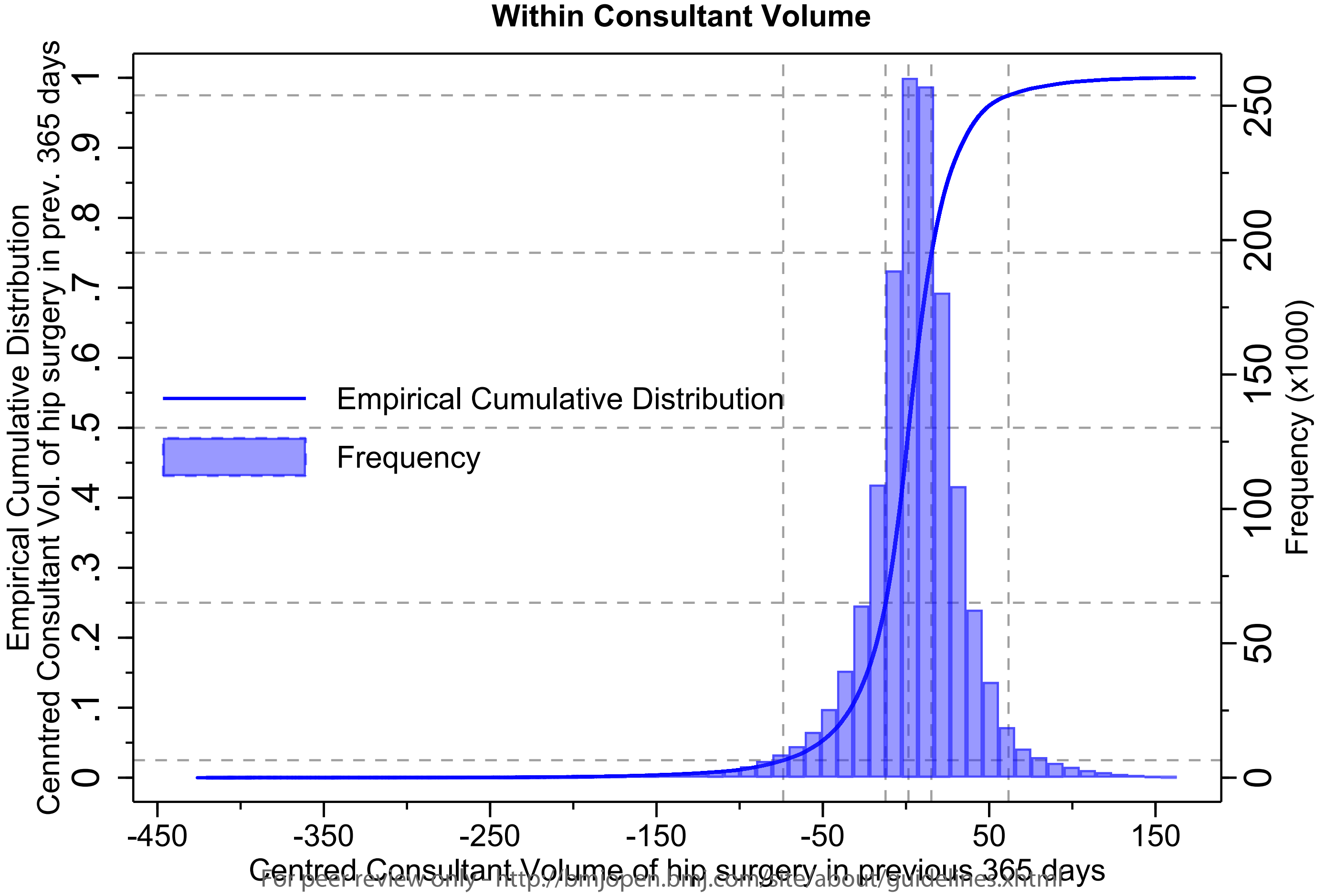
Within Consultant Volume
Empirical Cumulative Distribution
Cenntred Consultant Vol. of hip surgery in prev. 365 days
Frequency (x1000)
Empirical Cumulative Distribution
Frequency
Centred Consultant Volume of hip surgery in previous 365 days
0 .1 .2 .3 .4 .5 .6 .7 .8 .9 1
0 50 100 150 200 250
-450 -350 -250 -150 -50 50 150

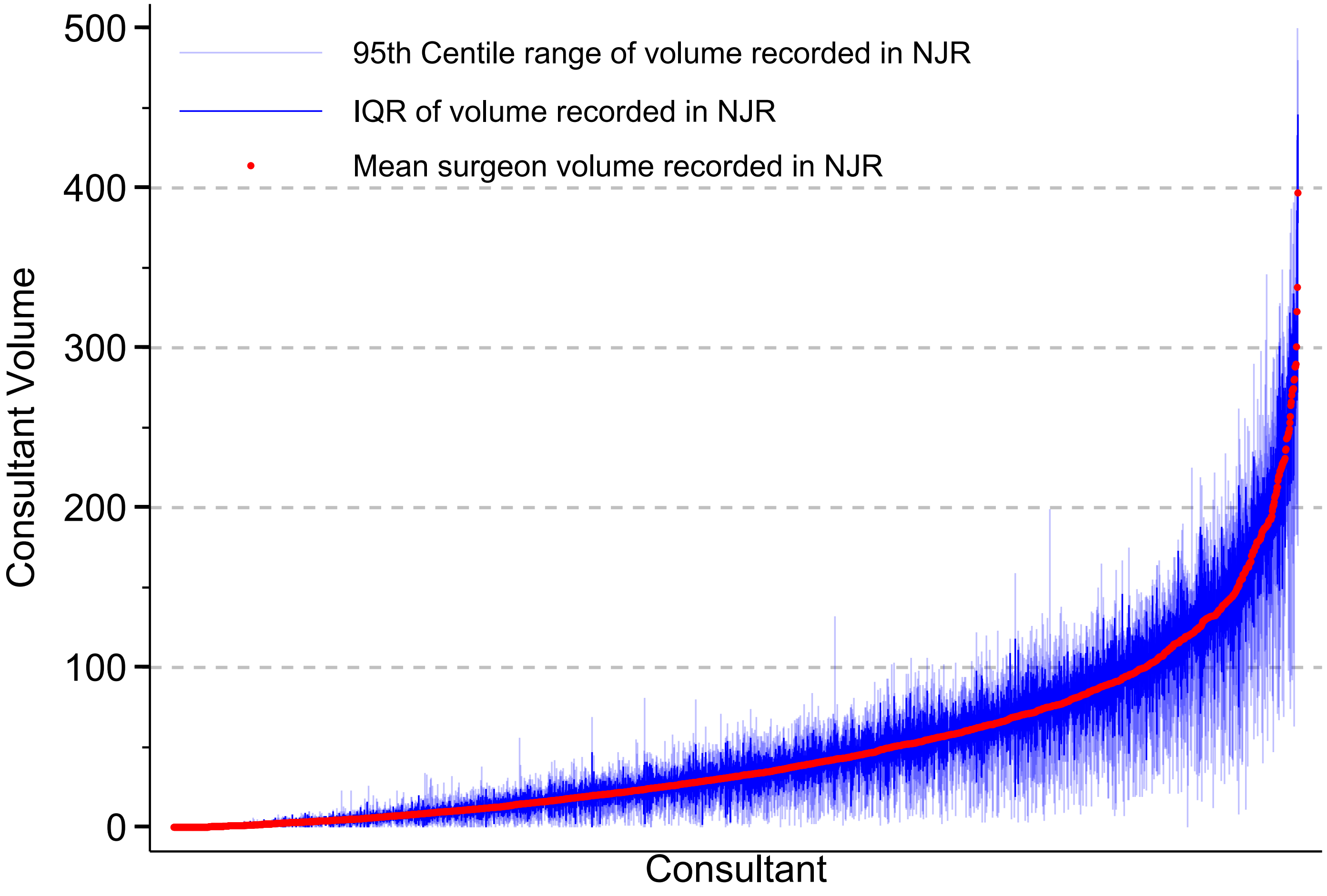
95th Centile range of volume recorded in NJR
IQR of volume recorded in NJR
Mean surgeon volume recorded in NJR
Consultant Volume
0
100
200
300
400
500
Consultant

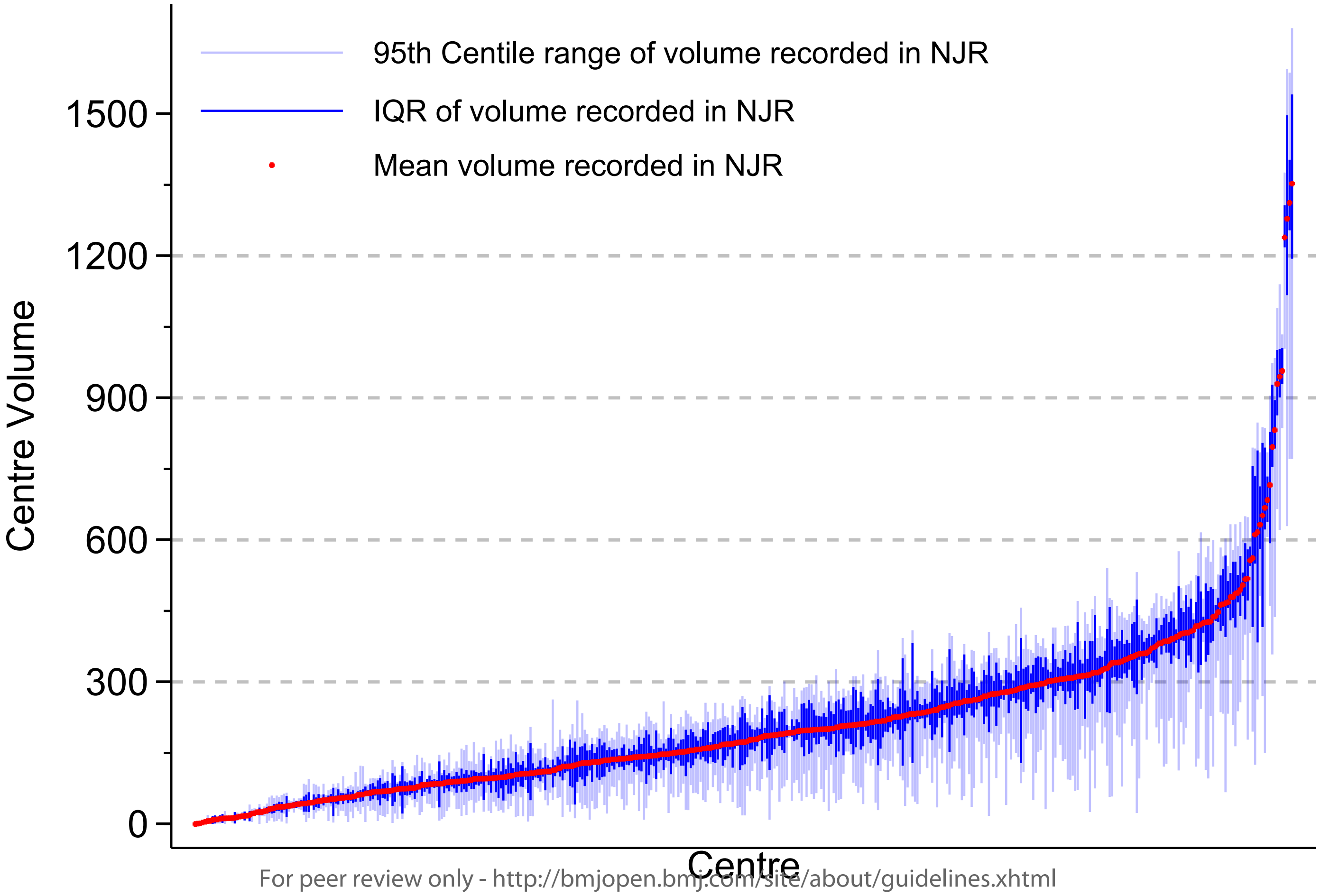
95th Centile range of volume recorded in NJR
IQR of volume recorded in NJR
Mean volume recorded in NJR
Centre Volume
0
300
600
900
1200
1500
Centre

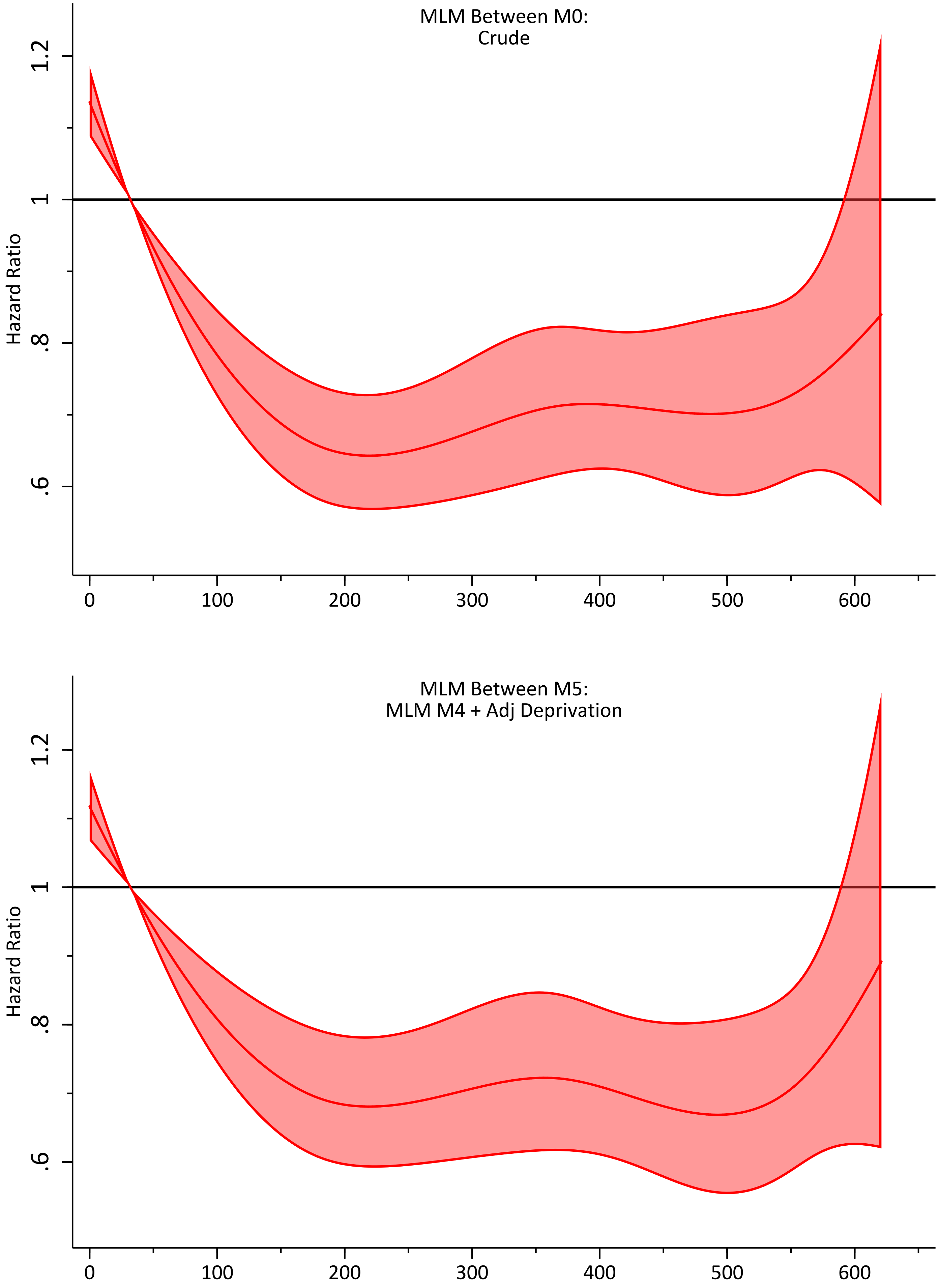

For peer review only - http://bmjopen.bmj.com/site/about/guidelines.xhtml

Consultant volume of hip surgery in previous 365 days

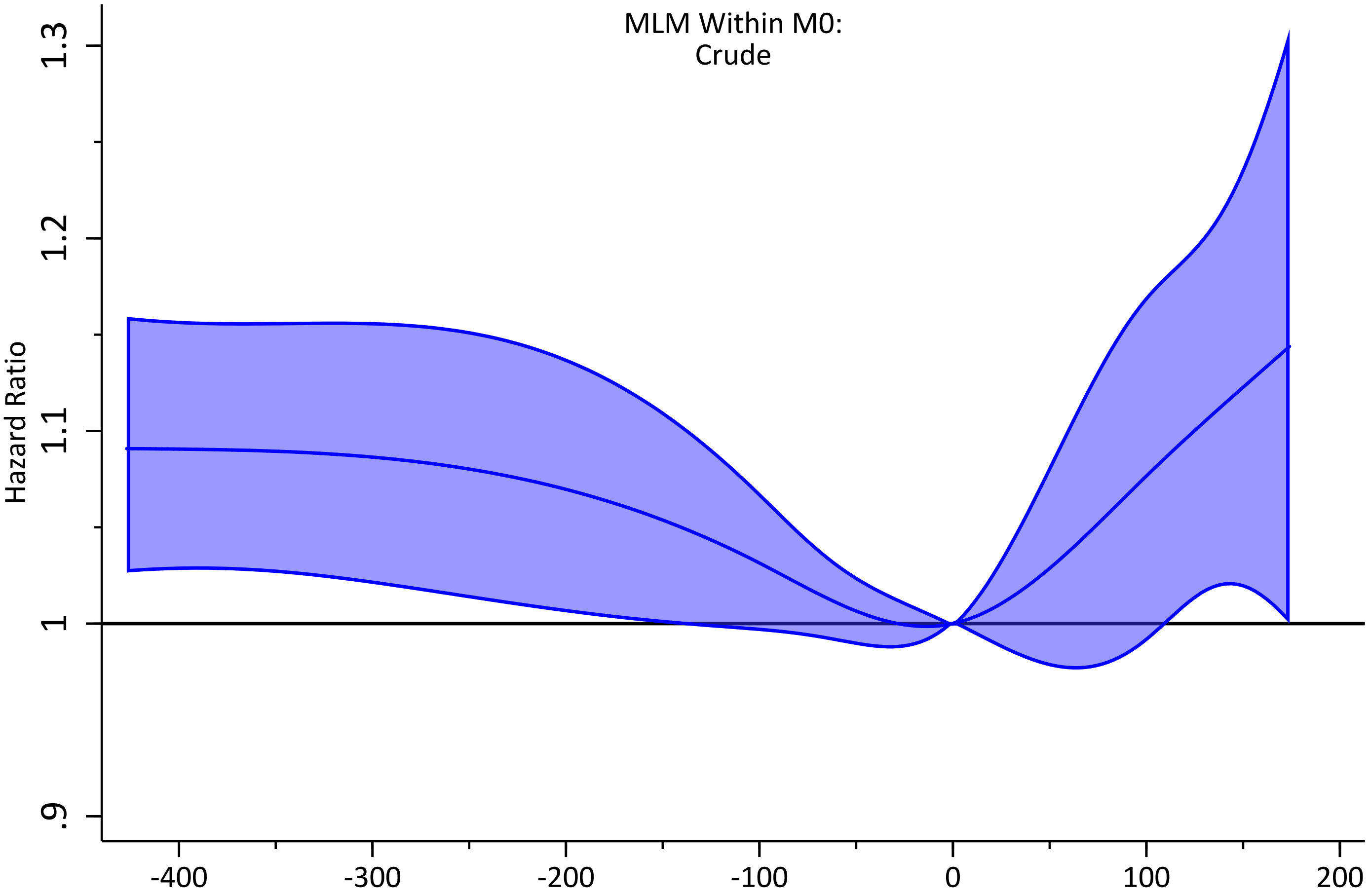

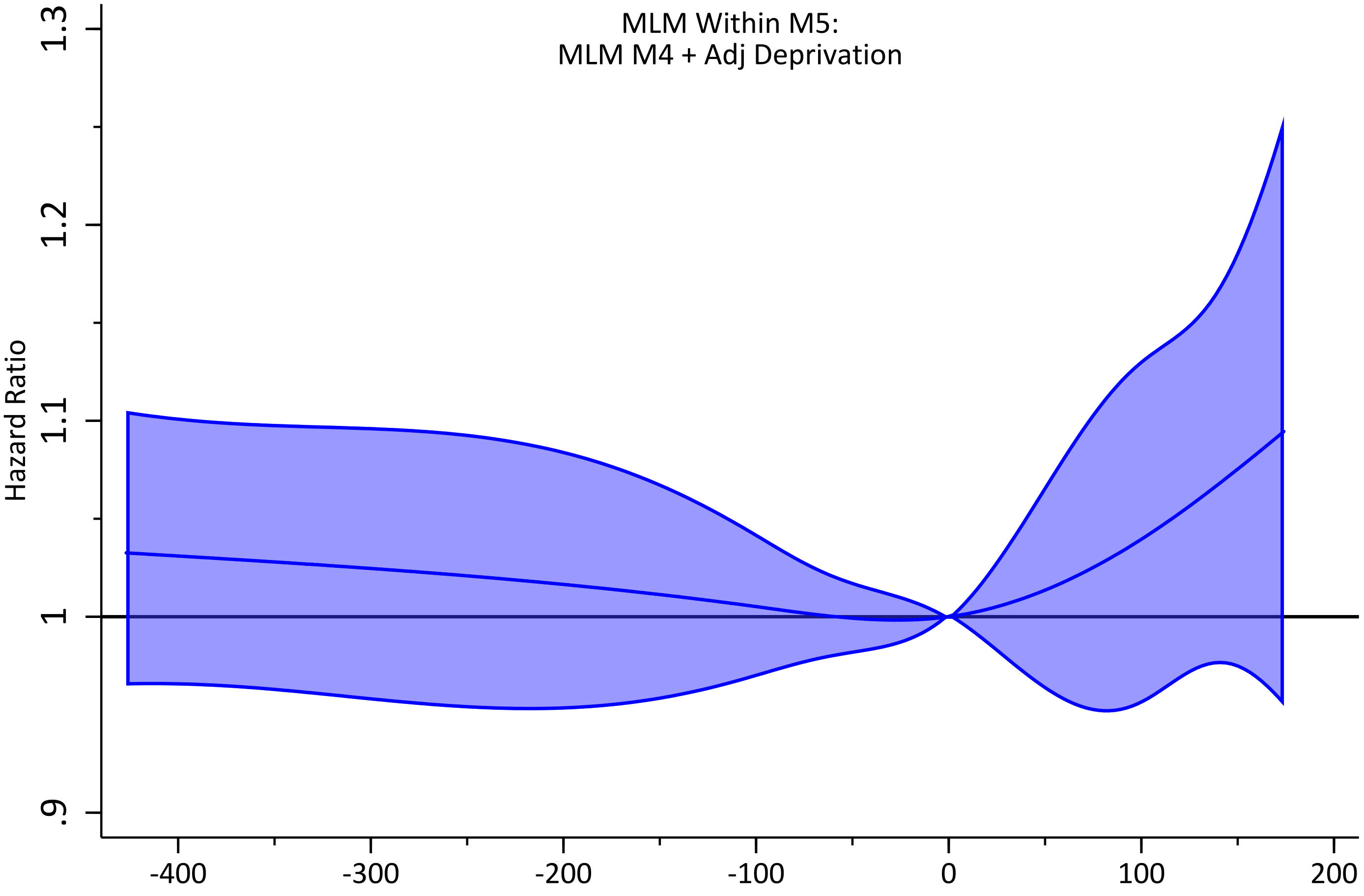

For peer review only - http://bmjopen.bmj.com/site/about/guidelines.xhtml

Consultant volume of hip surgery in previous 365 days

Supplementary Tables

Multilevel Weibull model

Denote by V_{ij} the volume in the 365 days prior to procedure i ($i = 1, \dots, I$) carried out by surgeon j ($j = 1, \dots, J$) and $\bar{V}_j$ the surgeon's average volume over the observation period. A multilevel Weibull model for the association between $h_{ij}(t)$, the hazard of revision at time t for procedure i of surgeon j, and volume is specified as

$$h_{ij}(t) = h_0(t)\exp\left[\boldsymbol{\alpha}S(\bar{V}_j, K_0) + \boldsymbol{\gamma}S\left((\bar{V}_j - V_{ij}), K_1\right) + \boldsymbol{\beta}X_{ij} + Z_j\right]$$

where $h_0(t)$ is the baseline hazard function of a Weibull distribution, $S()$ represents the restricted cubic spline basis, with a vector of K_k knots, $\boldsymbol{\alpha}$ is a vector of coefficients representing the between-consultant volume association, $\boldsymbol{\gamma}$ is a vector of coefficients for the within-consultant volume association, and $\boldsymbol{\beta}$ is a vector of coefficients for confounding factors X_{ij}, and $Z_j \sim N(0, \sigma^2)$ is a surgeon-level random effect.

Supplementary.Table 1: Coding and specification of confounding variables

Variable	Options	Baseline | Centred	Regression Specification
Patient Age	Orthogonalized Restricted Cubic Splines Knots @ knots(50(10)90 102	70	Continuous
Sex	Male / Female	Male	Binary
American Society of Anaesthesiologists (ASA) grade	I / II / III / IV+V	II	MV Binary
Operation funder	Public / Private	Public	Binary
Fixation	Cemented / Uncemented / Hybrid / Reverse Hybrid	Cemented	MV Binary
Approach	Posterior /Other	Posterior	Binary
Patient position during arthroplasty	Lateral / Supine	Lateral	Binary
Anaesthetic type	Spinal / GA / GA & Spinal / GA & Nerve Block/ Epidural / Nerve Block & Spinal / GA & Epidural / None / Epidural & Spinal / Nerve Block / GA & Nerve Block & Spinal / Other	Spinal	MV Binary
Mechanical thromboprophylaxis	TED / TED ICC / ICC / TED FP / FP / TED FP ICC / FP ICC / None	None	MV Binary
Chemical thromboprophylaxis	LMWH / Asprin/ DTI / LMWH Asprin / FXa / LMWH DTI / LMWH FXa / PS / LMWH Warf / Warf / LDH / LMWH PS /None /Other	None	MV Binary
Bearing	CoC / CoM /CoPe /MoC / MoPe / MoPu	MoPE	MV Binary
Year of arthroplasty	Orthogonalised Restricted Cubic Splines Knots @ 0(4.33)13	2004	Continuous
Setting of the treatment	NHS / Treatment Centre / Privates	NHS	MV Binary
Surgical centre volume	Orthogonalised Restricted Cubic Splines Knots @ 0(430)1720	97	Continuous

For peer review only

Training status of the primary surgeon	Consultant / Trainee	Consultant	Binary
Consultant was listed in the NJR prior to 2008	<2008 / >= 2008	<2008	Binary
proportion of THA undertaken in the NHS	Orthogonalized Restricted Cubic Splines Knots @ knots(0(0.25)1	0.7	Continuous
proportion of THA procedures	Orthogonalized Restricted Cubic Splines Knots @ knots(0(0.125)1	0.6	Continuous
Index of Multiple Deprivation (English)	Deciles	Decile #1	MV Binary
Welsh Index of Multiple Deprivation	Deciles	Decile #1	MV Binary

For peer review only

Supplementary.Table 2: Descriptive statistics of continuous variables used in the analysis.

Variable	Mean	(SD)	[25th	50th	75th]	Missing
(Traced) Age at operation date, rounded 2 DP	69.84	(10.2)	[63.56,	70.65,	77.16]	0
Consultant recorded hip volume in previous 365 days	118.36	(97.3)	[52.00,	95.00,	158.00]	0
SD consultant recorded hip volume in previous 365 days by consultant	28.13	(19.8)	[14.48,	23.48,	36.53]	114
Centre recorded hip volume in previous 365 days	370.18	(306.2)	[174.00,	284.00,	435.00]	0
SD centre recorded hip volume in previous 365 days by centre	81.84	(57.5)	[44.69,	65.90,	101.86]	2
Proportion of NHS hip procedures in previous 365 days	0.69	(0.3)	[0.54,	0.74,	0.94]	0
SD Proportion of NHS hip procedures in previous 365 day by consultant	0.10	(0.1)	[0.05,	0.10,	0.14]	114
Proportion of hip procedures in previous 365 days	0.60	(0.2)	[0.46,	0.58,	0.73]	0
SD Proportion of hip procedures in previous 365 days by consultant	0.06	(0.0)	[0.04,	0.06,	0.08]	114

For peer review only

Supplementary.Table 3: Descriptive statistics of categorical variable used in the analysis.

Variable	Factor	N	(%)
Gender	Female	397107	(61.1)
	Male	252852	(38.9)
	Missing	0	(0.0)
ASA Grade	I	94042	(14.5)
	II	457780	(70.4)
	III	95293	(14.7)
	IV +V	2844	(0.4)
	Missing	0	(0.0)
Procedure funded by:	NHS	546727	(84.1)
	Private	88588	(13.6)
	Missing	14644	(2.3)
Method of fixation	Cemented	236554	(36.4)
	Uncemented	260857	(40.1)
	Hybrid	133489	(20.5)
	Reverse Hybrid	19059	(2.9)
	Missing	0	(0.0)
Surgical Approach	Posterior	376818	(58.0)
	Other	273141	(42.0)
	Missing	0	(0.0)
Patient Position	Lateral	595964	(91.7)
	Supine	53995	(8.3)
	Missing	0	(0.0)
Anaesthetic combination	Sp	329954	(50.8)
	GA	141132	(21.7)
	GA Sp	79706	(12.3)
	GA NB	29658	(4.6)
	Epi	18759	(2.9)
	NB Sp	16450	(2.5)
	GA Epi	13235	(2.0)
	None	103	(0.0)
	Epi Sp	5449	(0.8)
	NB	3912	(0.6)
	GA NB Sp	2353	(0.4)
	Other	600	(0.1)
	Missing	8648	(1.3)
Mechanical thromboprophylaxis combination	TED	198597	(30.6)
	TED ICC	128881	(19.8)
	ICC	101050	(15.5)
	TED FP	77150	(11.9)
	FP	58467	(9.0)
	None	33814	(5.2)
	TED FP ICC	26230	(4.0)
	FP ICC	8994	(1.4)
	Missing	16776	(2.6)
Chemical thromboprophylaxis combination	LMWH	397523	(61.2)
	None	57057	(8.8)
	Asprin	44497	(6.8)
	DTI	36394	(5.6)
	LMWH Asprin	29615	(4.6)
	FXa	31768	(4.9)
	LMWH DTI	14628	(2.3)
	LMWH FXa	7391	(1.1)
	PS	4875	(0.8)

For peer review only

	LMWH Warf	3712	(0.6)
	Warf	3264	(0.5)
	LDH	1299	(0.2)
	LMWH PS	1781	(0.3)
	Other	1364	(0.2)
	Missing	14791	(2.3)
Bearing Surface [Head on Acetab.]	CoC	108539	(16.7)
	CoM	1916	(0.3)
	CoPe	119379	(18.4)
	MoC	115	(0.0)
	MoPe	419844	(64.6)
	MoPu	166	(0.0)
	Missing	0	(0.0)
Hospital provider category	NHS	420897	(64.8)
	Treatment Centre	27233	(4.2)
	Private	201829	(31.1)
	Missing	0	(0.0)
Year of operation	04	3783	(0.6)
	05	22638	(3.5)
	06	29726	(4.6)
	07	38141	(5.9)
	08	42083	(6.5)
	09	46407	(7.1)
	10	52725	(8.1)
	11	57465	(8.8)
	12	63544	(9.8)
	13	66043	(10.2)
	14	71634	(11.0)
	15	71316	(11.0)
	16	72761	(11.2)
	17	11693	(1.8)
	Missing	0	(0.0)
Operation training type (Consultant / Lead surgeon / Assisted by) Number in parentheses indicates the same or different consultant	Con.(1)/Con.(1)	520620	(80.1)
	Con.(1)/Con.(1)(?)	18202	(2.8)
	Con.(1)/Con.(2)	15041	(2.3)
	Con.(1)/Jun.(1)/Con.	37414	(5.8)
	Con.(1)/Jun.(1)/Jun.(2)	58682	(9.0)
	Missing	0	(0.0)
Consultant first recorded procedure post 2008 + 365days	Cons. Pre 2009	547791	(84.3)
	Cons. Post 2009	102168	(15.7)
	Missing	0	(0.0)
Index of Multiple Deprivation Deciles (Wales)	1	1945	(0.3)
	2	2460	(0.4)
	3	2854	(0.4)
	4	3183	(0.5)
	5	3668	(0.6)
	6	4351	(0.7)
	7	4596	(0.7)
	8	4498	(0.7)
	9	4294	(0.7)
	10	3630	(0.6)
	Missing	614480	(94.5)
Index of Multiple Deprivation Deciles (England)	1	32697	(5.0)
	2	37030	(5.7)
	3	43721	(6.7)
	4	54200	(8.3)
	5	63862	(9.8)
	6	71498	(11.0)
	7	74777	(11.5)

For peer review only

	8	76272	(11.7)
	9	78245	(12.0)
	10	80544	(12.4)
	Missing	37113	(5.7)

For peer review only

Supplementary.Table 4: Consultant volume summary statistics by potential confounding factors.

Variable	Category	N	Mean	(SD)	[25th	50th	75th]
Age at operation	50 to 55yrs	28129	129.82	(97.4)	[64.00,	108.00,	170.00]
	55 to 60yrs	48289	124.41	(95.9)	[59.00,	103.00,	164.00]
	60 to 65yrs	78757	121.97	(97.5)	[55.00,	99.00,	163.00]
	65 to 70yrs	109204	120.88	(98.6)	[53.00,	97.00,	161.00]
	70 to 75yrs	116901	118.50	(97.9)	[51.00,	95.00,	158.00]
	75 to 80yrs	104115	117.07	(98.9)	[50.00,	93.00,	156.00]
	80 to 85yrs	64385	117.64	(99.4)	[50.00,	93.00,	157.00]
	85 to 90yrs	25244	119.65	(101.7)	[51.00,	94.00,	159.00]
	>90yrs	4834	123.76	(105.2)	[53.00,	97.00,	165.00]
Gender	Female	355154	120.68	(99.3)	[53.00,	97.00,	161.00]
	Male	224704	119.45	(96.9)	[53.00,	96.00,	159.00]
ASA Grade	I	75489	129.62	(105.2)	[56.00,	103.00,	176.00]
	II	414552	120.68	(98.9)	[53.00,	97.00,	160.00]
	III	87278	110.27	(89.1)	[49.00,	88.00,	148.00]
	IV +V	2539	103.76	(80.5)	[46.00,	83.00,	140.00]
Procedure funded by:	NHS	500193	116.38	(94.5)	[52.00,	94.00,	155.00]
	Private	79665	144.25	(117.2)	[63.00,	116.00,	196.00]
Method of fixation	Cemented	209831	100.24	(73.9)	[43.00,	82.00,	140.00]
	Uncemented	231302	123.73	(100.1)	[56.00,	96.00,	160.00]
	Hybrid	121293	149.75	(124.2)	[71.00,	123.00,	192.00]
	Reverse Hybrid	17432	108.24	(68.9)	[57.00,	93.50,	147.00]
Hospital provider category	NHS	371858	113.06	(93.7)	[50.00,	90.00,	151.00]
	Treatment Centre	25296	103.59	(56.0)	[60.00,	100.00,	134.00]
	Private	182704	137.05	(109.5)	[61.00,	109.00,	184.00]
P(NHS) in previous 365-days	<0.25	59325	115.76	(109.8)	[42.00,	85.00,	145.00]
	>=0.25 & <0.5	70297	149.07	(118.0)	[60.00,	115.00,	212.00]
	>=0.5 & <0.75	170720	137.44	(100.4)	[72.00,	117.00,	178.00]
	>=0.75 & <1	278820	103.62	(84.7)	[45.00,	83.00,	140.00]
P(Hip Arthroplasty) in previous 365-days	<0.25	21128	25.62	(21.4)	[10.00,	21.00,	33.00]
	>=0.25 & <0.5	176666	66.41	(40.2)	[36.00,	59.00,	89.00]
	>=0.5 & <0.75	255607	119.99	(67.8)	[69.00,	109.00,	159.00]
	>=0.75 & <1	126204	212.03	(137.2)	[123.00,	188.00,	265.00]
Index of Multiple Deprivation Deciles (Wales)	1	1657	98.69	(78.0)	[39.00,	72.00,	148.00]
	2	2153	98.57	(74.4)	[40.00,	76.00,	142.00]
	3	2522	91.51	(67.8)	[40.00,	71.00,	129.00]
	4	2809	88.34	(68.7)	[38.00,	69.00,	121.00]
	5	3343	91.71	(72.2)	[36.00,	70.00,	130.00]
	6	3961	90.30	(67.8)	[38.00,	70.00,	130.00]
	7	4249	97.31	(70.0)	[43.00,	77.00,	138.00]
	8	4135	98.71	(70.1)	[44.00,	80.00,	139.00]

For peer review only

	9	3947	115.23	(84.7)	[49.00,	92.00,	174.00]
	10	3265	128.15	(85.1)	[56.00,	108.00,	198.00]
Index of Multiple Deprivation Deciles (England)	1	28022	98.15	(75.1)	[45.00,	81.00,	131.00]
	2	32304	101.47	(81.3)	[46.00,	83.00,	135.00]
	3	38370	106.67	(89.1)	[47.00,	85.00,	140.00]
	4	47978	111.39	(88.7)	[51.00,	90.00,	147.00]
	5	57129	118.02	(97.2)	[53.00,	94.00,	155.00]
	6	64237	124.51	(101.9)	[56.00,	100.00,	164.00]
	7	67491	125.95	(103.4)	[57.00,	101.00,	166.00]
	8	68831	127.19	(103.2)	[57.00,	103.00,	170.00]
	9	70537	130.69	(105.0)	[59.00,	106.00,	174.00]
	10	72918	134.51	(108.8)	[60.00,	111.00,	179.00]
Surgical Approach	Posterior	342849	144.30	(108.1)	[71.00,	121.00,	189.00]
	Other	237009	85.35	(68.7)	[37.00,	69.00,	115.00]
Patient Position	Lateral	535280	122.45	(99.0)	[55.00,	99.00,	164.00]
	Supine	44578	93.27	(87.1)	[40.00,	70.00,	112.00]
Anaesthetic combination	Sp	305186	116.39	(88.7)	[56.00,	98.00,	154.00]
	GA	125359	109.36	(87.5)	[46.00,	86.00,	151.00]
	GA Sp	71916	162.93	(131.8)	[68.00,	128.00,	221.00]
	GA NB	25213	105.09	(83.1)	[44.00,	84.00,	144.00]
	Epi	16568	95.02	(84.9)	[38.00,	71.00,	132.00]
	NB Sp	15451	132.51	(151.0)	[53.00,	86.00,	137.00]
	GA Epi	9820	130.50	(98.4)	[52.00,	102.00,	202.00]
	None	59	115.80	(78.9)	[49.00,	103.00,	167.00]
	Epi Sp	4041	104.14	(79.2)	[43.00,	83.00,	155.00]
	NB	3524	101.01	(83.8)	[48.00,	79.00,	133.00]
	GA NB Sp	2188	163.59	(115.9)	[78.00,	149.00,	230.00]
	Other	533	111.07	(112.9)	[44.00,	85.00,	143.00]
Mechanical thromboprophylaxis combination	TED	183510	115.60	(104.1)	[49.00,	92.00,	149.00]
	TED ICC	119415	118.20	(101.3)	[51.00,	90.00,	151.00]
	ICC	92340	109.16	(77.2)	[52.00,	91.00,	151.00]
	TED FP	67618	146.91	(118.8)	[67.00,	120.00,	187.00]
	FP	53375	132.33	(86.1)	[62.00,	122.00,	190.00]
	None	31757	117.05	(79.4)	[53.00,	100.00,	170.00]
	TED FP ICC	23445	115.61	(84.4)	[58.00,	91.00,	151.00]
	FP ICC	8398	103.40	(81.1)	[48.00,	83.00,	144.00]
Chemical thromboprophylaxis combination	LMWH	362081	113.44	(94.4)	[50.00,	91.00,	150.00]
	None	53791	129.35	(106.3)	[54.00,	99.00,	176.00]
	Asprin	38635	125.01	(88.2)	[56.00,	110.00,	170.00]
	DTI	34669	117.68	(78.4)	[59.00,	100.00,	163.00]
	LMWH Asprin	26428	146.37	(111.5)	[64.00,	125.00,	207.00]
	FXa	30026	153.06	(120.9)	[73.00,	122.00,	194.00]
	LMWH DTI	13840	138.70	(127.3)	[56.00,	99.00,	186.00]
	LMWH FXa	7019	128.35	(122.9)	[58.00,	99.00,	159.00]
	PS	4553	102.17	(64.2)	[50.00,	91.00,	144.00]
	LMWH Warf	3028	121.75	(98.5)	[55.00,	103.00,	164.00]

For peer review only

	Warf	2616	115.38	(73.6)	[57.00,	117.00,	152.00]
	LDH	677	91.82	(86.9)	[28.00,	47.00,	168.00]
	LMWH PS	1360	104.65	(77.3)	[49.00,	86.00,	160.00]
	Other	1135	120.09	(117.3)	[56.00,	88.00,	154.00]
Bearing Surface [Head on Acetab.]	CoC	89379	125.46	(90.5)	[61.00,	104.00,	166.00]
	CoM	1728	186.67	(141.7)	[69.00,	121.00,	286.50]
	CoPe	106359	138.64	(125.1)	[60.00,	104.00,	178.00]
	MoC	100	103.72	(88.3)	[41.50,	68.50,	140.50]
	MoPe	382139	113.55	(90.3)	[49.00,	92.00,	154.00]
	MoPu	153	123.27	(46.2)	[92.00,	130.00,	160.00]
Year of operation	04	1902	57.84	(47.1)	[24.00,	45.00,	77.00]
	05	12392	73.87	(60.3)	[29.00,	56.00,	101.00]
	06	16901	91.97	(81.6)	[33.00,	69.00,	124.00]
	07	22287	97.09	(83.3)	[36.00,	75.00,	133.00]
	08	37678	111.83	(98.2)	[44.00,	86.00,	149.00]
	09	44020	115.91	(102.8)	[45.00,	89.00,	151.00]
	10	50416	114.78	(95.7)	[48.00,	89.00,	155.00]
	11	54957	116.60	(98.2)	[50.00,	91.00,	154.00]
	12	60581	122.55	(99.0)	[55.00,	99.00,	163.00]
	13	62950	124.14	(94.3)	[58.00,	100.00,	169.00]
	14	68357	127.82	(94.1)	[63.00,	106.00,	171.00]
	15	68189	133.04	(103.8)	[64.00,	111.00,	172.00]
	16	69434	130.91	(106.2)	[65.00,	107.00,	169.00]
	17	9794	133.73	(106.7)	[67.00,	108.00,	175.00]
Centre Volume in previous 365 days	1x day	252914	99.94	(90.5)	[40.00,	75.00,	130.00]
	2x day	223425	116.00	(76.7)	[59.00,	101.00,	158.00]
	3x day	40127	142.01	(86.8)	[80.00,	127.00,	191.00]
	4x day	33584	226.83	(167.5)	[115.00,	183.00,	289.00]
	5x day	14127	198.14	(143.4)	[99.00,	172.00,	250.00]
	6x day	12630	151.66	(73.8)	[99.00,	133.00,	206.00]
	7x day	3051	156.37	(64.7)	[110.00,	142.00,	220.00]
Operation training type (Consultant / Lead surgeon / Assisted by) Number in parentheses indicates the same or different consultant	Con.(1)/Con.(1)	463705	117.46	(91.7)	[53.00,	95.00,	157.00]
	Con.(1)/Con.(1)(?)	16246	105.20	(115.9)	[40.00,	67.00,	133.00]
	Con.(1)/Con.(2)	13637	141.29	(134.4)	[49.00,	101.00,	197.00]
	Con.(1)/Jun.(1)/Con.	34006	110.47	(72.3)	[56.00,	101.00,	153.00]
	Con.(1)/Jun.(1)/Jun.(2)	52264	150.02	(139.3)	[58.00,	119.00,	191.00]
Consultant first recorded procedure post 2008 + 365days	Cons. Pre 2009	482572	126.19	(104.1)	[54.00,	101.00,	170.00]
	Cons. Post 2009	97286	90.53	(53.7)	[50.00,	81.00,	122.00]

Supplementary.Figure 1 ***Between-consultant*** *Marginal association of hip surgical volume in the preceding 365-days and hazard of revision arthroplasty adjusted with 4 different sets (M2 to M4) of confounding factors in a multi-level model (MLM).*

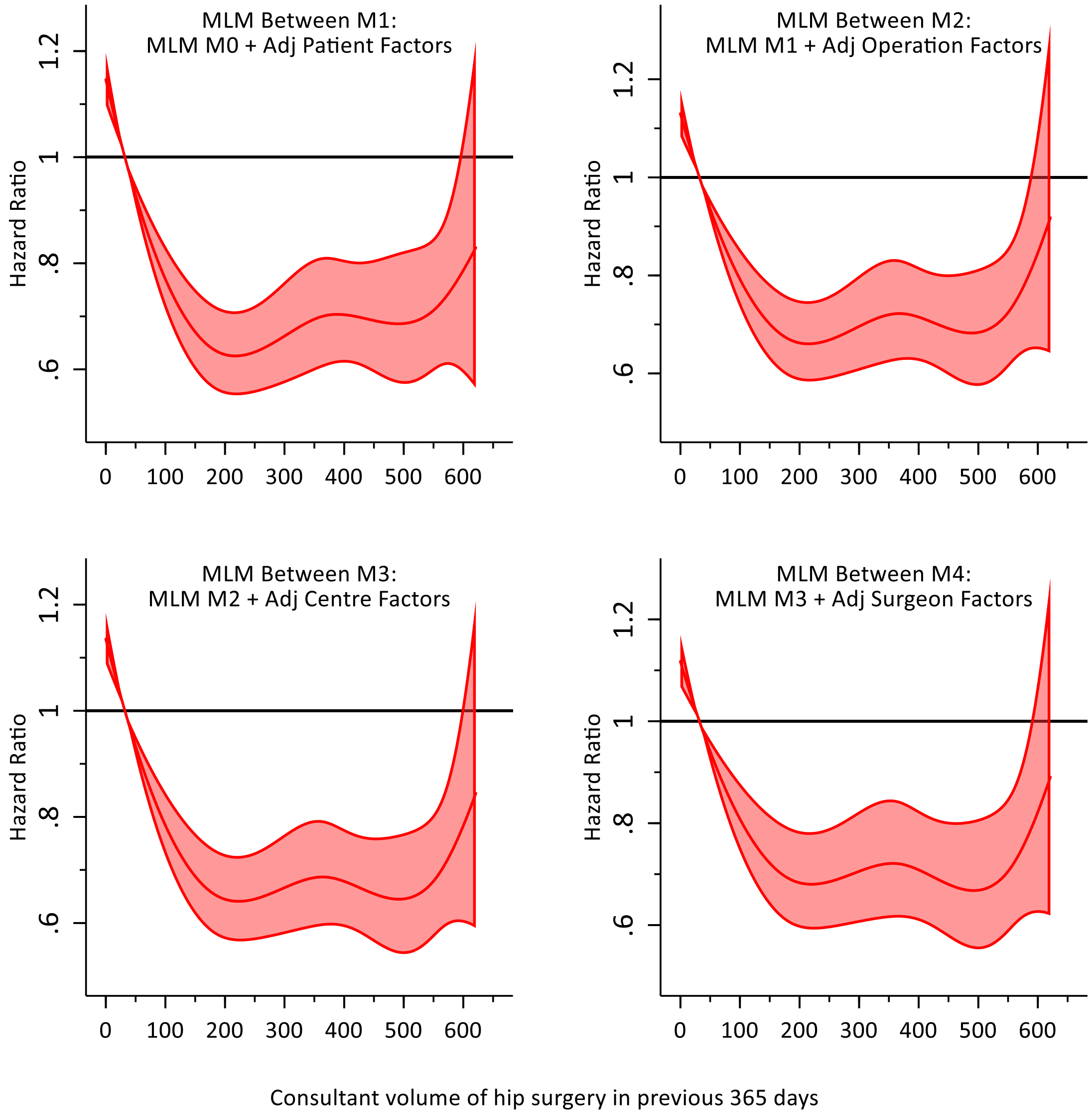

Foot Note.

Patient factors include sex, ASA grade, funder. Operation confounding factors include fixation, approach, position, anaesthetic, mechanical and chemical thromboprophylaxis, bearing, and year of operation. Centre confounding factors include hospital location and centre volume in the preceding 365-days. Surgeon confounding factors included; lead

operating surgeon, listing of a surgeon within NJR prior to 2008, the proportion of NHS cases in the preceding year, and proportion hip arthroplasty procedures undertaken in the previous year.

For peer review only

Supplementary.Figure 2 ***Within-consultant*** *Marginal association of hip surgical volume in the preceding 365-days and hazard of revision arthroplasty adjusted with 4 different sets (M2 to M4) of confounding factors in a multi-level model (MLM).*

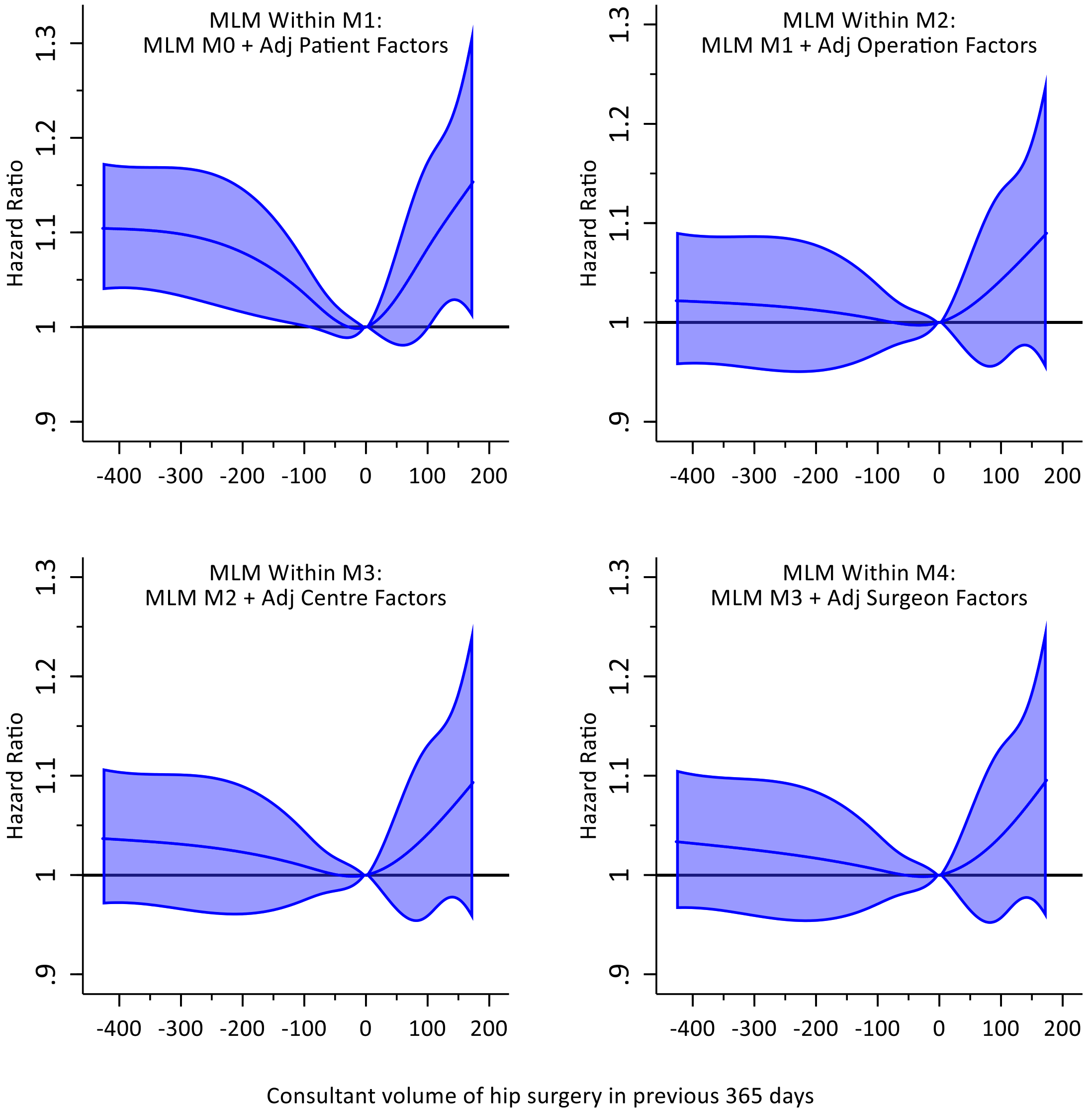

Foot Note.

Patient factors include sex, ASA grade, funder. Operation confounding factors include fixation, approach, position, anaesthetic, mechanical and chemical thromboprophylaxis, bearing, and year of operation. Centre confounding factors include hospital location and

centre volume in the preceding 365-days. Surgeon confounding factors included; lead operating surgeon, listing of a surgeon within NJR prior to 2008, the proportion of NHS cases in the preceding year, and proportion hip arthroplasty procedures undertaken in the previous year.

For peer review only

STROBE Statement—Checklist of items that should be included in reports of ***cohort studies***

	Item No	Recommendation	Page
**Title and abstract**	1	(*a*) Indicate the study's design with a commonly used term in the title or the abstract	1
		(*b*) Provide in the abstract an informative and balanced summary of what was done and what was found	2
**Introduction**			
Background/rationale	2	Explain the scientific background and rationale for the investigation being reported	4
Objectives	3	State specific objectives, including any prespecified hypotheses	5
**Methods**			
Study design	4	Present key elements of study design early in the paper	6
Setting	5	Describe the setting, locations, and relevant dates, including periods of 6recruitment, exposure, follow-up, and data collection	6
Participants	6	(*a*) Give the eligibility criteria, and the sources and methods of selection of participants. Describe methods of follow-up	6
		(*b*) For matched studies, give matching criteria and number of exposed and unexposed	
Variables	7	Clearly define all outcomes, exposures, predictors, potential confounders, and effect modifiers. Give diagnostic criteria, if applicable	7
Data sources/ measurement	8*	For each variable of interest, give sources of data and details of methods of assessment (measurement). Describe comparability of assessment methods if there is more than one group	*7/8*
Bias	9	Describe any efforts to address potential sources of bias	7/8
Study size	10	Explain how the study size was arrived at	8
Quantitative variables	11	Explain how quantitative variables were handled in the analyses. If applicable, describe which groupings were chosen and why	8 sup.t1
Statistical methods	12	(*a*) Describe all statistical methods, including those used to control for confounding	8
		(*b*) Describe any methods used to examine subgroups and interactions	n/a
		(*c*) Explain how missing data were addressed	9
		(*d*) If applicable, explain how loss to follow-up was addressed	7
		(*e*) Describe any sensitivity analyses	n/a
**Results**			
Participants	13*	(a) Report numbers of individuals at each stage of study—eg numbers potentially eligible, examined for eligibility, confirmed eligible, included in the study, completing follow-up, and analysed	Fig 1
		(b) Give reasons for non-participation at each stage	Fig 1
		(c) Consider use of a flow diagram	Fig 1
Descriptive data	14*	(a) Give characteristics of study participants (eg demographic, clinical, social) and information on exposures and potential confounders	ST2/3
		(b) Indicate number of participants with missing data for each variable of interest	Fig 1
		(c) Summarise follow-up time (eg, average and total amount)	6
Outcome data	15*	Report numbers of outcome events or summary measures over time	10
Main results	16	(*a*) Give unadjusted estimates and, if applicable, confounder-adjusted estimates and their precision (eg, 95% confidence interval). Make clear	Fig 4/5

For peer review only - http://bmjopen.bmj.com/site/about/guidelines.xhtml

		which confounders were adjusted for and why they were included	
		(*b*) Report category boundaries when continuous variables were categorized	n/a
		(*c*) If relevant, consider translating estimates of relative risk into absolute risk for a meaningful time period	n/a
Other analyses	17	Report other analyses done—eg analyses of subgroups and interactions, and sensitivity analyses	n/a
**Discussion**			
Key results	18	Summarise key results with reference to study objectives	12
Limitations	19	Discuss limitations of the study, taking into account sources of potential bias or imprecision. Discuss both direction and magnitude of any potential bias	13
Interpretation	20	Give a cautious overall interpretation of results considering objectives, limitations, multiplicity of analyses, results from similar studies, and other relevant evidence	14
Generalisability	21	Discuss the generalisability (external validity) of the study results	15
**Other information**			
Funding	22	Give the source of funding and the role of the funders for the present study and, if applicable, for the original study on which the present article is based	16

*Give information separately for exposed and unexposed groups.

Note: An Explanation and Elaboration article discusses each checklist item and gives methodological background and published examples of transparent reporting. The STROBE checklist is best used in conjunction with this article (freely available on the Web sites of PLoS Medicine at http://www.plosmedicine.org/, Annals of Internal Medicine at http://www.annals.org/, and Epidemiology at http://www.epidem.com/). Information on the STROBE Initiative is available at http://www.strobe-statement.org.

For peer review only

For peer review only - http://bmjopen.bmj.com/site/about/guidelines.xhtml

BMJ Open

The association between surgical volume and failure of primary total hip replacement in England and Wales: Findings from a prospective national joint replacement register

Journal:	*BMJ Open*
Manuscript ID	bmjopen-2019-033045.R2
Article Type:	Original research
Date Submitted by the Author:	17-Jun-2020
Complete List of Authors:	Sayers, Adrian; University of Bristol, Muscloskeletal Research Unit<br>Steele, Fiona; LSE, Department of Statistics<br>Whitehouse, Michael; University of Bristol, Musculoskeletal Research Unit<br>Price, Andrew; University of Oxford, Nuffield Department of Orthopaedics, Rheumatology and Musculoskeletal Sciences<br>Ben-Shlomo, Yoav; University of Bristol,<br>Blom, AW; University of Bristol,
**Primary Subject Heading**:	Epidemiology
Secondary Subject Heading:	Surgery, Epidemiology, Public health, Research methods
Keywords:	Total Hip Arthroplasty, Hip < ORTHOPAEDIC & TRAUMA SURGERY, volume, Revision surgery, Within Surgeon Effects, Between Surgeon effects

SCHOLARONE™
Manuscripts

I, the Submitting Author has the right to grant and does grant on behalf of all authors of the Work (as defined in the below author licence), an exclusive licence and/or a non-exclusive licence for contributions from authors who are: i) UK Crown employees; ii) where BMJ has agreed a CC-BY licence shall apply, and/or iii) in accordance with the terms applicable for US Federal Government officers or employees acting as part of their official duties; on a worldwide, perpetual, irrevocable, royalty-free basis to BMJ Publishing Group Ltd ("BMJ") its licensees and where the relevant Journal is co-owned by BMJ to the co-owners of the Journal, to publish the Work in this journal and any other BMJ products and to exploit all rights, as set out in our licence.

The Submitting Author accepts and understands that any supply made under these terms is made by BMJ to the Submitting Author unless you are acting as an employee on behalf of your employer or a postgraduate student of an affiliated institution which is paying any applicable article publishing charge ("APC") for Open Access articles. Where the Submitting Author wishes to make the Work available on an Open Access basis (and intends to pay the relevant APC), the terms of reuse of such Open Access shall be governed by a Creative Commons licence – details of these licences and which Creative Commons licence will apply to this Work are set out in our licence referred to above.

Other than as permitted in any relevant BMJ Author's Self Archiving Policies, I confirm this Work has not been accepted for publication elsewhere, is not being considered for publication elsewhere and does not duplicate material already published. I confirm all authors consent to publication of this Work and authorise the granting of this licence.

For peer review only

Full Title: The association between surgical volume and failure of primary total hip replacement in England and Wales: Findings from a prospective national joint replacement register

Short Title: Surgical volume and failure of primary total hip replacement

Adrian Sayers[1,2], Fiona Steele[3], Michael R Whitehouse[1,5], Andrew Price[4], Yoav Ben-Shlomo[2], Ashley W Blom[1,5]

1. Musculoskeletal Research Unit, Bristol Medical School, 1st Floor Learning & Research Building, Southmead Hospital, Bristol, BS10 5NB, United Kingdom
2. Population Health Sciences, Bristol Medical School, Canynge Hall, 39 Whatley Road Bristol, BS8 2PS, United Kingdom
3. Department of Statistics, London School of Economics and Political Science, London, UK
4. NIHR Biomedical Research Unit, University of Oxford, Oxford, UK
5. National Institute for Health Research Bristol Biomedical Research Centre, University Hospitals Bristol NHS Foundation Trust and University of Bristol

Corresponding Author

Adrian Sayers

Musculoskeletal Research Unit, Bristol Medical School, 1st Floor Learning & Research Building, Southmead Hospital, Bristol, BS10 5NB, United Kingdom

Email: adrian.sayers@bristol.ac.uk Tel: 0117 4147880

Word Count:2826

For peer review only
For peer review only - http://bmjopen.bmj.com/site/about/guidelines.xhtml

Abstract

Objective

Investigate the association of volume of total hip arthroplasty (THA) between consultants and within the same consultant in the previous year and the hazard of revision using multi-level survival models.

Design

Prospective cohort study using data from a national joint replacement register.

Setting

Elective total hip arthroplasty (THA) across all private and public centres in England and Wales between April 2003 and February 2017.

Participants

Patients age 50 years or more undergoing THA for osteoarthritis.

Intervention

The volume of THA conducted in the preceding 365-days to the index procedure.

Main Outcome and measure

Revision surgery (excision, addition or replacement) of a primary THA.

Results

Of the 579,858 patients undergoing primary THA (mean baseline age 69.8 years [SD 10.2]), 61.1% were female. Multi-level survival found differing results for between and within-consultant effects. There was a strong volume-revision association between consultants, with a near linear 43.3% (95% CI 29.1%, 57.4%) reduction of the risk of revision comparing consultants with volumes between 1 and 200 procedures annually. Changes in individual

For peer review only - http://bmjopen.bmj.com/site/about/guidelines.xhtml

For peer review only

surgeons (within-consultant) case volume showed no evidence of an association with revision.

Conclusion

Separation of between- and within-consultant effects of surgical volume reveals how volume contributes to the risk of revision after THA. The lack of association within-consultants suggests that individual changes to consultant volume alone will have little effect on outcomes following THA.

These novel findings provide strong evidence supporting the practice of specialisation of hip arthroplasty. It does not support the practice of low volume consultants increasing their personal volume as it is unlikely their results would improve if this is the only change. Limiting the exposure of patients to consultants with low volumes of THA and greater utilization of centres with higher volume surgeons with better outcomes may be beneficial to patients.

Strengths and limitations of this study

- This is the largest study in the world to explore the association between surgical volume and outcomes in total hip arthroplasty
- We uniquely calculate a time-varying exposure of surgical volume.
- We differentiate between- and within- consultant effects using a multi-level Weibull survival model.
- The effect of volume is modelled continuously using restricted cubic splines.
- We are unable to affirm causality due to the observational nature of the data

Funding Statement

AS is funded by an MRC Strategic Skills Fellowship MR/L01226X/1

For peer review only - http://bmjopen.bmj.com/site/about/guidelines.xhtml

This study was supported by the NIHR Biomedical Research Centre at University Hospitals Bristol NHS Foundation Trust and the University of Bristol. The views expressed in this publication are those of the author(s) and not necessarily those of the NHS, the National Institute for Health Research or the Department of Health and Social Care.

Competing interests statement

We declare no competing interests

For peer review only

Keywords

Total Hip Arthroplasty, Volume, Revision, Within surgeon effects, Between surgeon effects.

Introduction

Centralisation and specialisation in medical care are advocated to optimise a theorised volume-outcome relationship. In arthroplasty, the volume-outcome relationship has been investigated with respect to outcomes including surgical revision,[1-6] mortality,[1 6-9] patient reported outcome measures (PROMS),[10] and complications[6 11-14] where volume is measured either by surgeon[1-6] or hospital annual volume.[15] Given the technical requirements of arthroplasty, a strong argument for specialisation based on surgical volume and the risk of revision arthroplasty would exist if volume was causally related to outcome. The evidence to support this assertion is surprisingly sparse and has methodological limitations.[1-6] The principal limitation is the failure to distinguish between- and within-consultant effects.

Differentiating between- and within-consultant effects is crucial to interpreting the data. A between-consultant effect is essentially a cross-sectional analysis that compares the performance of one consultant against another and is highly likely to be confounded by centre level effects.[16] A within-consultant effect is based on individual time series data and compares changes of volume across time within the same consultant. Correspondingly, within effects can be interpreted more strongly, as the effect of changing a consultant's personal volume, assuming centre-level factors remain relatively constant over the short term analysis period.

The concept of between- and within-effects is well known in epidemiology, and the ecological fallacy is one example. For example, in a standard (single-level) regression analysis, we may observe a positive association between red meat consumption and life expectancy across several countries with differing levels of development. A between- and within-decomposition would reveal a positive between-country correlation, which is explained by the level of development, and a negative within-country association i.e. individuals within a country who eat more red meat have a lower life expectancy. The

For peer review only - http://bmjopen.bmj.com/site/about/guidelines.xhtml

decomposition of the volume effect into a between- and within-effect is similar i.e. the between-consultant effect is explained by factors, other than volume, which are intrinsic to those consultants and the hospital where they are based ("centre effects"), whereas the within-consultant effect represents the consequence of consultants individually changing their personal volume assuming that other factors remain constant.

In order to facilitate a between- and within-effects analysis, consultant volume needs to be assessed continuously across time. Allowing volume to vary over time is computationally intensive, but responds to variation in demand and capacity to deliver arthroplasty, specialisation or diversification of professional practice, and is in contrast to previous approaches.[1 4] Additionally, as consultant volume changes, dichotomising the data using arbitrary thresholds, e.g. 12 operations per year,[3] is difficult to justify as a consultant's volume will vary over the time they are observed. Furthermore, the interpretation of the results is less likely to be distorted by the arbitrary placement of the threshold.[17 18]

The aim of this research is to investigate the between- and within-consultant (surgeon) effect of the volume of primary total hip arthroplasty (THA) and the risk of subsequent revision.

For peer review only - http://bmjopen.bmj.com/site/about/guidelines.xhtml

Methods

Using data from the National Joint Registry of England, Wales, Northern Ireland and the Isle of Man (NJR), we investigated the association between consultant surgical volume in the year (365-days) prior to the index operation of interest, and the risk of revision in patients undergoing elective THA between 1st April 2003 and 22nd February 2017.

Data source

The NJR commenced data collection in April 2003; at inception, it was mandatory for all THA conducted in the private sector to be entered into the NJR, and from 2011 all THA procedures in the public and private sector were required to be entered into the NJR. A recent national audit of data entered into the NJR between 2014 and 2015 estimated data capture of 95% for primary THA and 91% for revision THA.

Ethical Approval

Pseudo anonymised analysis of NJR data is considered as secondary use of clinical registry data, under HRA guidance this does not require formal ethical approval. The full NJR privacy notice can be found online (http://www.njrcentre.org.uk/njrcentre/About-the-NJR/Privacy-Notice-GDPR).

Patient and public involvement

Patient representatives sit on the committee structure of the National Joint Registry. The research priorities of the National Joint Registry are identified by this committee structure and approved by the patient representatives. Patients were not involved in the setting of the research question or the outcome measures, nor were they involved in designing or implementing this work or interpretation of the results. We are unable to disseminate results of this study directly to study participants due to the anonymous nature of the data. We plan to disseminate our findings to the National Joint Registry, via their communications team, to

For peer review only - http://bmjopen.bmj.com/site/about/guidelines.xhtml

relevant individuals who determine the provision of joint replacement, and to the general population through the local and national press.

Inclusion/exclusion criteria

All consenting patients undergoing THA were eligible for inclusion in the analysis. Patients were included if their patient history was unique and consistent, i.e. contained no duplicates, ipsilateral revision prior to the primary, or currently held in query by the submitting unit. Due to the requirement of reliable date information, patients who were indicated to have died prior to undergoing a procedure, were more than 110 years of age, had undergone a procedure prior to their date of birth, or received a procedure prior to 2003 were excluded. Only primary THA, where the sole indication for operation was osteoarthritis (OA) with unique prosthesis combinations, were included. All metal-on-metal bearing combinations were excluded from the analysis due to the known exceptionally high failure rate in this group.[19 20] Consultants with less than 365-days of data were excluded as were patients who were less than 50 years of age at the date of the index THA, because these cases are highly likely to be due to secondary OA. See Figure 1 and Figure 2 for a detailed breakdown of inclusion criteria.

Primary outcome

The primary outcome of interest is all cause revision after a primary THA. Revision arthroplasty was identified by the inclusion of a revision specific data upload after a primary ipsilateral THA. We note that it is not always the primary surgeon that performs the revision.

Censoring

Patients were censored following death. Death status was established by linking patients to the NHS Personal Demographic tracing service.

For peer review only - http://bmjopen.bmj.com/site/about/guidelines.xhtml

Primary exposure

The primary exposure of interest in this study was the consultant surgical volume of any THA recorded in the NJR in the preceding 365-days prior to the index procedure in consenting patients, and prior to the application of inclusion and exclusion criteria, see Figure 1. We choose a 365 day period as this represents one calendar year, and this effectively integrates out seasonal variation from the volume definition,

Confounding factors

Confounding factors were thematically organised into 5 groups:

1) Patient factors: Age, sex, American Society of Anesthesiologists (ASA) grade, and operation funder.
2) Operation factors: THA fixation, approach, patient position during arthroplasty, anaesthetic type, thromboprophylaxis regime, bearing, and year of primary THA.
3) Centre factors included the setting of the treatment episode (i.e. private or NHS hospital) and surgical centre volume.
4) Consultant based factors included the training status of the primary surgeon performing the operation, whether the responsible consultant was listed in the NJR after to 2008 (i.e. a newly qualified surgeon), the proportion of THA undertaken in the NHS in the preceding 365-days by the consultant (i.e. a public or private hospital surgeon), the proportion of THA procedures undertaken in the previous year compared to all joints recorded by the NJR (i.e. a specialist hip surgeon or a general arthroplasty surgeon).
5) Deprivation factors were based on the English and Welsh indices of multiple deprivation, an area based index of patient socioeconomic status. See Supplementary.Table 1.

For peer review only - http://bmjopen.bmj.com/site/about/guidelines.xhtml

Statistical analyses

Means, standard-deviations and interquartile points were used to describe continuous variables. Frequencies and percentages were used to describe categorical variables. The association between confounding factors and consultant volume was explored by comparing summary statistics between levels of each factor.

Graphical methods including frequency distributions, and empirical cumulative distributions were used to describe the relative frequency and centiles of the volume distributions. The empirical cumulative distribution allows centiles of the distribution to be quickly identified.

The associations between consultant volume in the preceding 365-days of the index procedure and all-cause revision were explored using multi-level parametric (Weibull) survival models.

Between- and within-effects are decomposed using a process known as group mean centring.[21 22] Group mean centring is the process of creating two new variables from the primary exposure. The first variable is the consultant specific mean volume, and the second variable is the deviation of a consultant's volume for a given procedure from their personal average (i.e. consultant-mean centred volume). The between-effect is estimated as the coefficient of a consultant's mean volume, whereas the within-effect is the coefficient of the consultant's deviation from their average.

Continuous variables were modelled used orthogonalized Restricted Cubic Splines (RCS), this also included the volume effect. Each RCS is centred at meaningful value, these values are listed in supplementary material, mean between consultant volume is centred at 32 procedures annually, whereas within consultant volume is naturally centred at zero. We iteratively varied the number of knot points and placement strategies, in unadjusted models to

For peer review only

optimize fit. The most parsimonious specification of RCS was selected using Akaike Information Criterion (AIC).[23]

Confounding adjustment was conducted incrementally introducing patient, operation, centre, surgeon, and finally, deprivation confounding variable groups. The effect of confounding adjustment on the primary exposure of interest was explored and presented at each stage of the model building process allowing the effect of adjustment to be clearly illustrated. All modelling was conducted using the `mestreg` package in Stata 15.1.[24]. The specification of the model is described in more detail in the supplementary material.

Missing data

Given the large data set and small fraction of incomplete cases among the observed (FICO) (89% of all eligible records were included in the analysis), any improvement in efficiency from multiple imputation is likely to be negligible, and a complete-cases analysis would provide unbiased results.[25 26] Therefore, we have assumed the reason for missingness is independent of both the outcome and primary exposure of interest.

For peer review only

Results

Between 1st April 2003 and 22nd February 2017, 1,008,619 primary and revision THA procedures were entered in the NJR. After application of inclusion and exclusion criteria, 579,858 primary THA replacement procedures were available for analysis, (Figure 1), including 9238 revised procedures, with 2.5 million years of observation time, with a maximum length of follow up of 13.14 years. Patients were predominately female (61.1%), ASA II (70.4%), treated in public (NHS) hospitals (84.1%), and had a mean age 69.8 years (SD 10.2). See Supplementary.Table 2 and Supplementary.Table 3 for full descriptive statistics of the population of patients used in the analysis.

Mean between-consultant volume in the previous year was positively skewed, with only a minority (>97.5% of the empirical cumulative distribution) of consultants performing in-excess of 200 procedures a year, (Figure 3). The median number of procedures conducted across all consultants, for the whole analysis period, in the year prior to an index procedure was 95 (IQR 52, 158) with substantial variability between consultants, (Figure 4). Mean within-consultant volume, for the most part, was symmetrically distributed. There was substantial variability of volume within consultants (median within-consultant SD is 23 (IQR 14.5, 36.5)), (Figure 3), and consultants with larger mean volumes tended to have greater variability, (Figure 4). We also note that the centre IQR of centre volume, is much less variable than consultant volume, but is more negatively skewed at centre level, (Figure 4).

Summary statistics of consultant volume by confounding factors at the index procedure are listed in Supplementary.Table 4. Higher volume consultants were observed to treat patients who were younger, with lower ASA grade, who privately funded their THA, and received a uncemented or hybrid prostheses more frequently. They were also more likely to use a posterior approach, place patients in the lateral position, work in the private sector,

For peer review only - http://bmjopen.bmj.com/site/about/guidelines.xhtml

predominately perform hip arthroplasty, and to treat patients from more deprived areas, (Supplementary.Table 4).

The effect of confounding adjustment on the volume association was explored using complete cases in estimation of the multi-level Weibull model. The marginal between- and within-consultant effects of volume in the preceding year on hazard of revision and 95% CI are presented in Figure 5, Supplementary Figure 1 and Figure 6, Supplementary Figure 2 respectively.

A near linear 48.8% (95% CI:36.3,61.4) reduction in hazard ratio was observed in crude models for volumes between 1 and 200 procedures annually i.e. from 1.13 (95% CI 1.08, 1.18) to 0.64 (95% CI 0.56, 0.72), with a flattening of risk beyond this, noting that only ~2.5% of consultant recorded more than 200 procedures annually. Despite extensive confounding adjustment, the marginal association of the between-consultant volume effect remained remarkably consistent across the range of volumes observed. In our fully adjusted (deprivation) model, a near linear 43.3% (95% CI 29.1, 57.4) reduction in hazard ratio was observed. The marginal association of the within-consultant volume effect and revision was similarly consistent and showed no evidence (Figure 6) of an association with revision if consultants increased or decreased their volume by ± 50 procedures per year (HR ~1 and 95% CI includes zero).

For peer review only - http://bmjopen.bmj.com/site/about/guidelines.xhtml

Discussion

We provide novel insights into the volume-outcome relationship of 579,858 elective THA patients using a between- and within-decomposition to analyse the association of consultant volumes on revision. Between different consultants, the volume of arthroplasty in the previous year is associated with a near linear 49% and 43% reduction in hazard ratio, between 1 and 200 procedures with revision THA in crude and fully adjusted models respectively. Within the same consultant we demonstrate that there is no evidence of an association between volume of THA in the previous year and risk of revision.

Uniquely, we use a time-varying volume specification that facilitates the decomposition of between- and within-consultant effects. We suggest the within-consultant effect is much closer to the causal interpretation desired by many policymakers, and failure of research to recognise the difference amongst between- and within-effects may lead to erroneous policy decisions and unintended consequences.

We demonstrate that optimal between-consultant results are reached when the consultant volumes in the previous year are approximately 200 procedures. We suggest these factors are not causally related to volume, but rather due to unmeasured surgeon, patient and/or centre factors. There is no evidence to suggest that consultants should change their personal volume in the hope of improving their outcomes or that there is an arbitrary threshold where the outcome of results become good. It is important to add that these volume changes are for experienced consultants who have already passed early improvements that one might observe at the early trainee level due to practice. Furthermore, their low volume for hip replacement may reflect high volume experience for other procedures thereby ensuring manual dexterity albeit for a different operation.

For peer review only - http://bmjopen.bmj.com/site/about/guidelines.xhtml

Whilst the results appear contradictory compared to previous research i.e. no threshold volume effect, the differences may be explained by the method of analysis, i.e. single-level models vs. multi-level model, and interpretation of results, i.e. separation of between- and within-consultant volume effects. Previous analyses are single-level analyses assume that all procedures are independent of one another, so that a low-volume consultant would achieve the results of a high-volume consultant if they could instantly increase their personal volume. The interpretation of a single-level model is similar to that of the between-consultant interpretation. Whilst this may be an attractive interpretation for policymakers, it fails to recognise the complexity of the data and processes observed, and that there are many factors, intrinsic to each consultant and the centre or centres in which they work, that predispose them to be either low- or high-volume surgeons e.g. fellowship trained, threshold for revision, or unit organisation.

This study has a number of strengths and limitations. Strengths include: 1) our unique decomposition of between- and within-consultant which we believe provides a more actionable interpretation for policy makers. 2) Time-varying consultant volume, which is independent of the index procedure, and allows for stronger inferences. 3) OA was the only indication for arthroplasty, which we believe represents a "best case scenario", and the volume effect will only be attenuated by the inclusion of other diagnoses. 4) We demonstrate the use of RCS to model volume effects, which ensures flexibility and a smooth continuous function, emphasising the lack of any threshold in the volume effect. 5) The study is significantly, 10 times larger, than any other published study on this topic,[1-6] with a maximal follow-up period of more than 13 years. 6) we have conducted extensive case-mix adjustment and illustrate that both between- and within-effects are insensitive to our measured confounding factors.

For peer review only - http://bmjopen.bmj.com/site/about/guidelines.xhtml

Despite the many strengths, this study has a number of limitations. 1) The analysis and decomposition of between- and within-consultant effects is more complex than traditional analyses assume, and requires careful interpretation. 2) Despite the independent nature of the volume of arthroplasty calculation prior to the index procedure, we still see anticipated associations, i.e. younger patients, patients with lower ASA scores, patients receiving uncemented implants and patients operated on in the private sector all tend to be treated by higher volume consultants. Consultants specialising in THA and working principally within the private sector accrue higher volumes. These features suggest a strong propensity in practice to treat similar patients and raises the possibility of lagged correlation in unmeasured confounders.[27] 3) The use of a single indication for arthroplasty, namely OA, may limit the generalisability of results particularly in regard to Black or Asian ethnicities where OA is not as dominant an indication for THA.[28] 4) The use of RCS requires extensive sensitivity analyses to ensure knot points are placed optimally, and that results are not sensitive to knot placement. 5) Calculation of the time-varying volume specification of THA in the previous year is computationally intensive and requires significant parallelisation before analyses can be started. 6) Our covariates are unlikely to capture important centre differences in staffing, organization and policy in the management of THAs. 7) Volume does not fully encapsulate experience or expertise that may have been acquired prior to the inception of the register for example during fellowships or from working in other healthcare systems, or the potential persistent effect of learnt experience. 8) Revision surgery is only one possible endpoint and other endpoints including Patient Reported Outcome Measures (PROMs) or other adverse events may also be important to patients. 9) We only considered a multi-level Weibull model to model the baseline hazard; other more flexible functional forms may accommodate the baseline hazard more appropriately, however model convergence is always a challenge.

For peer review only - http://bmjopen.bmj.com/site/about/guidelines.xhtml

We suggest the within-consultant effect from the multi-level regression is much closer to the causal interpretation required by consultants, patients, and policymakers i.e. what is the effect of changes in personal volume on the hazard of revision THA? This is not to say the between-effect is not of interest to policy makers, but to say that the between-effect suggests that there are intrinsic differences between high and low volume consultants i.e. expertise, where higher volume consultants tend to have better outcomes, but these differences cannot be attributed to volume per se. We suggest our analyses illustrate "State vs Trait" behaviour. Where between-consultant association illustrates the "traits" of surgeons, and within-consultant associations illustrate their "state". This is to say traits of experienced high volume surgeons with good outcomes are unaffected by changes to their personal volume. Conversely, experienced low volume arthroplasty surgeons who transiently increase their personal volume do not improve their outcomes.

Conclusion

In summary, using data from the largest arthroplasty register in the world,[29] we have demonstrated that there is no within-consultant association between surgical volume in the previous year and the risk of revision in patients undergoing primary THA for OA. Whereas there is strong evidence to suggest higher volume consultants tend to have better outcomes for reasons that are unlikely to be due to the volume of arthroplasty in the previous year per se.

The results from this study have profound implications for quality improvement within healthcare. Encouraging consultants to undertake a minimum number of procedures under the guise of raising standards could be counterproductive and may only serve to expose patients to increased risk of revision by low or previously low volume consultants. Centralisation and specialisation of THA in consultants who for reasons, not including volume, can undertake a greater number of procedures is likely to benefit patients and reduce the revision burden

For peer review only - http://bmjopen.bmj.com/site/about/guidelines.xhtml

overall. Encouraging or training low volume consultants to use prosthesis combinations with better outcomes may be more effective methods of improving outcomes for patients.

Importantly, the combined use of an independent time-varying consultant volume with multi-level (between- and within-) regression modelling allows results to be interpreted more clearly than a cross-sectional analysis of conventional observational data. Our work highlights the importance of appropriate methodology, and whilst this may fall short of being definitively causal, results from a randomised experiment are unlikely to be feasible in this population. We believe this represents the best available evidence to guide policy formulation.

For peer review only

List of abbreviations

THA: Total Hip Arthroplasty

PROMS: patient reported outcome measures

NJR: National Joint Registry of England, Wales, Northern Ireland and the Isle of Man

OA: Osteoarthritis

ASA: American Society of Anesthesiologists

NHS: National Health Service

RCS: Restricted Cubic Splines

AIC: Akaike Information Criterion

FICO: Fraction of incomplete cases among the observed

IQR: Interquartile Range

Author Contributions

AS, FS, YBS, AJP, MRW, and AWB were responsible for the study design, AS conducted the data analysis. AS, FS, YBS, AJP, MRW, and AWB were responsible for interpreting the data. AS, FS, YBS, AJP, MRW, and AWB prepared and edited and approved the final manuscript.

For peer review only - http://bmjopen.bmj.com/site/about/guidelines.xhtml

Role of the funding source

The Medical Research Council or National Institute of Health Research had no role in the design and conduct of the study; collection, management, analysis, and interpretation of the data; preparation, review, or approval of the manuscript; and decision to submit the manuscript for publication

Availability of data and material

Access to the data can be made via research requests to the National Joint Registry of England, Wales, Northern Ireland and the Isle of Man. Full details can be found at http://www.njrcentre.org.uk/njrcentre/Research/Research-requests

Acknowledgements

We thank the patients and staff of all the hospitals who have contributed data to the National Joint Registry. We are grateful to the Healthcare Quality Improvement Partnership, the National Joint Registry Steering Committee, and staff at the National Joint Registry for facilitating this work. The views expressed represent those of the authors and do not necessarily reflect those of the National Joint Registry Steering Committee or Healthcare Quality Improvement Partnership, who do not vouch for how the information is presented.

For peer review only

For peer review only - http://bmjopen.bmj.com/site/about/guidelines.xhtml

Figure Titles

Figure 1: Flow chart of participant inclusion and exclusion criteria

Figure 2: Flow chart of participant missing data

Figure 3: Empirical cumulative distribution and frequency distribution of (Between) mean consultant volume and (Within) individual centred volume of hip arthroplasty in the previous 365-days.

Foot Note

Grey horizontal hashed lines indicate the 2.5th, 25th, 50th, 75th, and 97.5th centile of the distribution, vertical hashed lines indicate mean and centred consultant volume at 2.5th, 25th, 50th, 75th, and 97.5th centiles respectively.

Figure 4: Mean, interquartile range, and 95th centile range of consultant and centre volume of hip arthroplasty in the previous 365-days recorded in the NJR by individual consultant, and individual unit respectively.

*Figure 5: **Between-consultant** Marginal association of hip surgical volume in the preceding 365-days and hazard of revision arthroplasty unadjusted (M1) and adjusted (M5) for confounding factors in a multi-level model (MLM).*

Foot Note.

Patient factors include sex, ASA grade, funder. Operation confounding factors include fixation, approach, position, anaesthetic, mechanical and chemical thromboprophylaxis, bearing, and year of operation. Centre confounding factors include hospital location and centre volume in the preceding 365-days. Surgeon confounding factors included; lead operating surgeon, listing of a surgeon within NJR prior to 2008, the proportion of NHS

For peer review only

cases in the preceding year, and proportion hip arthroplasty procedures undertaken in the previous year.

Figure 6: ***Within-consultant*** *Marginal association of hip surgical volume in the preceding 365-days and hazard of revision arthroplasty unadjusted (M1) and adjusted (M5) for confounding factors in a multi-level model (MLM).*

Foot Note.

Patient factors include sex, ASA grade, funder. Operation confounding factors include fixation, approach, position, anaesthetic, mechanical and chemical thromboprophylaxis, bearing, and year of operation. Centre confounding factors include hospital location and centre volume in the preceding 365-days. Surgeon confounding factors included; lead operating surgeon, listing of a surgeon within NJR prior to 2008, the proportion of NHS cases in the preceding year, and proportion hip arthroplasty procedures undertaken in the previous year.

For peer review only

For peer review only - http://bmjopen.bmj.com/site/about/guidelines.xhtml

1. Kreder HJ, Deyo RA, Koepsell T, et al. Relationship between the volume of total hip replacements performed by providers and the rates of postoperative complications in the state of Washington. *J Bone Joint Surg Am* 1997;79(4):485-94.
2. Kreder HJ, Williams JI, Jaglal S, et al. Are complication rates for elective primary total hip arthroplasty in Ontario related to surgeon and hospital volumes? A preliminary investigation. *Can J Surg* 1998;41(6):431-7.
3. Losina E, Barrett J, Mahomed NN, et al. Early failures of total hip replacement: effect of surgeon volume. *Arthritis Rheum* 2004;50(4):1338-43. doi: https://dx.doi.org/10.1002/art.20148
4. Manley M, Ong K, Lau E, et al. Effect of volume on total hip arthroplasty revision rates in the United States Medicare population. *J Bone Joint Surg Am* 2008;90(11):2446-51. doi: https://dx.doi.org/10.2106/JBJS.G.01300
5. Khatod M, Cafri G, Namba RS, et al. Risk factors for total hip arthroplasty aseptic revision. *J Arthroplasty* 2014;29(7):1412-7. doi: https://dx.doi.org/10.1016/j.arth.2014.01.023
6. Ravi B, Jenkinson R, Austin PC, et al. Relation between surgeon volume and risk of complications after total hip arthroplasty: propensity score matched cohort study. *Bmj* 2014;348:g3284. doi: https://dx.doi.org/10.1136/bmj.g3284
7. Katz JN, Losina E, Barrett J, et al. Association between hospital and surgeon procedure volume and outcomes of total hip replacement in the United States medicare population. *J Bone Joint Surg Am* 2001;83-A(11):1622-9.
8. Lavernia CJ. Hemiarthroplasty in hip fracture care: effects of surgical volume on short-term outcome. *J Arthroplasty* 1998;13(7):774-8.
9. Lavernia CJ, Guzman JF. Relationship of surgical volume to short-term mortality, morbidity, and hospital charges in arthroplasty. *J Arthroplasty* 1995;10(2):133-40.
10. Katz JN, Phillips CB, Baron JA, et al. Association of hospital and surgeon volume of total hip replacement with functional status and satisfaction three years following surgery. *Arthritis Rheum* 2003;48(2):560-8. doi: https://dx.doi.org/10.1002/art.10754
11. Solomon DH, Losina E, Baron JA, et al. Contribution of hospital characteristics to the volume-outcome relationship: dislocation and infection following total hip replacement surgery. *Arthritis Rheum* 2002;46(9):2436-44. doi: https://dx.doi.org/10.1002/art.10478
12. Battaglia TC, Mulhall KJ, Brown TE, et al. Increased surgical volume is associated with lower THA dislocation rates. *Clin Orthop* 2006;447:28-33. doi: https://dx.doi.org/10.1097/01.blo.0000218743.99741.f0
13. Khatod M, Barber T, Paxton E, et al. An analysis of the risk of hip dislocation with a contemporary total joint registry. *Clin Orthop* 2006;447:19-23. doi: https://dx.doi.org/10.1097/01.blo.0000218752.22613.78
14. Yasunaga H, Tsuchiya K, Matsuyama Y, et al. High-volume surgeons in regard to reductions in operating time, blood loss, and postoperative complications for total hip arthroplasty. *J Orthop Sci* 2009;14(1):3-9. doi: https://dx.doi.org/10.1007/s00776-008-1289-4
15. Judge A, Chard J, Learmonth I, et al. The effects of surgical volumes and training centre status on outcomes following total joint replacement: analysis of the Hospital Episode Statistics for England. *J Public Health (Oxf)* 2006;28(2):116-24. doi: https://dx.doi.org/10.1093/pubmed/fdl003

For peer review only - http://bmjopen.bmj.com/site/about/guidelines.xhtml

16. Hodsman A, Ben-Shlomo Y, Roderick P, et al. The 'Centre Effect' in Nephrology: What Do Differences between Nephrology Centres Tell Us about Clinical Performance in Patient Management? *Nephron Clin Pract* 2011;119(1):C10-C17. doi: 10.1159/000321378
17. Orsini N, Greenland S. A procedure to tabulate and plot results after flexible modeling of a quantitative covariate. *Stata J* 2011;11(1):1-29.
18. Greenland S. Avoiding power loss associated with categorization and ordinal scores in dose-response and trend analysis. *Epidemiology* 1995;6(4):450-4. [published Online First: 1995/07/01]
19. Smith AJ, Dieppe P, Howard PW, et al. Failure rates of metal-on-metal hip resurfacings: analysis of data from the National Joint Registry for England and Wales. *Lancet* 2012;380(9855):1759-66. doi: 10.1016/S0140-6736(12)60989-1 [published Online First: 2012/10/06]
20. Smith AJ, Dieppe P, Vernon K, et al. Failure rates of stemmed metal-on-metal hip replacements: analysis of data from the National Joint Registry of England and Wales. *Lancet* 2012;379(9822):1199-204. doi: 10.1016/S0140-6736(12)60353-5 [published Online First: 2012/03/16]
21. Neuhaus JM, McCulloch CE. Separating between- and within-cluster covariate effects by using conditional and partitioning methods. *J Roy Stat Soc B* 2006;68:859-72. doi: DOI 10.1111/j.1467-9868.2006.00570.x
22. Skrondal A, Rabe-Hesketh S. Generalized Latent Variable Modelling: Multilevel, Longitudinal , and Structural Equation Models. Boca Raton: Chapman & Hall/CRC 2004.
23. Akaike H. Citation Classic - a New Look at the Statistical-Model Identification. *Cc/Eng Tech Appl Sci* 1981(51):22-22.
24. Stata Statistical Software: Release 14 [program]. College Station, TX: StataCorp LP, 2015.
25. White IR, Carlin JB. Bias and efficiency of multiple imputation compared with complete-case analysis for missing covariate values. *Stat Med* 2010;29(28):2920-31. doi: 10.1002/sim.3944 [published Online First: 2010/09/16]
26. Schafer JL, Graham JW. Missing data: Our view of the state of the art. *Psychol Methods* 2002;7(2):147-77. doi: 10.1037//1082-989x.7.2.147
27. Bellemare MF, Masaki T, Pepinsky TB. Lagged Explanatory Variables and the Estimation of Causal Effect. *J Polit* 2017;79(3):949-63. doi: 10.1086/690946
28. Smith MC, Ben-Shlomo Y, Dieppe P, et al. Rates of hip and knee joint replacement amongst different ethnic groups in England: an analysis of National Joint Registry data. *Osteoarthritis Cartilage* 2017;25(4):448-54. doi: 10.1016/j.joca.2016.12.030 [published Online First: 2017/02/06]
29. Malchau H, Garellick G, Berry D, et al. Arthroplasty implant registries over the past five decades: Development, current, and future impact. *J Orthop Res* 2018;36(9):2319-30. doi: 10.1002/jor.24014 [published Online First: 2018/04/18]

For peer review only - http://bmjopen.bmj.com/site/about/guidelines.xhtml

Figure 1. Flow chart of participant inclusion and exclusion criteria

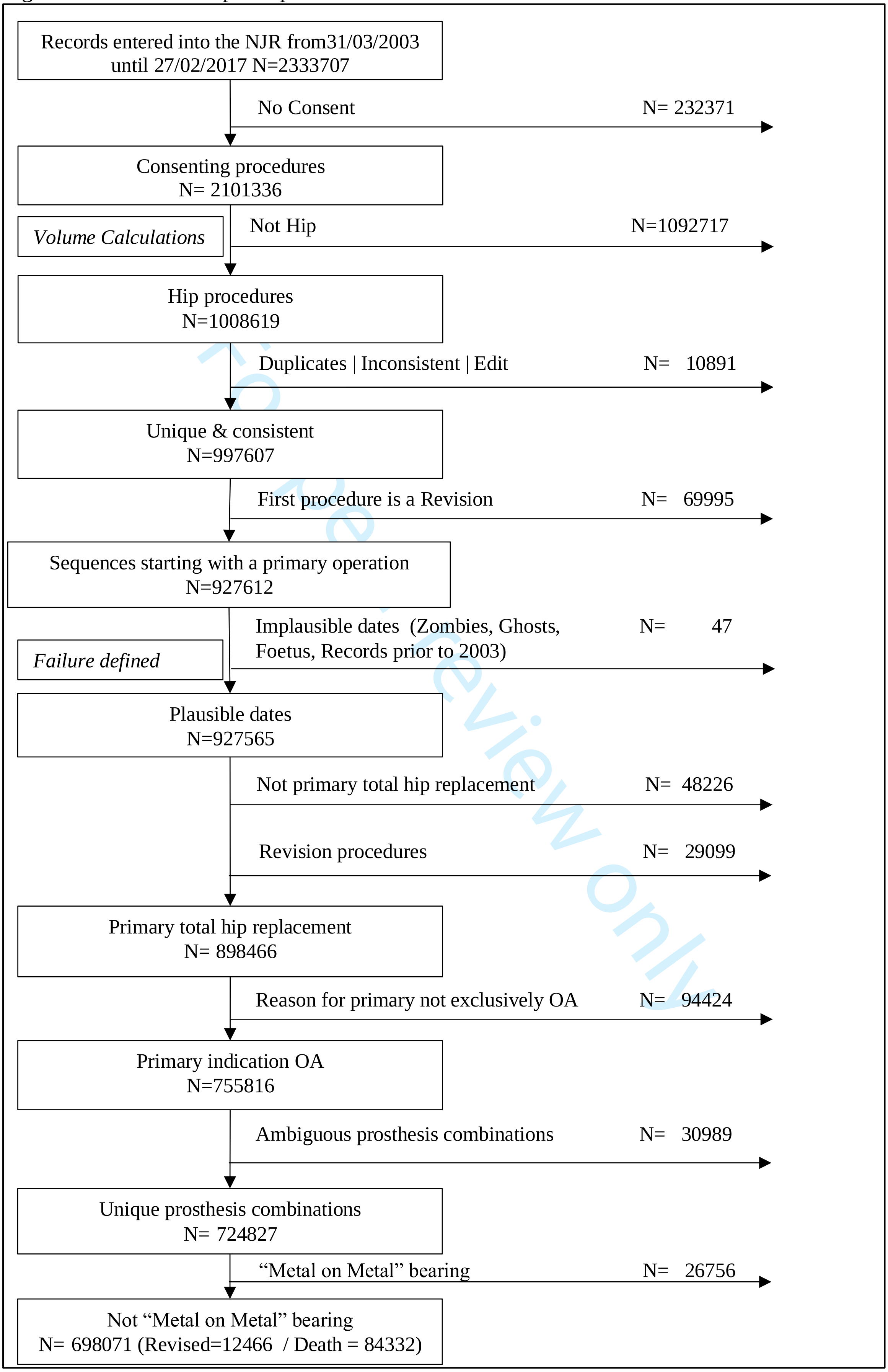

For peer review only - http://bmjopen.bmj.com/site/about/guidelines.xhtml

Figure 2. Flow chart of participant missing data

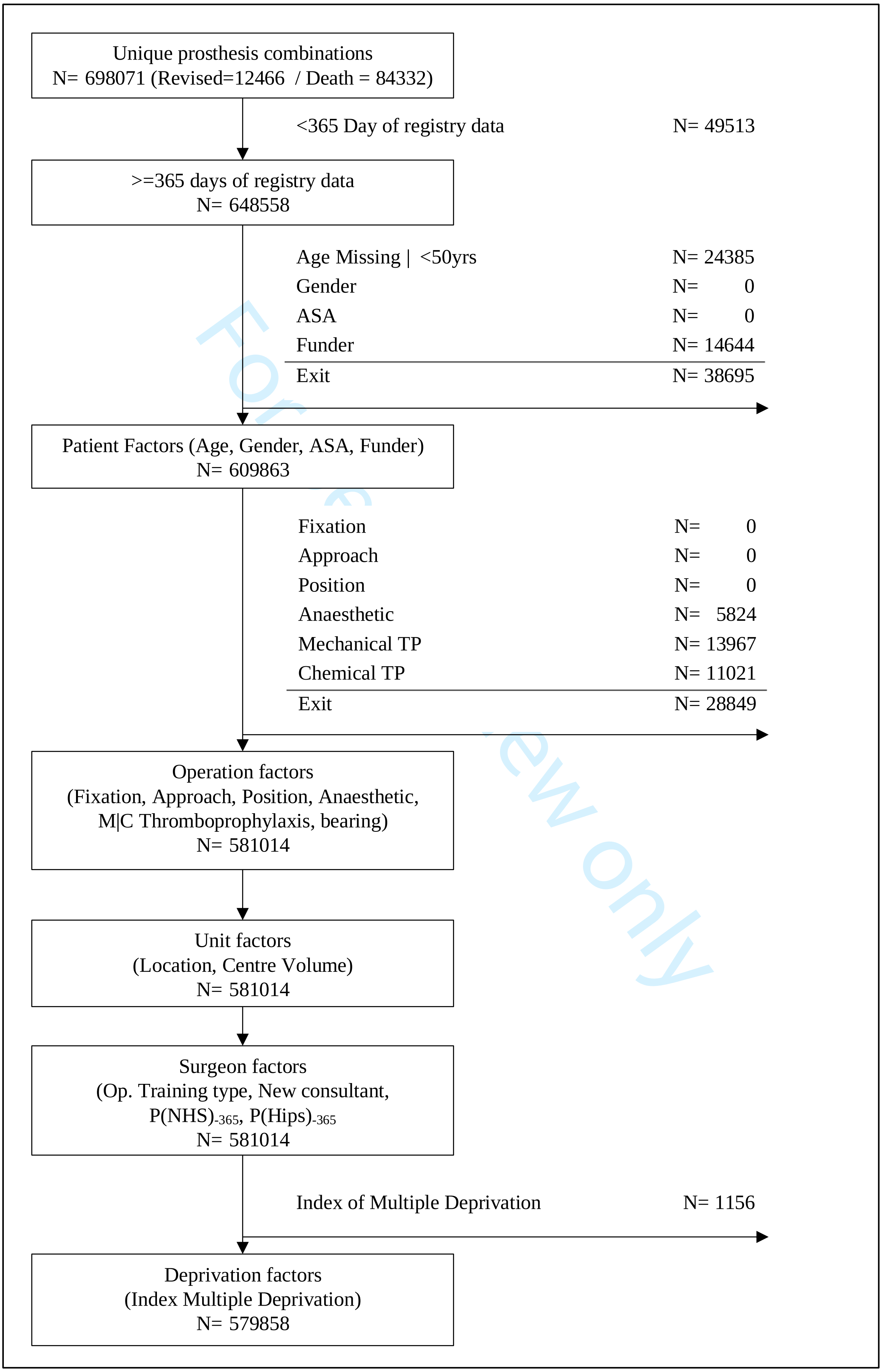

For peer review only

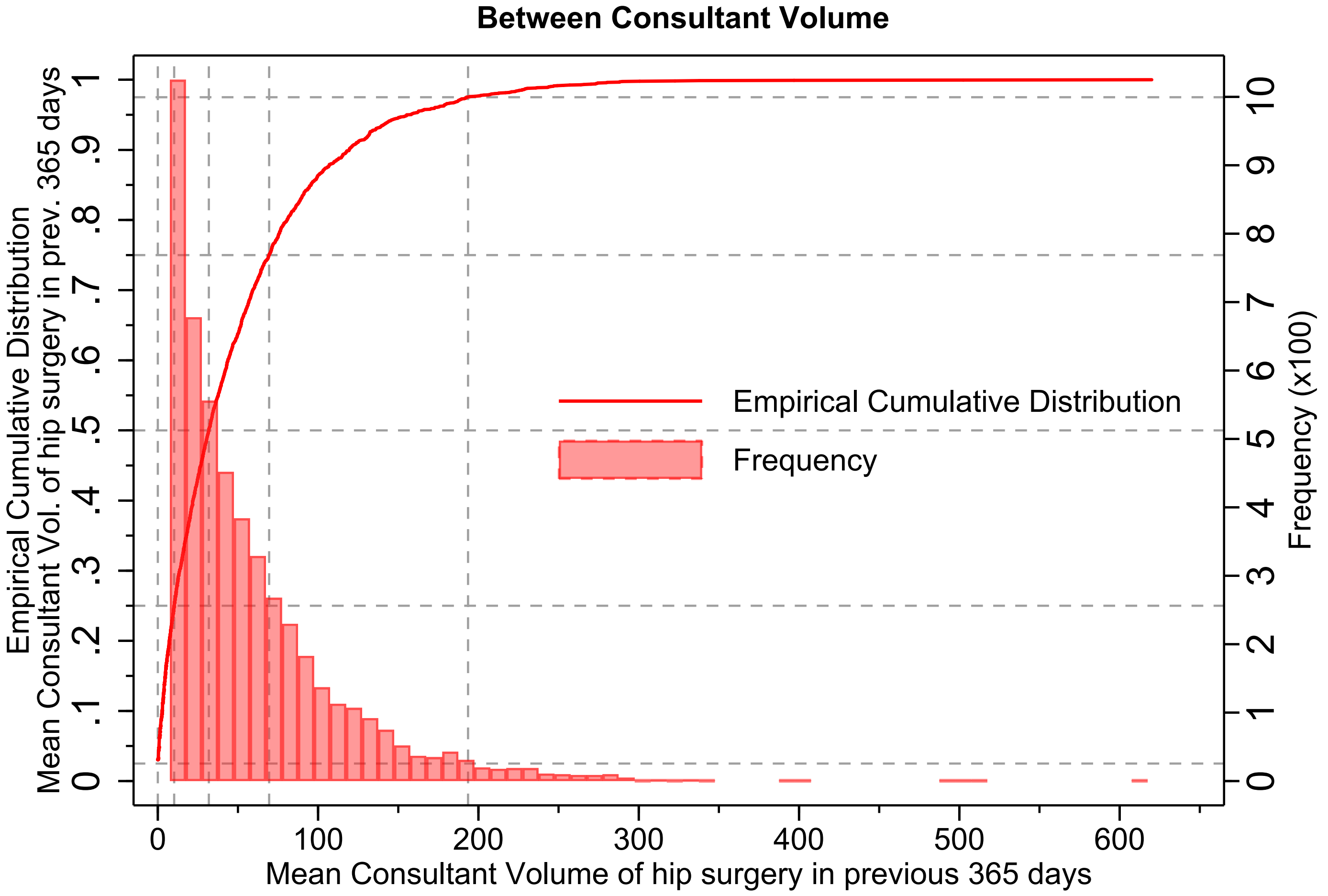

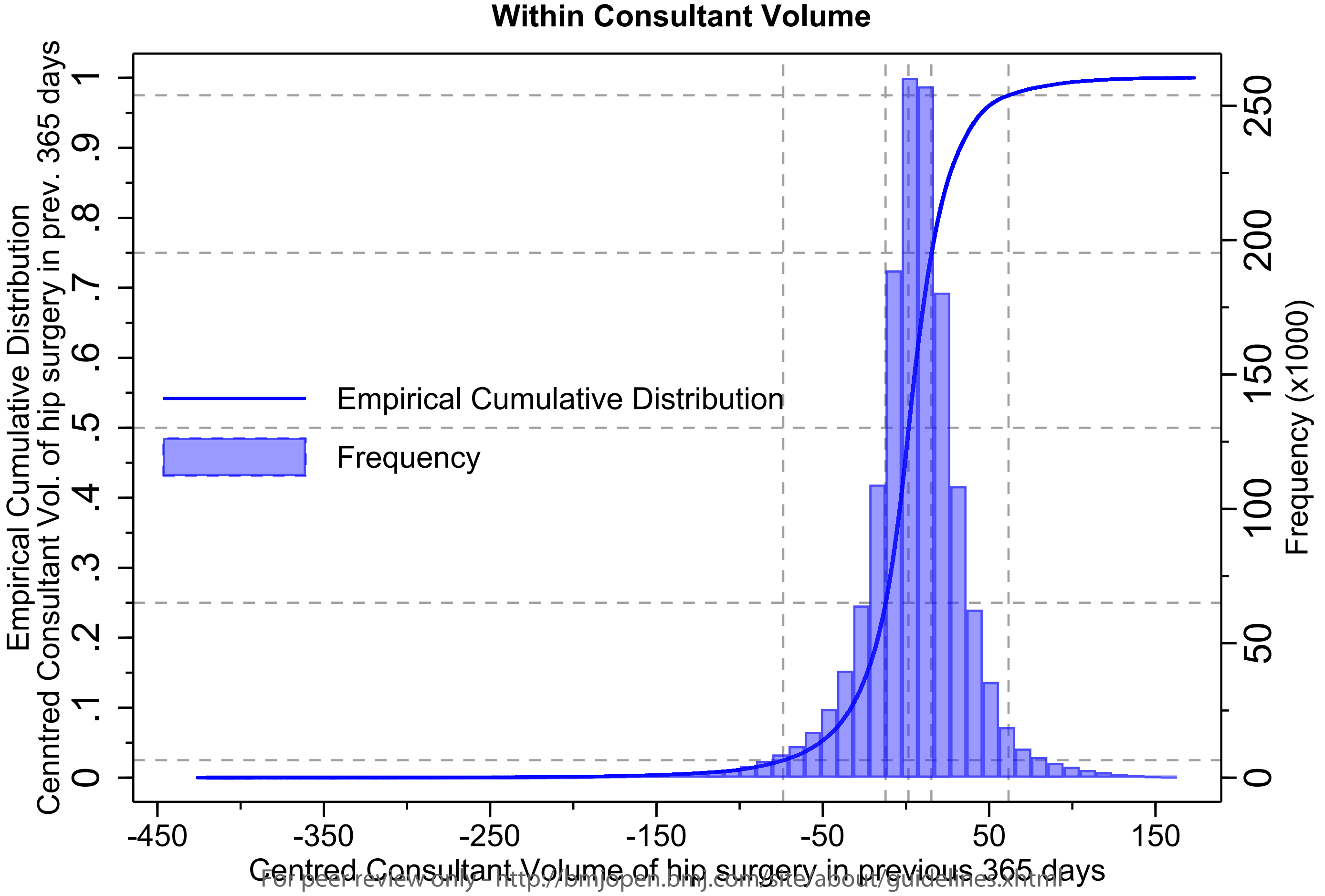

For peer review only - http://bmjopen.bmj.com/site/about/guidelines.xhtml

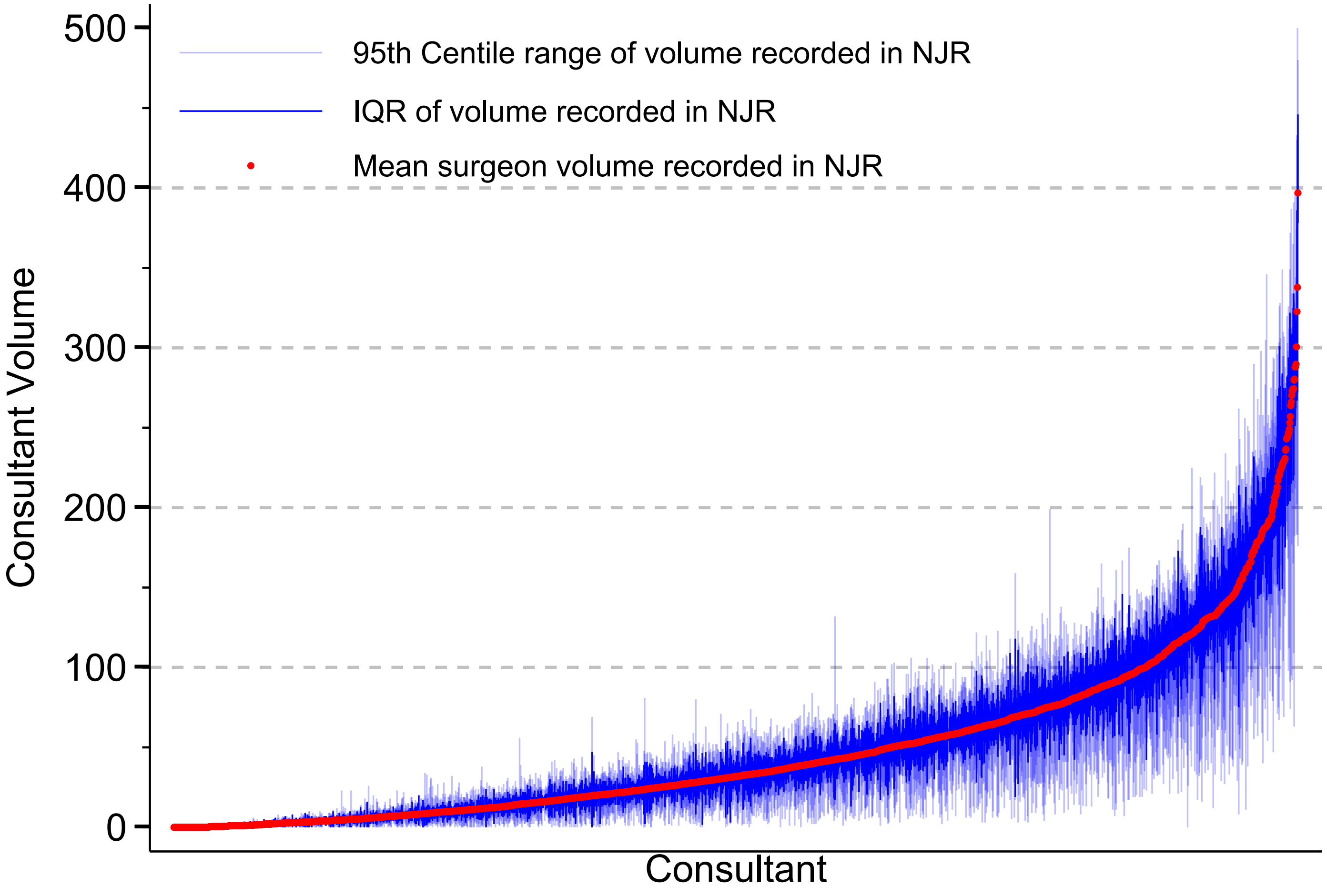
95th Centile range of volume recorded in NJR
IQR of volume recorded in NJR
Mean surgeon volume recorded in NJR
Consultant Volume
0
100
200
300
400
500
Consultant

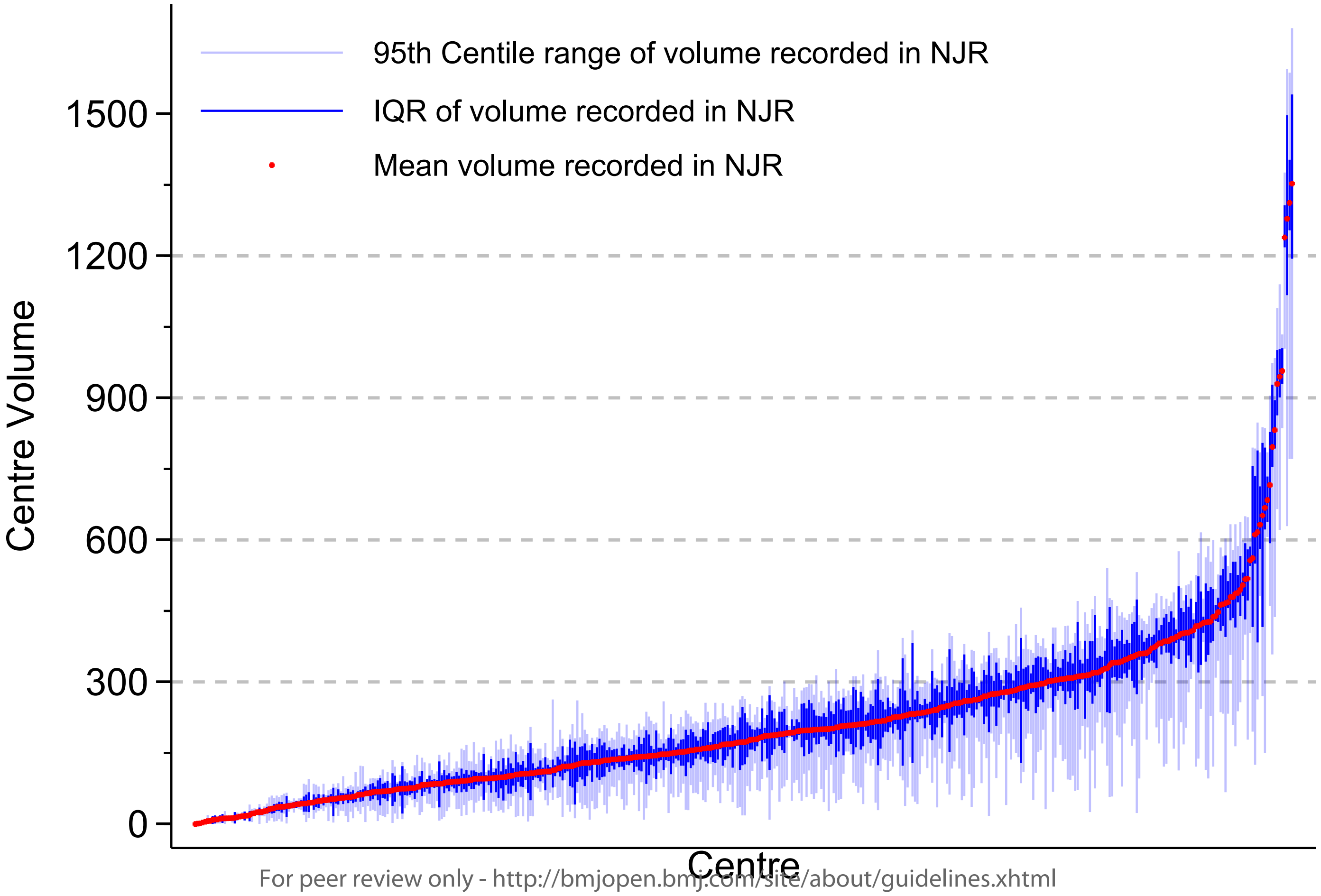
95th Centile range of volume recorded in NJR
IQR of volume recorded in NJR
Mean volume recorded in NJR
Centre Volume
0
300
600
900
1200
1500
Centre

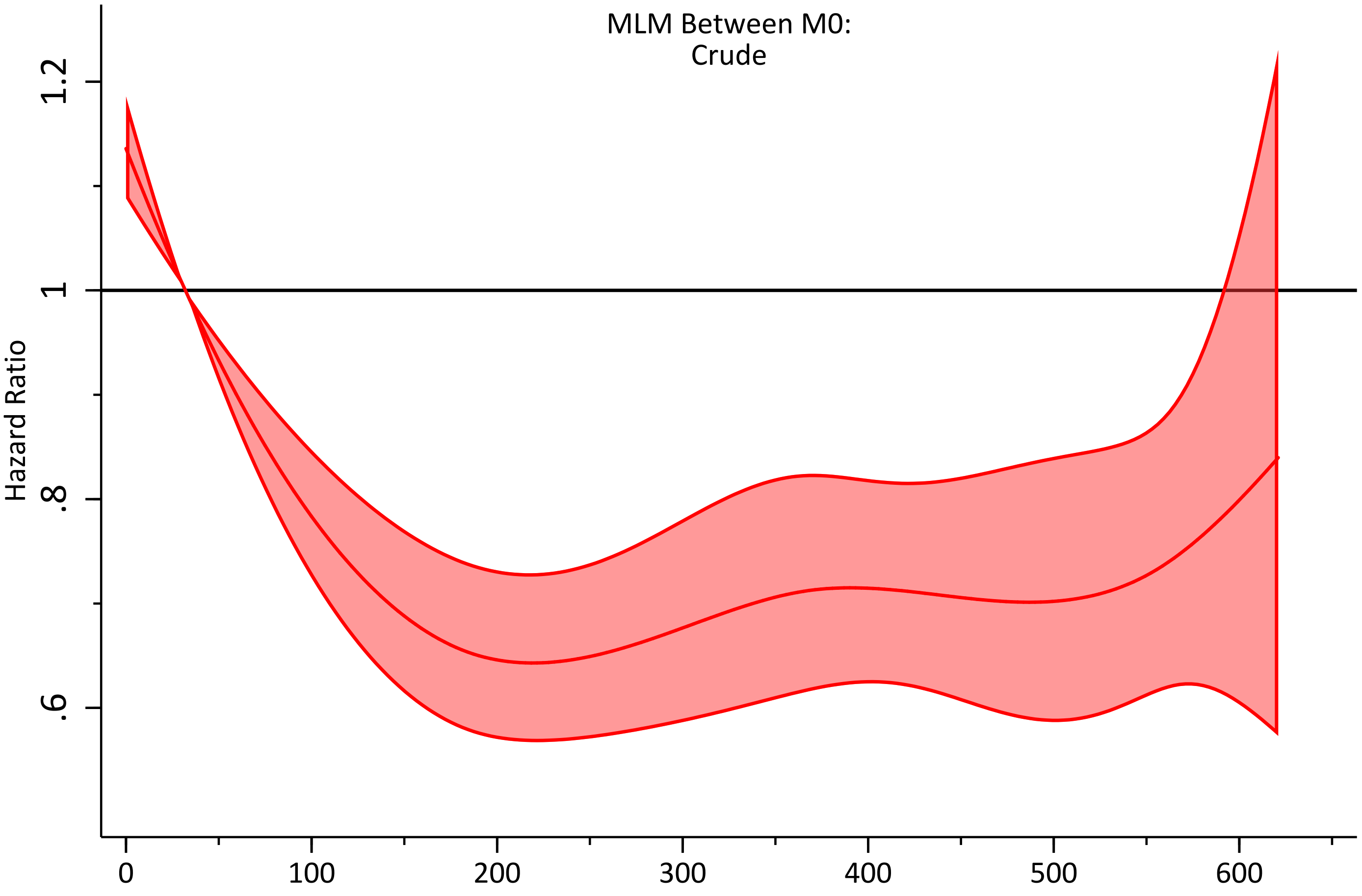

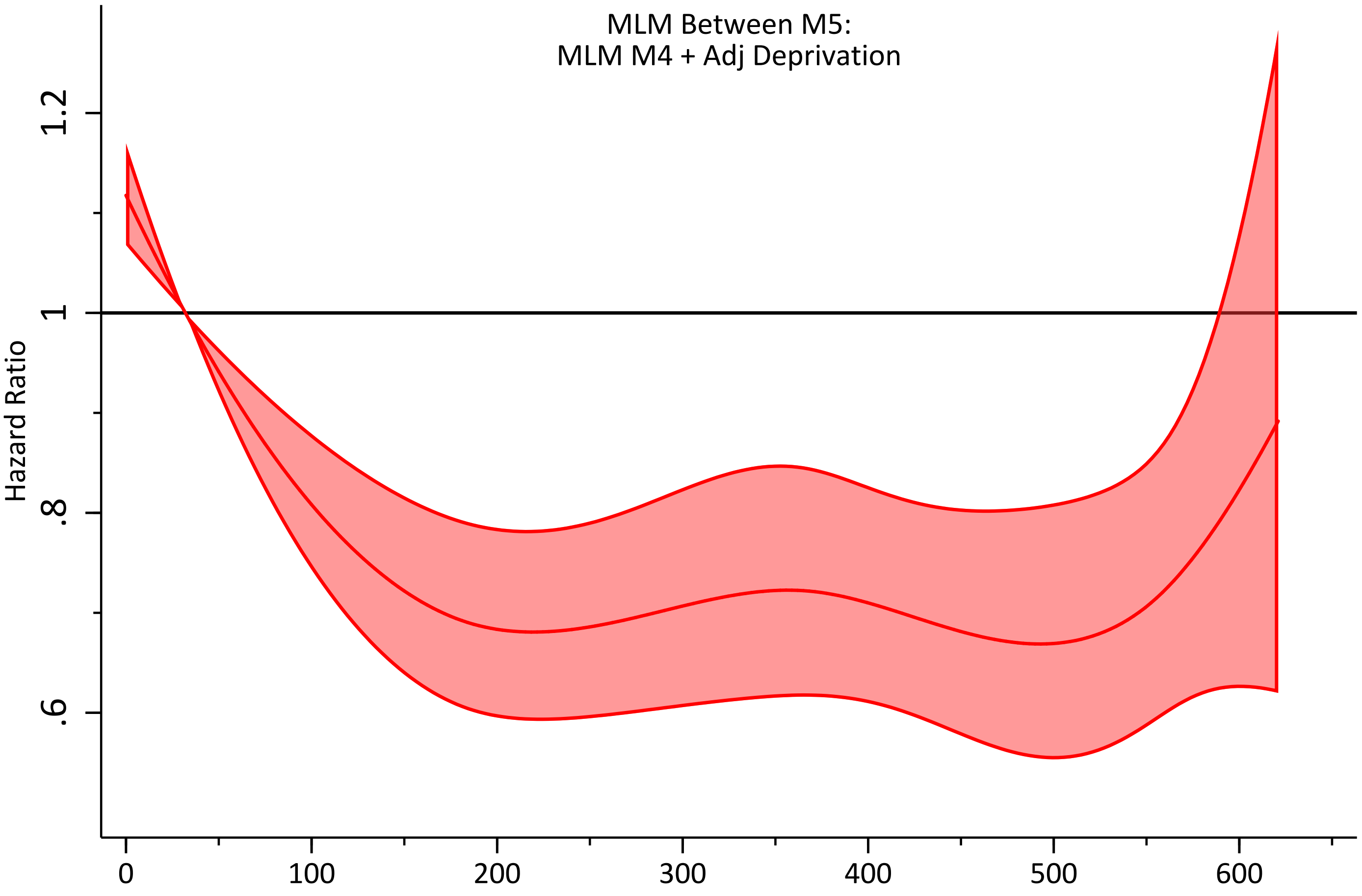

Consultant volume of hip surgery in previous 365 days

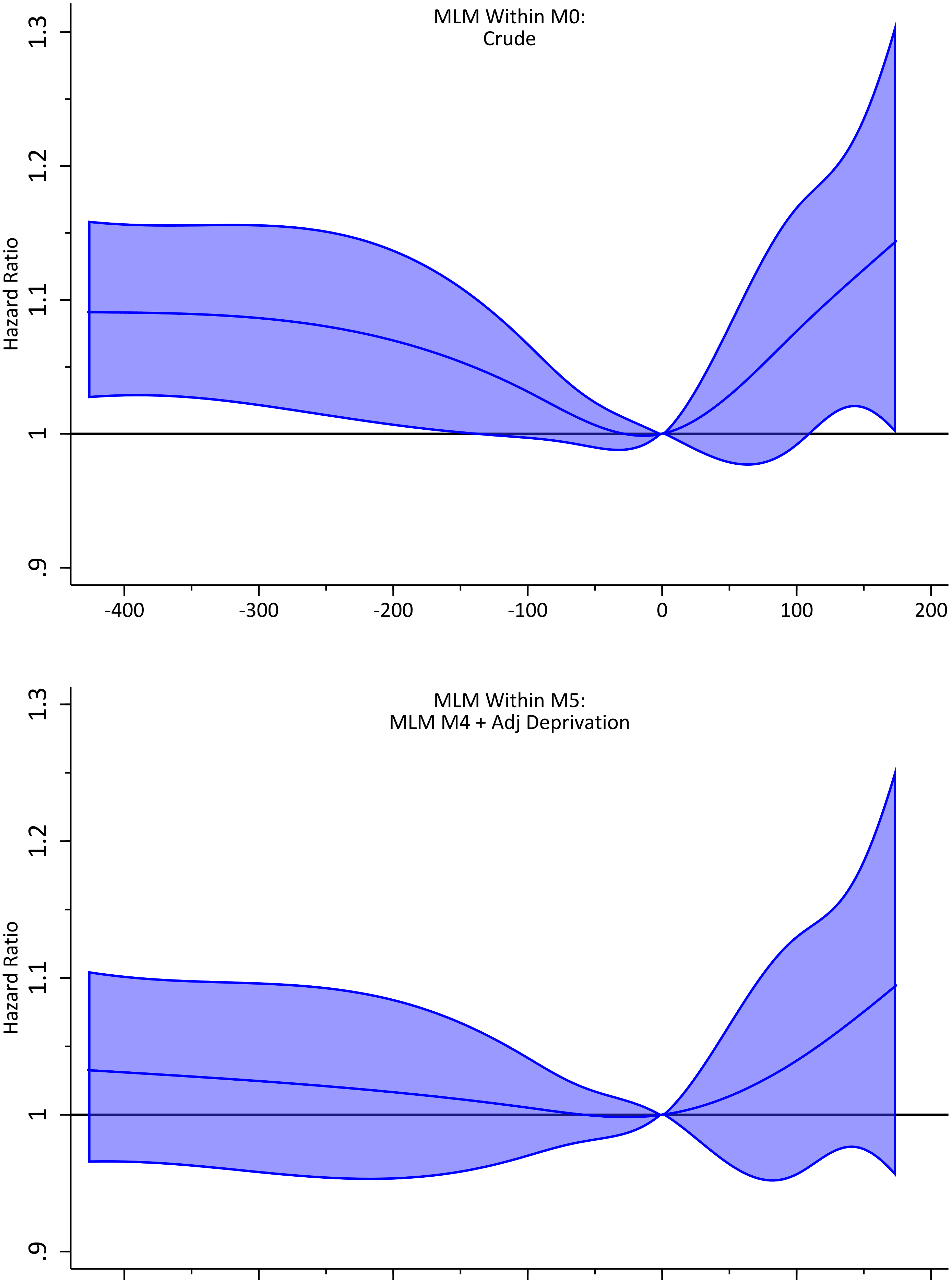

For peer review only - http://bmjopen.bmj.com/site/about/guidelines.xhtml

Consultant volume of hip surgery in previous 365 days

Supplementary Tables

Multilevel Weibull model

Denote by V_{ij} the volume in the 365 days prior to procedure i ($i = 1, \dots, I$) carried out by surgeon j ($j = 1, \dots, J$) and $\bar{V}_j$ the surgeon's average volume over the observation period. A multilevel Weibull model for the association between $h_{ij}(t)$, the hazard of revision at time t for procedure i of surgeon j, and volume is specified as

$$h_{ij}(t) = h_0(t)\exp\left[\boldsymbol{\alpha}S(\bar{V}_j, K_0) + \boldsymbol{\gamma}S\left((\bar{V}_j - V_{ij}), K_1\right) + \boldsymbol{\beta}X_{ij} + Z_j\right]$$

where $h_0(t)$ is the baseline hazard function of a Weibull distribution, $S()$ represents the restricted cubic spline basis, with a vector of K_k knots, $\boldsymbol{\alpha}$ is a vector of coefficients representing the between-consultant volume association, $\boldsymbol{\gamma}$ is a vector of coefficients for the within-consultant volume association, and $\boldsymbol{\beta}$ is a vector of coefficients for confounding factors X_{ij}, and $Z_j \sim N(0, \sigma^2)$ is a surgeon-level random effect.

Supplementary.Table 1: Coding and specification of confounding variables

Variable	Options	Baseline | Centred	Regression Specification
Patient Age	Orthogonalized Restricted Cubic Splines Knots @ knots(50(10)90 102	70	Continuous
Sex	Male / Female	Male	Binary
American Society of Anaesthesiologists (ASA) grade	I / II / III / IV+V	II	MV Binary
Operation funder	Public / Private	Public	Binary
Fixation	Cemented / Uncemented / Hybrid / Reverse Hybrid	Cemented	MV Binary
Approach	Posterior /Other	Posterior	Binary
Patient position during arthroplasty	Lateral / Supine	Lateral	Binary
Anaesthetic type	Spinal / GA / GA & Spinal / GA & Nerve Block/ Epidural / Nerve Block & Spinal / GA & Epidural / None / Epidural & Spinal / Nerve Block / GA & Nerve Block & Spinal / Other	Spinal	MV Binary
Mechanical thromboprophylaxis	TED / TED ICC / ICC / TED FP / FP / TED FP ICC / FP ICC / None	None	MV Binary
Chemical thromboprophylaxis	LMWH / Asprin/ DTI / LMWH Asprin / FXa / LMWH DTI / LMWH FXa / PS / LMWH Warf / Warf / LDH / LMWH PS /None /Other	None	MV Binary
Bearing	CoC / CoM /CoPe /MoC / MoPe / MoPu	MoPE	MV Binary
Year of arthroplasty	Orthogonalised Restricted Cubic Splines Knots @ 0(4.33)13	2004	Continuous
Setting of the treatment	NHS / Treatment Centre / Privates	NHS	MV Binary
Surgical centre volume	Orthogonalised Restricted Cubic Splines Knots @ 0(430)1720	97	Continuous

For peer review only

Training status of the primary surgeon	Consultant / Trainee	Consultant	Binary
Consultant was listed in the NJR prior to 2008	<2008 / >= 2008	<2008	Binary
proportion of THA undertaken in the NHS	Orthogonalized Restricted Cubic Splines Knots @ knots(0(0.25)1	0.7	Continuous
proportion of THA procedures	Orthogonalized Restricted Cubic Splines Knots @ knots(0(0.125)1	0.6	Continuous
Index of Multiple Deprivation (English)	Deciles	Decile #1	MV Binary
Welsh Index of Multiple Deprivation	Deciles	Decile #1	MV Binary

For peer review only

Supplementary.Table 2: Descriptive statistics of continuous variables used in the analysis.

Variable	Mean	(SD)	[25th	50th	75th]	Missing
(Traced) Age at operation date, rounded 2 DP	69.84	(10.2)	[63.56,	70.65,	77.16]	0
Consultant recorded hip volume in previous 365 days	118.36	(97.3)	[52.00,	95.00,	158.00]	0
SD consultant recorded hip volume in previous 365 days by consultant	28.13	(19.8)	[14.48,	23.48,	36.53]	114
Centre recorded hip volume in previous 365 days	370.18	(306.2)	[174.00,	284.00,	435.00]	0
SD centre recorded hip volume in previous 365 days by centre	81.84	(57.5)	[44.69,	65.90,	101.86]	2
Proportion of NHS hip procedures in previous 365 days	0.69	(0.3)	[0.54,	0.74,	0.94]	0
SD Proportion of NHS hip procedures in previous 365 day by consultant	0.10	(0.1)	[0.05,	0.10,	0.14]	114
Proportion of hip procedures in previous 365 days	0.60	(0.2)	[0.46,	0.58,	0.73]	0
SD Proportion of hip procedures in previous 365 days by consultant	0.06	(0.0)	[0.04,	0.06,	0.08]	114

For peer review only

Supplementary.Table 3: Descriptive statistics of categorical variable used in the analysis.

Variable	Factor	N	(%)
Gender	Female	397107	(61.1)
	Male	252852	(38.9)
	Missing	0	(0.0)
ASA Grade	I	94042	(14.5)
	II	457780	(70.4)
	III	95293	(14.7)
	IV +V	2844	(0.4)
	Missing	0	(0.0)
Procedure funded by:	NHS	546727	(84.1)
	Private	88588	(13.6)
	Missing	14644	(2.3)
Method of fixation	Cemented	236554	(36.4)
	Uncemented	260857	(40.1)
	Hybrid	133489	(20.5)
	Reverse Hybrid	19059	(2.9)
	Missing	0	(0.0)
Surgical Approach	Posterior	376818	(58.0)
	Other	273141	(42.0)
	Missing	0	(0.0)
Patient Position	Lateral	595964	(91.7)
	Supine	53995	(8.3)
	Missing	0	(0.0)
Anaesthetic combination	Sp	329954	(50.8)
	GA	141132	(21.7)
	GA Sp	79706	(12.3)
	GA NB	29658	(4.6)
	Epi	18759	(2.9)
	NB Sp	16450	(2.5)
	GA Epi	13235	(2.0)
	None	103	(0.0)
	Epi Sp	5449	(0.8)
	NB	3912	(0.6)
	GA NB Sp	2353	(0.4)
	Other	600	(0.1)
	Missing	8648	(1.3)
Mechanical thromboprophylaxis combination	TED	198597	(30.6)
	TED ICC	128881	(19.8)
	ICC	101050	(15.5)
	TED FP	77150	(11.9)
	FP	58467	(9.0)
	None	33814	(5.2)
	TED FP ICC	26230	(4.0)
	FP ICC	8994	(1.4)
	Missing	16776	(2.6)
Chemical thromboprophylaxis combination	LMWH	397523	(61.2)
	None	57057	(8.8)
	Asprin	44497	(6.8)
	DTI	36394	(5.6)
	LMWH Asprin	29615	(4.6)
	FXa	31768	(4.9)
	LMWH DTI	14628	(2.3)
	LMWH FXa	7391	(1.1)
	PS	4875	(0.8)

For peer review only

	LMWH Warf	3712	(0.6)
	Warf	3264	(0.5)
	LDH	1299	(0.2)
	LMWH PS	1781	(0.3)
	Other	1364	(0.2)
	Missing	14791	(2.3)
Bearing Surface [Head on Acetab.]	CoC	108539	(16.7)
	CoM	1916	(0.3)
	CoPe	119379	(18.4)
	MoC	115	(0.0)
	MoPe	419844	(64.6)
	MoPu	166	(0.0)
	Missing	0	(0.0)
Hospital provider category	NHS	420897	(64.8)
	Treatment Centre	27233	(4.2)
	Private	201829	(31.1)
	Missing	0	(0.0)
Year of operation	04	3783	(0.6)
	05	22638	(3.5)
	06	29726	(4.6)
	07	38141	(5.9)
	08	42083	(6.5)
	09	46407	(7.1)
	10	52725	(8.1)
	11	57465	(8.8)
	12	63544	(9.8)
	13	66043	(10.2)
	14	71634	(11.0)
	15	71316	(11.0)
	16	72761	(11.2)
	17	11693	(1.8)
	Missing	0	(0.0)
Operation training type (Consultant / Lead surgeon / Assisted by) Number in parentheses indicates the same or different consultant	Con.(1)/Con.(1)	520620	(80.1)
	Con.(1)/Con.(1)(?)	18202	(2.8)
	Con.(1)/Con.(2)	15041	(2.3)
	Con.(1)/Jun.(1)/Con.	37414	(5.8)
	Con.(1)/Jun.(1)/Jun.(2)	58682	(9.0)
	Missing	0	(0.0)
Consultant first recorded procedure post 2008 + 365days	Cons. Pre 2009	547791	(84.3)
	Cons. Post 2009	102168	(15.7)
	Missing	0	(0.0)
Index of Multiple Deprivation Deciles (Wales)	1	1945	(0.3)
	2	2460	(0.4)
	3	2854	(0.4)
	4	3183	(0.5)
	5	3668	(0.6)
	6	4351	(0.7)
	7	4596	(0.7)
	8	4498	(0.7)
	9	4294	(0.7)
	10	3630	(0.6)
	Missing	614480	(94.5)
Index of Multiple Deprivation Deciles (England)	1	32697	(5.0)
	2	37030	(5.7)
	3	43721	(6.7)
	4	54200	(8.3)
	5	63862	(9.8)
	6	71498	(11.0)
	7	74777	(11.5)

For peer review only

	8	76272	(11.7)
	9	78245	(12.0)
	10	80544	(12.4)
	Missing	37113	(5.7)

For peer review only

Supplementary.Table 4: Consultant volume summary statistics by potential confounding factors.

Variable	Category	N	Mean	(SD)	[25th	50th	75th]
Age at operation	50 to 55yrs	28129	129.82	(97.4)	[64.00,	108.00,	170.00]
	55 to 60yrs	48289	124.41	(95.9)	[59.00,	103.00,	164.00]
	60 to 65yrs	78757	121.97	(97.5)	[55.00,	99.00,	163.00]
	65 to 70yrs	109204	120.88	(98.6)	[53.00,	97.00,	161.00]
	70 to 75yrs	116901	118.50	(97.9)	[51.00,	95.00,	158.00]
	75 to 80yrs	104115	117.07	(98.9)	[50.00,	93.00,	156.00]
	80 to 85yrs	64385	117.64	(99.4)	[50.00,	93.00,	157.00]
	85 to 90yrs	25244	119.65	(101.7)	[51.00,	94.00,	159.00]
	>90yrs	4834	123.76	(105.2)	[53.00,	97.00,	165.00]
Gender	Female	355154	120.68	(99.3)	[53.00,	97.00,	161.00]
	Male	224704	119.45	(96.9)	[53.00,	96.00,	159.00]
ASA Grade	I	75489	129.62	(105.2)	[56.00,	103.00,	176.00]
	II	414552	120.68	(98.9)	[53.00,	97.00,	160.00]
	III	87278	110.27	(89.1)	[49.00,	88.00,	148.00]
	IV +V	2539	103.76	(80.5)	[46.00,	83.00,	140.00]
Procedure funded by:	NHS	500193	116.38	(94.5)	[52.00,	94.00,	155.00]
	Private	79665	144.25	(117.2)	[63.00,	116.00,	196.00]
Method of fixation	Cemented	209831	100.24	(73.9)	[43.00,	82.00,	140.00]
	Uncemented	231302	123.73	(100.1)	[56.00,	96.00,	160.00]
	Hybrid	121293	149.75	(124.2)	[71.00,	123.00,	192.00]
	Reverse Hybrid	17432	108.24	(68.9)	[57.00,	93.50,	147.00]
Hospital provider category	NHS	371858	113.06	(93.7)	[50.00,	90.00,	151.00]
	Treatment Centre	25296	103.59	(56.0)	[60.00,	100.00,	134.00]
	Private	182704	137.05	(109.5)	[61.00,	109.00,	184.00]
P(NHS) in previous 365-days	<0.25	59325	115.76	(109.8)	[42.00,	85.00,	145.00]
	>=0.25 & <0.5	70297	149.07	(118.0)	[60.00,	115.00,	212.00]
	>=0.5 & <0.75	170720	137.44	(100.4)	[72.00,	117.00,	178.00]
	>=0.75 & <1	278820	103.62	(84.7)	[45.00,	83.00,	140.00]
P(Hip Arthroplasty) in previous 365-days	<0.25	21128	25.62	(21.4)	[10.00,	21.00,	33.00]
	>=0.25 & <0.5	176666	66.41	(40.2)	[36.00,	59.00,	89.00]
	>=0.5 & <0.75	255607	119.99	(67.8)	[69.00,	109.00,	159.00]
	>=0.75 & <1	126204	212.03	(137.2)	[123.00,	188.00,	265.00]
Index of Multiple Deprivation Deciles (Wales)	1	1657	98.69	(78.0)	[39.00,	72.00,	148.00]
	2	2153	98.57	(74.4)	[40.00,	76.00,	142.00]
	3	2522	91.51	(67.8)	[40.00,	71.00,	129.00]
	4	2809	88.34	(68.7)	[38.00,	69.00,	121.00]
	5	3343	91.71	(72.2)	[36.00,	70.00,	130.00]
	6	3961	90.30	(67.8)	[38.00,	70.00,	130.00]
	7	4249	97.31	(70.0)	[43.00,	77.00,	138.00]
	8	4135	98.71	(70.1)	[44.00,	80.00,	139.00]

For peer review only

	9	3947	115.23	(84.7)	[49.00,	92.00,	174.00]
	10	3265	128.15	(85.1)	[56.00,	108.00,	198.00]
Index of Multiple Deprivation Deciles (England)	1	28022	98.15	(75.1)	[45.00,	81.00,	131.00]
	2	32304	101.47	(81.3)	[46.00,	83.00,	135.00]
	3	38370	106.67	(89.1)	[47.00,	85.00,	140.00]
	4	47978	111.39	(88.7)	[51.00,	90.00,	147.00]
	5	57129	118.02	(97.2)	[53.00,	94.00,	155.00]
	6	64237	124.51	(101.9)	[56.00,	100.00,	164.00]
	7	67491	125.95	(103.4)	[57.00,	101.00,	166.00]
	8	68831	127.19	(103.2)	[57.00,	103.00,	170.00]
	9	70537	130.69	(105.0)	[59.00,	106.00,	174.00]
	10	72918	134.51	(108.8)	[60.00,	111.00,	179.00]
Surgical Approach	Posterior	342849	144.30	(108.1)	[71.00,	121.00,	189.00]
	Other	237009	85.35	(68.7)	[37.00,	69.00,	115.00]
Patient Position	Lateral	535280	122.45	(99.0)	[55.00,	99.00,	164.00]
	Supine	44578	93.27	(87.1)	[40.00,	70.00,	112.00]
Anaesthetic combination	Sp	305186	116.39	(88.7)	[56.00,	98.00,	154.00]
	GA	125359	109.36	(87.5)	[46.00,	86.00,	151.00]
	GA Sp	71916	162.93	(131.8)	[68.00,	128.00,	221.00]
	GA NB	25213	105.09	(83.1)	[44.00,	84.00,	144.00]
	Epi	16568	95.02	(84.9)	[38.00,	71.00,	132.00]
	NB Sp	15451	132.51	(151.0)	[53.00,	86.00,	137.00]
	GA Epi	9820	130.50	(98.4)	[52.00,	102.00,	202.00]
	None	59	115.80	(78.9)	[49.00,	103.00,	167.00]
	Epi Sp	4041	104.14	(79.2)	[43.00,	83.00,	155.00]
	NB	3524	101.01	(83.8)	[48.00,	79.00,	133.00]
	GA NB Sp	2188	163.59	(115.9)	[78.00,	149.00,	230.00]
	Other	533	111.07	(112.9)	[44.00,	85.00,	143.00]
Mechanical thromboprophylaxis combination	TED	183510	115.60	(104.1)	[49.00,	92.00,	149.00]
	TED ICC	119415	118.20	(101.3)	[51.00,	90.00,	151.00]
	ICC	92340	109.16	(77.2)	[52.00,	91.00,	151.00]
	TED FP	67618	146.91	(118.8)	[67.00,	120.00,	187.00]
	FP	53375	132.33	(86.1)	[62.00,	122.00,	190.00]
	None	31757	117.05	(79.4)	[53.00,	100.00,	170.00]
	TED FP ICC	23445	115.61	(84.4)	[58.00,	91.00,	151.00]
	FP ICC	8398	103.40	(81.1)	[48.00,	83.00,	144.00]
Chemical thromboprophylaxis combination	LMWH	362081	113.44	(94.4)	[50.00,	91.00,	150.00]
	None	53791	129.35	(106.3)	[54.00,	99.00,	176.00]
	Asprin	38635	125.01	(88.2)	[56.00,	110.00,	170.00]
	DTI	34669	117.68	(78.4)	[59.00,	100.00,	163.00]
	LMWH Asprin	26428	146.37	(111.5)	[64.00,	125.00,	207.00]
	FXa	30026	153.06	(120.9)	[73.00,	122.00,	194.00]
	LMWH DTI	13840	138.70	(127.3)	[56.00,	99.00,	186.00]
	LMWH FXa	7019	128.35	(122.9)	[58.00,	99.00,	159.00]
	PS	4553	102.17	(64.2)	[50.00,	91.00,	144.00]
	LMWH Warf	3028	121.75	(98.5)	[55.00,	103.00,	164.00]

For peer review only

	Warf	2616	115.38	(73.6)	[57.00,	117.00,	152.00]
	LDH	677	91.82	(86.9)	[28.00,	47.00,	168.00]
	LMWH PS	1360	104.65	(77.3)	[49.00,	86.00,	160.00]
	Other	1135	120.09	(117.3)	[56.00,	88.00,	154.00]
Bearing Surface [Head on Acetab.]	CoC	89379	125.46	(90.5)	[61.00,	104.00,	166.00]
	CoM	1728	186.67	(141.7)	[69.00,	121.00,	286.50]
	CoPe	106359	138.64	(125.1)	[60.00,	104.00,	178.00]
	MoC	100	103.72	(88.3)	[41.50,	68.50,	140.50]
	MoPe	382139	113.55	(90.3)	[49.00,	92.00,	154.00]
	MoPu	153	123.27	(46.2)	[92.00,	130.00,	160.00]
Year of operation	04	1902	57.84	(47.1)	[24.00,	45.00,	77.00]
	05	12392	73.87	(60.3)	[29.00,	56.00,	101.00]
	06	16901	91.97	(81.6)	[33.00,	69.00,	124.00]
	07	22287	97.09	(83.3)	[36.00,	75.00,	133.00]
	08	37678	111.83	(98.2)	[44.00,	86.00,	149.00]
	09	44020	115.91	(102.8)	[45.00,	89.00,	151.00]
	10	50416	114.78	(95.7)	[48.00,	89.00,	155.00]
	11	54957	116.60	(98.2)	[50.00,	91.00,	154.00]
	12	60581	122.55	(99.0)	[55.00,	99.00,	163.00]
	13	62950	124.14	(94.3)	[58.00,	100.00,	169.00]
	14	68357	127.82	(94.1)	[63.00,	106.00,	171.00]
	15	68189	133.04	(103.8)	[64.00,	111.00,	172.00]
	16	69434	130.91	(106.2)	[65.00,	107.00,	169.00]
	17	9794	133.73	(106.7)	[67.00,	108.00,	175.00]
Centre Volume in previous 365 days	1x day	252914	99.94	(90.5)	[40.00,	75.00,	130.00]
	2x day	223425	116.00	(76.7)	[59.00,	101.00,	158.00]
	3x day	40127	142.01	(86.8)	[80.00,	127.00,	191.00]
	4x day	33584	226.83	(167.5)	[115.00,	183.00,	289.00]
	5x day	14127	198.14	(143.4)	[99.00,	172.00,	250.00]
	6x day	12630	151.66	(73.8)	[99.00,	133.00,	206.00]
	7x day	3051	156.37	(64.7)	[110.00,	142.00,	220.00]
Operation training type (Consultant / Lead surgeon / Assisted by) Number in parentheses indicates the same or different consultant	Con.(1)/Con.(1)	463705	117.46	(91.7)	[53.00,	95.00,	157.00]
	Con.(1)/Con.(1)(?)	16246	105.20	(115.9)	[40.00,	67.00,	133.00]
	Con.(1)/Con.(2)	13637	141.29	(134.4)	[49.00,	101.00,	197.00]
	Con.(1)/Jun.(1)/Con.	34006	110.47	(72.3)	[56.00,	101.00,	153.00]
	Con.(1)/Jun.(1)/Jun.(2)	52264	150.02	(139.3)	[58.00,	119.00,	191.00]
Consultant first recorded procedure post 2008 + 365days	Cons. Pre 2009	482572	126.19	(104.1)	[54.00,	101.00,	170.00]
	Cons. Post 2009	97286	90.53	(53.7)	[50.00,	81.00,	122.00]

For peer review only

*Supplementary.Figure 1 **Between-consultant** Marginal association of hip surgical volume in the preceding 365-days and hazard of revision arthroplasty adjusted with 4 different sets (M2 to M4) of confounding factors in a multi-level model (MLM).*

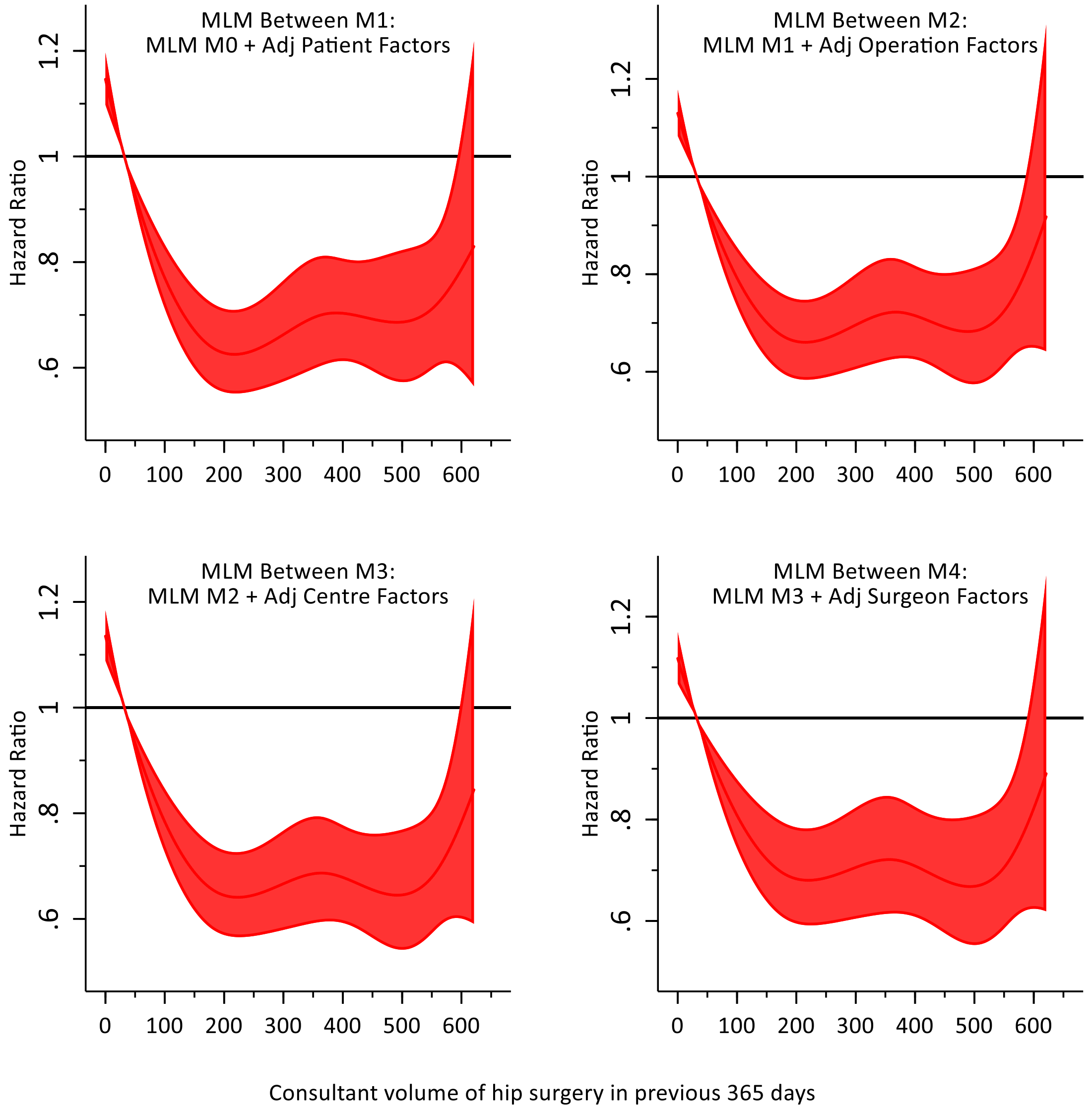

Foot Note.

Patient factors include sex, ASA grade, funder. Operation confounding factors include fixation, approach, position, anaesthetic, mechanical and chemical thromboprophylaxis, bearing, and year of operation. Centre confounding factors include hospital location and centre volume in the preceding 365-days. Surgeon confounding factors included; lead

operating surgeon, listing of a surgeon within NJR prior to 2008, the proportion of NHS cases in the preceding year, and proportion hip arthroplasty procedures undertaken in the previous year.

For peer review only

Supplementary.Figure 2 ***Within-consultant*** *Marginal association of hip surgical volume in the preceding 365-days and hazard of revision arthroplasty adjusted with 4 different sets (M2 to M4) of confounding factors in a multi-level model (MLM).*

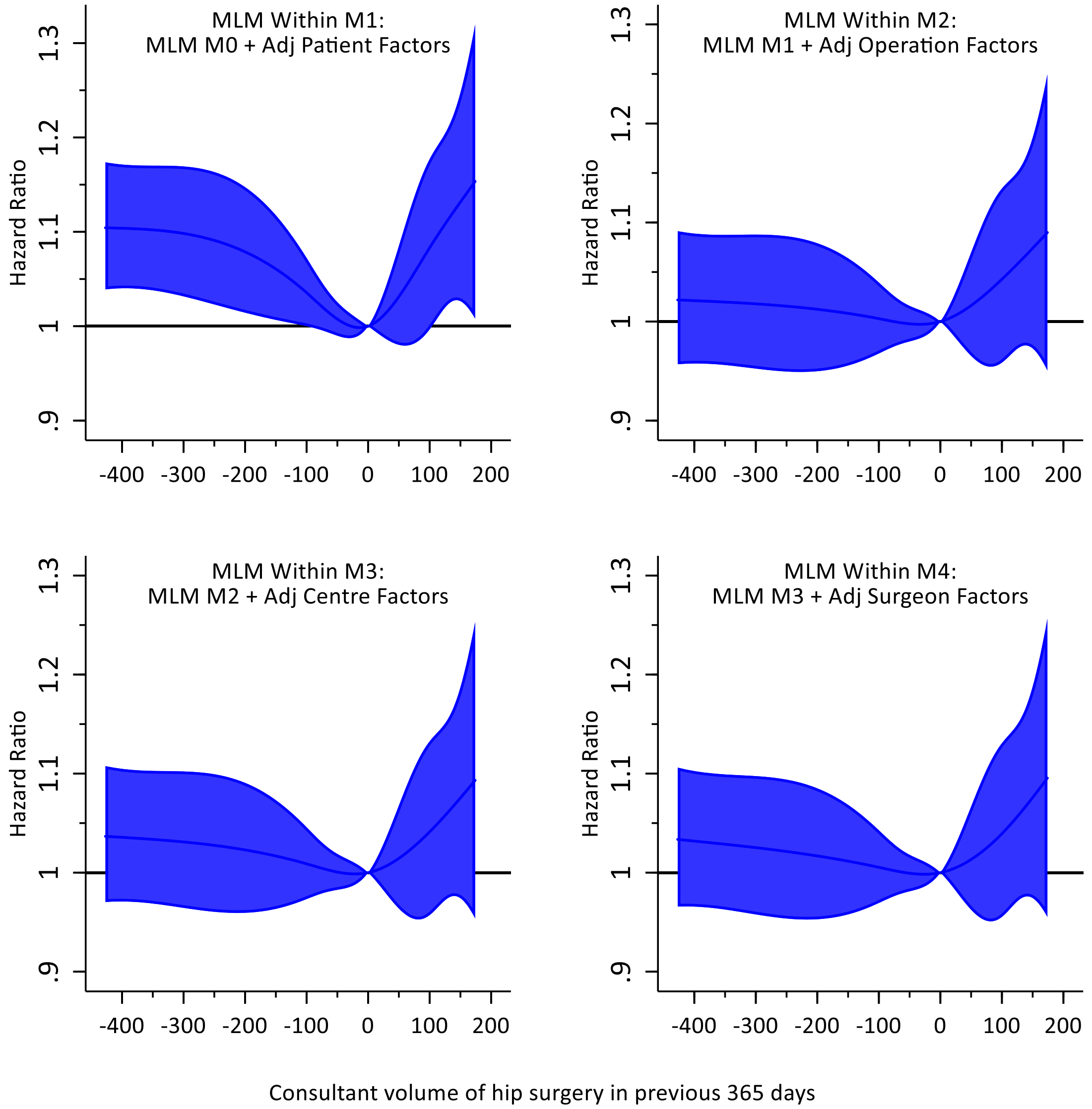

Foot Note.

Patient factors include sex, ASA grade, funder. Operation confounding factors include fixation, approach, position, anaesthetic, mechanical and chemical thromboprophylaxis, bearing, and year of operation. Centre confounding factors include hospital location and

centre volume in the preceding 365-days. Surgeon confounding factors included; lead operating surgeon, listing of a surgeon within NJR prior to 2008, the proportion of NHS cases in the preceding year, and proportion hip arthroplasty procedures undertaken in the previous year.

For peer review only

STROBE Statement—Checklist of items that should be included in reports of ***cohort studies***

	Item No	Recommendation	Page
**Title and abstract**	1	(*a*) Indicate the study's design with a commonly used term in the title or the abstract	1
		(*b*) Provide in the abstract an informative and balanced summary of what was done and what was found	2
**Introduction**			
Background/rationale	2	Explain the scientific background and rationale for the investigation being reported	4
Objectives	3	State specific objectives, including any prespecified hypotheses	5
**Methods**			
Study design	4	Present key elements of study design early in the paper	6
Setting	5	Describe the setting, locations, and relevant dates, including periods of 6recruitment, exposure, follow-up, and data collection	6
Participants	6	(*a*) Give the eligibility criteria, and the sources and methods of selection of participants. Describe methods of follow-up	6
		(*b*) For matched studies, give matching criteria and number of exposed and unexposed	
Variables	7	Clearly define all outcomes, exposures, predictors, potential confounders, and effect modifiers. Give diagnostic criteria, if applicable	7
Data sources/ measurement	8*	For each variable of interest, give sources of data and details of methods of assessment (measurement). Describe comparability of assessment methods if there is more than one group	*7/8*
Bias	9	Describe any efforts to address potential sources of bias	7/8
Study size	10	Explain how the study size was arrived at	8
Quantitative variables	11	Explain how quantitative variables were handled in the analyses. If applicable, describe which groupings were chosen and why	8 sup.t1
Statistical methods	12	(*a*) Describe all statistical methods, including those used to control for confounding	8
		(*b*) Describe any methods used to examine subgroups and interactions	n/a
		(*c*) Explain how missing data were addressed	9
		(*d*) If applicable, explain how loss to follow-up was addressed	7
		(*e*) Describe any sensitivity analyses	n/a
**Results**			
Participants	13*	(a) Report numbers of individuals at each stage of study—eg numbers potentially eligible, examined for eligibility, confirmed eligible, included in the study, completing follow-up, and analysed	Fig 1
		(b) Give reasons for non-participation at each stage	Fig 1
		(c) Consider use of a flow diagram	Fig 1
Descriptive data	14*	(a) Give characteristics of study participants (eg demographic, clinical, social) and information on exposures and potential confounders	ST2/3
		(b) Indicate number of participants with missing data for each variable of interest	Fig 1
		(c) Summarise follow-up time (eg, average and total amount)	6
Outcome data	15*	Report numbers of outcome events or summary measures over time	10
Main results	16	(*a*) Give unadjusted estimates and, if applicable, confounder-adjusted estimates and their precision (eg, 95% confidence interval). Make clear	Fig 4/5

For peer review only - http://bmjopen.bmj.com/site/about/guidelines.xhtml

		which confounders were adjusted for and why they were included	
		(*b*) Report category boundaries when continuous variables were categorized	n/a
		(*c*) If relevant, consider translating estimates of relative risk into absolute risk for a meaningful time period	n/a
Other analyses	17	Report other analyses done—eg analyses of subgroups and interactions, and sensitivity analyses	n/a
**Discussion**			
Key results	18	Summarise key results with reference to study objectives	12
Limitations	19	Discuss limitations of the study, taking into account sources of potential bias or imprecision. Discuss both direction and magnitude of any potential bias	13
Interpretation	20	Give a cautious overall interpretation of results considering objectives, limitations, multiplicity of analyses, results from similar studies, and other relevant evidence	14
Generalisability	21	Discuss the generalisability (external validity) of the study results	15
**Other information**			
Funding	22	Give the source of funding and the role of the funders for the present study and, if applicable, for the original study on which the present article is based	16

*Give information separately for exposed and unexposed groups.

Note: An Explanation and Elaboration article discusses each checklist item and gives methodological background and published examples of transparent reporting. The STROBE checklist is best used in conjunction with this article (freely available on the Web sites of PLoS Medicine at http://www.plosmedicine.org/, Annals of Internal Medicine at http://www.annals.org/, and Epidemiology at http://www.epidem.com/). Information on the STROBE Initiative is available at http://www.strobe-statement.org.

For peer review only
